# Genetic variation in *TERT* modifies the risk of hepatocellular carcinoma in alcohol-related cirrhosis: results from a genome-wide case-control study

**DOI:** 10.1136/gutjnl-2022-327196

**Published:** 2022-07-04

**Authors:** Stephan Buch, Hamish Innes, Philipp Ludwig Lutz, Hans Dieter Nischalke, Jens U Marquardt, Janett Fischer, Karl Heinz Weiss, Jonas Rosendahl, Astrid Marot, Marcin Krawczyk, Markus Casper, Frank Lammert, Florian Eyer, Arndt Vogel, Silke Marhenke, Johann von Felden, Rohini Sharma, Stephen Rahul Atkinson, Andrew McQuillin, Jacob Nattermann, Clemens Schafmayer, Andre Franke, Christian Strassburg, Marcella Rietschel, Heidi Altmann, Stefan Sulk, Veera Raghavan Thangapandi, Mario Brosch, Carolin Lackner, Rudolf E Stauber, Ali Canbay, Alexander Link, Thomas Reiberger, Mattias Mandorfer, Georg Semmler, Bernhard Scheiner, Christian Datz, Stefano Romeo, Stefano Ginanni Corradini, William Lucien Irving, Joanne R Morling, Indra Neil Guha, Eleanor Barnes, M Azim Ansari, Jocelyn Quistrebert, Luca Valenti, Sascha A Müller, Marsha Yvonne Morgan, Jean-François Dufour, Jonel Trebicka, Thomas Berg, Pierre Deltenre, Sebastian Mueller, Jochen Hampe, Felix Stickel

**Affiliations:** 1 Department of Medicine I, Dresden University Hospital, Dresden, Germany; 2 Center for Regenerative Therapies Dresden (CRTD), Technische Universität Dresden (TU Dresden), Dresden, Germany; 3 School of Health and Life Sciences, Glasgow Caledonian University School of Health and Life Sciences, Glasgow, UK; 4 NIHR Nottingham Biomedical Research Centre, Nottingham University Hospitals NHS Trust, Nottingham, UK; 5 Department of Internal Medicine I, University of Bonn, Bonn, Germany; 6 Department of Medicine I, University of Luebeck Human Medicine, Lubeck, Germany; 7 Department of Gastroenterology and Rheumatology, Section Hepatology, Leipzig University, Leipzig, Germany; 8 Department of Internal Medicine, Krankenhaus Salem, Heidelberg, Germany; 9 Department of Gastroenterology, University Hospital Halle, Halle, Germany; 10 Division of Gastroenterology and Hepatology, Centre Hospitalier Universitaire Vaudois, Lausanne, Switzerland; 11 Department of Gastroenterology and Hepatology, CHU UCL Namur, Université catholique de Louvain, Louvain-la-Neuve, Belgium; 12 Department of Medicine II, Saarland University Medical Center, Saarland University, Saarbrucken, Germany; 13 Laboratory of Metabolic Liver Diseases, Department of General, Transplant and Liver Surgery, Centre for Preclinical Research, Medical University of Warsaw, Warszawa, Poland; 14 Department of Clinical Toxicology, Klinikum Rechts der Isar, Technical University of Munich, Munchen, Germany; 15 Department of Gastroenterology, Hepatology and Endocrinology, Hannover Medical School, Hannover, Germany; 16 Department of Internal Medicine, University Medical Center Hamburg-Eppendorf, Hamburg, Germany; 17 Hammersmith Hospital Campus, Imperial College, London, UK; 18 Molecular Psychiatry Laboratory, University College London, London, UK; 19 Department of General Surgery, Rostock University Medical Center, Rostock, Germany; 20 Institute for Clinical Molecular Biology, Kiel University, Kiel, Germany; 21 Department of Genetic Epidemiology in Psychiatry, Central Institute of Mental Health, Medical Faculty Mannheim, Heidelberg University, Heidelberg, Germany; 22 Department of Medicine I, University Hospital Dresden, Dresden, Germany; 23 Institute of Pathology, University of Graz, Graz, Austria; 24 Department of Internal Medicine, University of Graz, Graz, Austria; 25 Department of Internal Medicine, Ruhr-Universitat Bochum, Bochum, Germany; 26 Department of Gastroenterology, Hepatology and Infectious Diseases, Otto von Guericke Universitat Magdeburg, Magdeburg, Germany; 27 Division of Gastroenterology & Hepatology, Department of Internal Medicine III, Medical University of Vienna, Wien, Austria; 28 Department of Internal Medicine, General Hospital Oberndorf, Paracelsus Medical University Salzburg, Salzburg, Austria; 29 Department of Molecular and Clinical Medicine, University of Gothenburg, Institute of Medicine, Sahlgrenska Academy, Wallenberg Laboratory, Gothenburg, Sweden; 30 Clinical Nutrition Unit, Department of Medical and Surgical Sciences, Magna Graecia University of Catanzaro, Catanzaro, Italy; 31 Division of Gastroenterology, Department of Translational and Precision Medicine, University of Rome La Sapienza, Rome, Italy; 32 Microbiology, University of Nottingham, Nottingham, UK; 33 Division of Epidemiology and Public Health, University of Nottingham, Nottingham, UK; 34 Nottingham Digestive Diseases NIHR Biomedical Research Unit, University Hospital, Nottingham, UK; 35 Nuffield Department of Medicine, University of Oxford, Oxford, UK; 36 Peter Medawar Building for Pathogen Research, Nuffield Department of Medicine and the Oxford NIHR Biomedical Research Centre, University of Oxford, Oxford, UK; 37 Internal Medicine and Metabolic Diseases, Fondazione IRCCS Ca' Granda Ospedale Maggiore Policlinico, Milan, Italy; 38 Department of Surgery, Hirslanden Klinik Beau-Site, Bern, Switzerland; 39 Division of Medicine, Royal Free Campus, UCL Institute for Liver and Digestive Health, London, UK; 40 Department of BioMedical Research, University of Bern, Bern, Switzerland; 41 Gastroenterology, Hepatology, Endocrinology and Clinical Infectiology, University of Münster, Münster, Germany; 42 Division of Hepatology, Department of Medicine II, Leipzig University Medical Center, Leipzig University, Leipzig, Germany; 43 Salem Medical Center, Department of Gastroenterology and Hepatology, University of Heidelberg, Heidelberg, Germany; 44 Department of Gatroenterology and Hepatology, University of Zürich, Zürich, Switzerland; 45 Hirslanden Klinik Beau-Site, Bern, Switzerland

**Keywords:** hepatocellular carcinoma, genetic polymorphisms

## Abstract

**Objective:**

Hepatocellular carcinoma (HCC) often develops in patients with alcohol-related cirrhosis at an annual risk of up to 2.5%. Some host genetic risk factors have been identified but do not account for the majority of the variance in occurrence. This study aimed to identify novel susceptibility loci for the development of HCC in people with alcohol related cirrhosis.

**Design:**

Patients with alcohol-related cirrhosis and HCC (cases: n=1214) and controls without HCC (n=1866), recruited from Germany, Austria, Switzerland, Italy and the UK, were included in a two-stage genome-wide association study using a case–control design. A validation cohort of 1520 people misusing alcohol but with no evidence of liver disease was included to control for possible association effects with alcohol misuse. Genotyping was performed using the InfiniumGlobal Screening Array (V.24v2, Illumina) and the OmniExpress Array (V.24v1-0a, Illumina).

**Results:**

Associations with variants rs738409 in *PNPLA3* and rs58542926 in *TM6SF2* previously associated with an increased risk of HCC in patients with alcohol-related cirrhosis were confirmed at genome-wide significance. A novel locus rs2242652(A) in *TERT* (telomerase reverse transcriptase) was also associated with a decreased risk of HCC, in the combined meta-analysis, at genome-wide significance (p=6.41×10^−9^, OR=0.61 (95% CI 0.52 to 0.70). This protective association remained significant after correction for sex, age, body mass index and type 2 diabetes (p=7.94×10^−5^, OR=0.63 (95% CI 0.50 to 0.79). Carriage of rs2242652(A) in *TERT* was associated with an increased leucocyte telomere length (p=2.12×10^−44^).

**Conclusion:**

This study identifies rs2242652 in *TERT* as a novel protective factor for HCC in patients with alcohol-related cirrhosis.

**What is already known on this subject?**

- Hepatocellular carcinoma (HCC) is the most common primary malignancy of the liver, responsible for ~0.8M deaths per year worldwide. Most alcohol-related HCCs develop in patients with established alcohol-related cirrhosis (ArC).
- Older age, male sex, obesity and type 2 diabetes are risk factors for the development of HCC in people with ArC.
- Only three genetic loci*—PNPLA3*, *TM6SF2* and *WNT3A-WNT9A*—have been associated with the development of alcohol-related HCC, at genome-wide significance, to date. Other risk loci are likely to exist.

**What are the new findings?**

- We identify the rs2242652 germline variant in *TERT* as a novel susceptibility locus for HCC development in ArC.
- Specifically, the rs2242652 A allele is associated with an decreased risk of HCC development in ArC.
- Carriage of rs2242652 in *TERT* is not associated with the risk for developing ArC.

**HOW MIGHT IT IMPACT ON CLINICAL PRACTICE IN THE FORESEEABLE FUTURE?**

- Exploration of the functional significance of TERT variants could provide important insights into the pathogenesis of HCC in people with ArC.
- Genetic profiling of patients with ArC might inform HCC screening programmes.

## Introduction

Hepatocellular carcinoma (HCC) is the most common primary liver malignancy worldwide and is responsible for ~0.8 million deaths per annum.^1^ The global incidence of HCC is rising and may surpass 1 million cases annually by 2025.^2^ Alcohol-related liver disease (ArLD) is a leading underlying cause of HCC in Europe and Northern America.^3 4^ Most cases of alcohol-related HCC develop in patients with established cirrhosis. Cohort studies indicate that the cumulative incidence of HCC approaches 2.5% per annum for alcohol-related cirrhosis (ArC) patients attending specialist care centres.^3 4^ Clinical risk factors for the development of HCC in people with ArC include older age, male sex, type 2 diabetes and obesity^2 5^—but explain only a fraction of the total variability in HCC occurrence.^6 7^

In recent years, interest has focused on dissecting the underlying host genetics of HCC through candidate gene association studies. In the studies undertaken to date, loci in the genes coding for patatin-like phospholipase domain containing 3 (*PNPLA3*; rs738409) and transmembrane 6 superfamily member 2 (*TM6SF2;* rs58542926) were robustly confirmed to increase the risk of developing HCC in ArC,^8^ while loci, rs72613567:TA in hydroxysteroid 17-beta dehydrogenase 13 (*HSD17B13*) and rs429358:C in apolipoprotein E (*APOE*), were found to attenuate risk.^9–11^ As the products of these genes are involved in lipid turnover and processing, it is not surprising that the same loci also modulate the risk for HCC development in people with non-alcoholic fatty liver disease (NAFLD).^12^

The variants currently identified as associated with HCC risk in ArC only account for a small proportion of the heritability risk, suggesting the existence of additional genetic modulators.^7 8^ Also, the genetic risk loci recognised hitherto do not relate to genes considered pivotal to hepatocarcinogenesis.^13^ Identifying these additional, potential genetic modulators of hepatocarcinogenesis requires large genome-wide association studies (GWASs) in which cases are defined as people with ArC with HCC and controls as people with ArC who have no evidence of HCC. These definitions are critical to enable the detection of risk loci with a direct molecular link to hepatocarcinogenesis per se, rather than to the development of alcohol-related steatosis, inflammation or fibrosis.

A European GWAS of HCC in ArLD, while not conforming to this exact design, was recently undertaken by Trépo *et al*.^14^ In their discovery analysis comparing 775 HCC cases (80% with F3/F4 fibrosis) against 1332 non-HCC controls (94% with F3/F4 fibrosis), a genome-wide significant association was identified between the rs708113:T allele locus near W*NT3A-WNT9A* and a reduction in the risk for developing alcohol-related HCC.^14^

The aim of his study was to undertake a GWAS in patients with HCC against a background of ArC comprising 1066 cases and 844 controls using a case–control design.

## Methods

### Patient cohorts

#### Germany/Switzerland/Austria Alcohol Cohort (discovery cohort)

The diagnosis of ArC was established based on a history of long-term, sustained alcohol intake of a minimum of 40 g/day in women and 60 g/day in men, together with histological examination of liver tissue; or compatible historical, clinical, laboratory, radiological and endoscopic features. Patients were excluded if they had any other potential cause of liver injury, specifically if they were positive for hepatitis B surface antigen (HBsAg), anti-hepatitis C IgG (anti-hepatitis C virus (HCV) IgG), antinuclear antibodies (titre >1:80) or antimitochondrial antibodies (titre >1:40), had elevated serum ferritin levels with a transferrin saturation of >50%, a serum ceruloplasmin of <20 mg/dL (0.2 g/dL), a serum alpha-1 antitrypsin of <70 mg/dL (13 µmol/L) or were morbidly obese. The diagnosis of HCC was made following on histological examination of tumour tissue or based on criteria applied to images obtained using multiphasic CT or dynamic contrast-enhanced MRI^15 16^ (online supplemental methods A).

10.1136/gutjnl-2022-327196.supp1 Supplementary data

#### UK Alcohol Cohort (replication cohort 1)

The UK Biobank (UKB) is a large-scale biomedical database containing in depth genetic and health information from a prospective study of approximately half a million middle-aged individuals from the UK recruited in 2006–2010.^17^ Participants have been deeply phenotyped and are linked to UK hospital in-patient, cancer and mortality registries. A nested case–control dataset (n=860) was created using this resource. Cases were defined as participants with a hospital admission for ArC International Classification of Diseases 10 (ICD10:K70.3), and an HCC diagnosis (ICD10:C22.0 or ICD9:155.0). Controls were participants with a hospital admission for ArC but with no record of an HCC diagnosis. Analyses were restricted to participants of white British ancestry. These nested case–control data were then pooled with 306 patients recruited from the Centre for Hepatology at the Royal Free Hospital, London who had histologically proven ArC with or without HCC, as described previously^18^ (online supplemental methods B).

#### Germany and Italy Alcohol Cohort (replication cohort 2)

The replication cohort included 238 patients with ArC (42 with HCC) from the University of Bonn, and 72 patients with ArC (36 with HCC) from the University of Milan.

### Validation cohorts

Patients with a history of alcohol misuse (AM) but without evidence of significant alcohol-related liver injury were recruited from psychiatric units in Germany (n=1080)^19 20^ and from Hepatology Centres in Heidelberg, Germany (n=99) and London, UK (n=341)^18^ (online supplemental methods C).

### Genotyping and imputation

#### Discovery cohort

Genotyping was performed using genomic DNA extracted from peripheral blood samples as described previously.^18^ The GWAS (stage 1) included 1910 patients with ArC genotyped on the InfiniumGlobal Screening Array (V.24v2, Illumina) (table 1) (online supplemental methods D). Genotype imputation was performed with Minimac4 to the Haplotype Reference Consortium reference panel (HRC r1.1)^21^ using the Michigan Imputation Server^22^ (online supplemental methods E).

**Table 1:** Overview of the study populations included in the discovery and replication cohorts

Variable	Discovery (GWAS stage 1)* (n=1910)			Replication (stage 2)* (n=1170)									Validation† Patients with alcohol misuse (n=1520)	
	Germany-Switzerland-Austria (n=1910)			UK (cohort 1) (n=860)			Germany (cohort 2) (n=238)			Italy (cohort 3) (n=72)			Germany (n=1179)	UK (n=341)
	Cases (n=1066)	Controls (n=844)	‡P value	Cases (n=70)	Controls (n=790)	‡P value	Cases (n=42)	Controls (n=196)	P value‡	Cases (n=36)	Controls (n=36)	‡P value	Non-cirrhosis controls	Non-cirrhosis controls
Age (yr)	64.8 (8.5) (100%)	57.1 (9.7) (100%)	<0.0001	60.2 (5.9) (100%)	56.3 (8.9) (100%)	<0.0001	67.1 (9.1) (100%)	58.5 (9.7) (100%)	>0.05	72.7 (8.0) (100%)	53.3 (8.9) (100%)	<0.0001	42.7 (10.4) (100%)	48.6 (10.5) (100%)
Proportion male (n: %)	968 (90.8)	624 (73.9)	<0.0001	67 (95.7)	577 (73.0)	<0.0001	35 (83.3)	131 (66.8)	<0.05	32 (88.9)	31 (86.1)	>0.05	1148 (97.4)	263 (77.1)
BMI (kg/m^2^) §	28.1 (4.8) (69%)	26.5 (5.3) (91%)	<0.0001	29.3 (4.4) (100%)	27.5 (4.8) (92%)	<0.05	24.8 (3.5) (57%)	26.1 (5.8) (52%)	>0.05	27.0 (3.8) (64%)	27.0 (6.8) (75%)	>0.05	25.3 (4.5) (81%)	24.7 (2.3) (53%)
BMI kg/m^2^; (n: %) <25	183 (24.7)	308 (40.3)	<0.0001	13 (18.6)	227 (31.3)	<0.05	13 (54.2)	52 (51.0)	>0.05	6 (26.1)	13 (48.1)	>0.05	505 (52.6)	94 (52.2)
25–30	333 (45.0)	295 (38.6)		27 (38.6)	288 (39.7)		8 (33.3)	27 (26.5)		12 (52.2)	7 (25.9)		345 (35.9)	86 (47.8)
>30	224 (30.3)	162 (21.2)		30 (42.9)	211 (29.1)		3 (12.5)	23 (22.5)		5 (21.7)	7 (25.9)		110 (11.5)	0 (0)
Diabetes type II + (n: %)§	337 (47.1) (67%)	136 (30.1) (54%)	<0.0001	20 (28.6) (100%)	102 (12.9) (100%)	<0.0001	14 (36.8) (90%)	36 (19.7) (93%)	<0.05	16 (44.4) (100%)	5 (13.9) (100%)	<0.05	58 (5.7) (86%)	8 (3.6) (66%)

*Cases and controls were assigned to groups as detailed in the Methods section.

†Validation cohorts were used in post hoc risk assessment.

‡P values were calculated from Student’s t-test for quantitative variables and as Pearson’s χ2 test for categorical variables.

§Data are reported as mean±SD or as number (%); Completeness of phenotypic information for age, BMI and type 2 diabetes status are reported as percentage of subjects with available information below the mean value.

BMI, body mass index; GWAS, Genome-Wide Association Study.

#### Replication and validation samples

Patients from the Royal Free Hospital, London and Germany were genotyped using the OmniExpress array (24v1-0a, Illumina).^12^ The replication (stage 2) included 1170 patients with ArC (table 1). Patients from Italy were genotyped on the InfiniumGlobal Screening Array (24v2, Illumina) (online supplemental methods D). Genotypic data were imputed for each cohort to the HRC reference (online supplemental methods E). Imputed genotypic data from 606 patients were obtained from the UKB Resource.^23^

### Statistical analyses

#### GWAS analysis

Association analyses for 7 946 762 variants were performed using Plink V.2.0^24^ with allele dosages obtained after imputation (imputation info score >0.3, minor allele frequency >1%). The lambda inflation factor λ_GC_ for the unadjusted GWAS analysis was 1.085 indicative of subtle population stratification. To account for the observed inflation, the top 20 principal components (PCs) on the LD-pruned data set were calculated and the top 15 PCs of genetic ancestry included as covariates in the regression models.^25^ The corrected λ_GC_ was 1.03. Two discovery GWAS analyses were performed: GWAS 1 (primary GWAS analysis): included only the top 15 PCs as covariates in the regression model. The p value threshold for lead single-nucleotide polymorphism (SNPs) for replication follow-up was set to p<5×10^−6^ to allow loci with suggestive association to be included at the replication stage. GWAS 2 (sensitivity GWAS analysis): included sex, age and the top 15 PCs as covariates; the top 15 independent loci were follow-up at Stage 2.

#### Loci discovery and annotation

Independent genomic risk loci and lead variants (for p<5×10^−6^) were derived from FUMA (V.1.3.1)^26^ based on GWAS summary statistics, as previously described.^27^ For a locus to be defined as independent it had to be separated from other loci by at least 500 kb of genomic distance; the top-ranking SNPs were deemed potential lead markers.

#### Power analysis

The expected power to identify a true association between a SNP and HCC development in ArC was calculated using the GAS Power Calculator.^28^ The power for SNPs with minor allele frequencies of >20% was estimated to be 49% for alleles with a relative risk of 1.5, increasing to 81% for a relative risk of 1.6, for a p value threshold of 5×10^−8^ (online supplemental table 1).

#### Replication analysis

In stage 2, the selected SNPs were validated in independent samples from the UK, Germany and Italy. Study-specific β estimates and standard errors were further analysed using fixed-effect meta-analysis. Two criteria were required to demonstrate replication: (1) p<5.55×10^−3^ (corresponding to p<0.05 after Bonferroni correction for nine tests in the primary analysis); or p<3.33×10^−3^ (corresponding to p<0.05 after Bonferroni correction for 15 tests in the sensitivity analysis) (2) and consistency of allelic effect direction between discovery and replication samples (online supplemental methods F).

#### Additional replication analyses

The association between novel risk loci and HCC/liver cancer were also assessed using: (1) publicly available summary statistics from a recent alcohol-related HCC GWAS performed by Trépo *et al* 14; (2) data from two large population-based cohorts (Finngen and BioBank Japan) and (3) data from a UK cohort of patients with HCV-related cirrhosis (STOP-HCV) (online supplemental methods F).

#### Association with other cancers (pleiotropy)

Moreover, we assessed if novel risk loci were associated with selected cancers unrelated to the liver in both the UKB and FinnGen population-based cohorts. Each cancer phenotype was defined by ICD codes present in hospital admissions, death records and cancer registry records. In addition, the NHGRI-EBI Catalogue of human GWAS was searched for association of novel risk loci with cancer phenotypes (online supplemental methods F).

#### Meta-analysis GWAS

A fixed-effect meta-analysis restricted to markers present in all data sets (n=5 552 382) was performed using METAL^29^ to: (1) use the total study sample (n=3080) for the discovery stage and (2) to determine the combined effect size of replicated loci across stages 1 and 2 datasets.

#### eQTL analysis

Variants at novel loci were tested for cis-eQTL effect on gene expression in: (1) liver tissue (n=266) using the database of the Genotype-Tissue Expression Project (GTEx) release V8^30^ and (2) whole-blood (n=24 376) using the database of the eQTLGen Consortium.^31^

#### SNP Heritability Analysis

The proportion of phenotypic variance explained by the additive genetic effect of common genome-wide significant SNPs (*h*²_SNP_: SNP heritability) was estimated using a Genomic relatedness matrix REstricted Maximum Likelihood analysis implemented in GCTA^32^ (online supplemental methods G).

### Association with HCC-related phenotypes

Replicating loci were tested in the total UKB for association with two HCC-related phenotypes: leucocyte telomere length^33^ and liver fat content.^34^ Leucocyte telomere length was available for 474 074 participants in UKB (Field ID: 22191), while liver fat content was available for 8315 imaging substudy participants (Field ID: 22436) (online supplemental methods H).

### Patient and public involvement

There was no patient and public involvement in the design and conduct of this study.

## Results

### GWAS and validation of the loci

After imputation a total of 7 946 762 variants with a MAF >0.01 were tested for association with HCC in 1066 cases with ArC and HCC and 844 controls with ArC but with no evidence of HCC (table 1).

**Figure 1:**
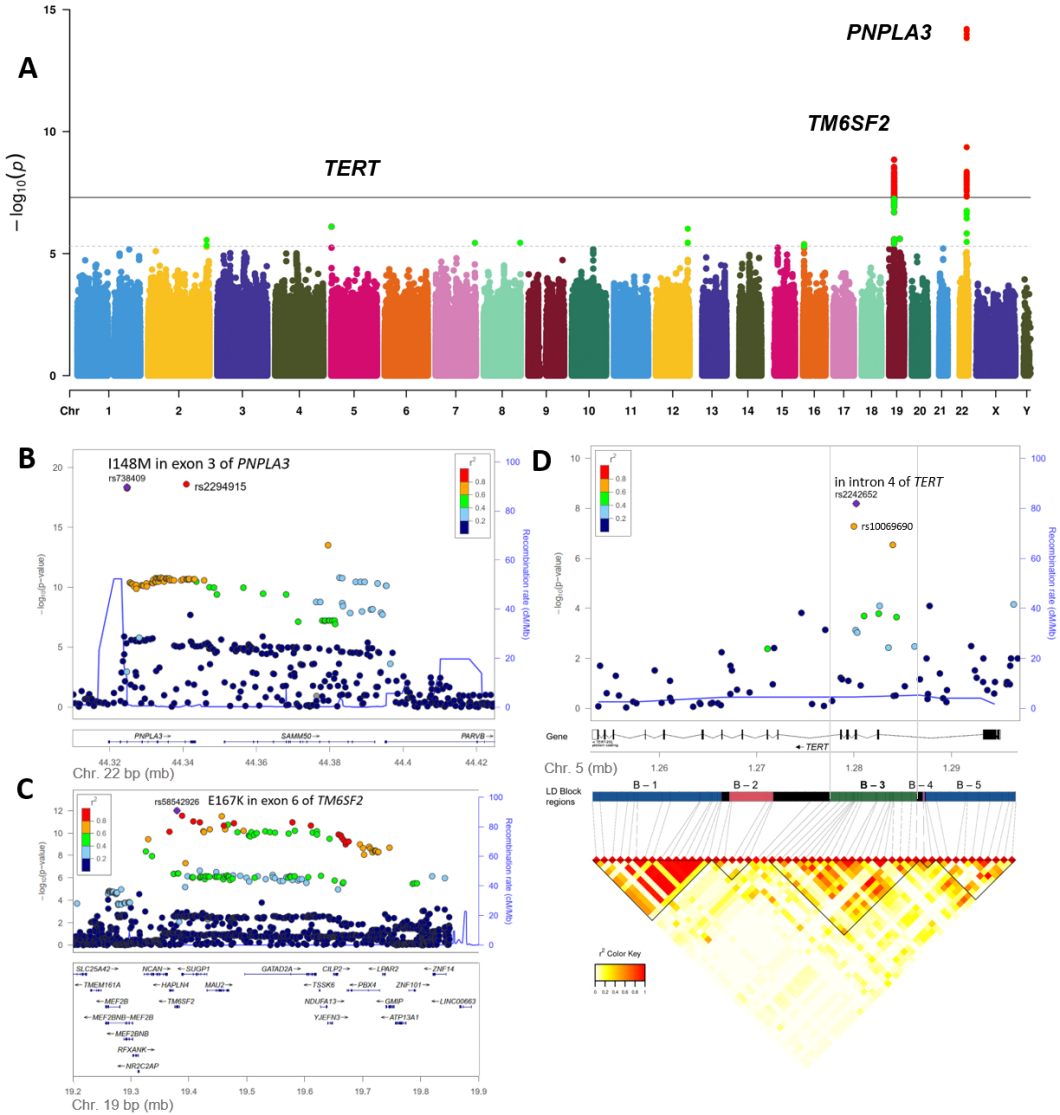
Genome-wide association study (Discovery GWAS) results. Principal findings of genetic analyses. (A): Manhattan plot of genome-wide association results for alcohol-related hepatocellular carcinoma (HCC) in the primary discovery cohort. P values (−log10) are shown for SNPs that passed quality control. The genome-wide significance threshold (5×10^−8^) is shown as a black line. The threshold for replication follow-up (p<5×10^−6^) is shown as a dashed line. Gene names for replicating loci (table 2) are shown. Variants with significance p<5×10^−8^ are highlighted in red, those with p<5×10^−6^ are highlighted in green. (B) Locus plot for HCC risk locus *PNPLA3*. The −log10 (p values, meta-analysis of discovery and replication samples) are plotted against SNP genomic position based on NCBI Build 37, with the names and location of nearest genes shown at the bottom. The variant with the lowest p value (lead variant) in the discovery analysis in the region is marked by a purple diamond. SNPs are coloured to reflect correlation with the most significant SNP, with red denoting the highest LD (r2 >0.8) with the lead SNP. The top association signal is located in exon 3 of *PNPLA3*. Estimated recombination rates from the 1000 Genomes Project (hg19, EUR population) are plotted in blue to reflect the local LD structure. (C) Locus plot for HCC risk locus *TM6SF2*. The top association signal is located in exon 6 of *TM6SF2*. (D) Locus plot for HCC risk locus *TERT*. Fine-mapping analysis of the *TERT* association signals. Annotated LD-Blocks are clusters of strong pairwise LD SNPs and reflect the LD pattern in the Discovery GWAS cohort. The lead association signal is located in intron 4 of the *TERT* gene (annotated on the reverse strand), located in LD block B-3 spanning from intron 4 to intron 2 of *TERT*. NCBI, National Center for Biotechnology Information; SNP, single-nucleotide polymorphism.

Associations with HCC were observed at genome-wide significance (p<5×10^−8^) for two independent genomic loci viz *PNPLA3* and *TM6SF2* (table 2; figure 1A, online supplemental figure 1). The strongest signal was at rs2294915, located in *PNPLA3* (p=6.21×10^−15^) which encodes 1-acylglycerol-3-phosphate O-acyltransferase. This tag SNP rs2294915, located in intron 8 of *PNPLA3*, is in strong linkage disequilibrium (LD) (r^2^=0.92) with the functional variant rs738409 C>G p.I148M in exon 3 of *PNPLA3* that yielded a similar *p* value at the discovery stage (p=7.23×10^−15^, OR (95% CI)=1.71 (1.49 to 1.96)).

**Table 2:** Association results for lead markers of regions entering the validation stage of the primary and sensitivity GWAS analysis

Lead SNPs	Locus	Chr	SNP ID	EA, ED	Discovery (stage 1)			Replication (stage 2)		Eff. Dir.	I^2^	*p*_heterog._	Combined (stage 1 & 2)‡		
					P value*†	OR (95% CI)	EAF Ca|Co|Eur	Meta P value^+^*^†^	OR (95% CI)				Meta P value^+^*^†^	OR(95% CI)	I^2^
GWAS analysis 1 (pc adjusted)*															
SNP 1	*PNPLA3§*	22	rs2294915	T+	**6.21×10^−15^**	1.71 (1.50 to 1.96)	.49|.36|.24	**6.19×10^-6^**	1.89 (1.44 to 2.50)	+++	0	0.517	**2.44×10^−19^**	1.75 (1.55 to 1.97)	0
	*PNPLA3*	22	rs738409	G+	**7.23×10^−15^**	1.71 (1.49 to 1.96)	.48|.35|.22	**9.74×10^-6^**	1.89 (1.42 to 2.50)	+++	0	0.578	**4.31×10^−19^**	1.74 (1.54 to 1.97)	0
SNP 2	*TM6SF2¶*	19	rs58489806	T+	**1.42×10^−9^**	1.87 (1.53 to 2.29)	.17|.10|.08	**5.22×10^-4^**	1.91 (1.33 to 2.76)	++−	54	0.110	**3.04×10^−12^**	1.88 (1.57 to 2.25)	32
	*TM6SF2*	19	rs58542926	T+	**2.81×10^−9^**	1.94 (1.56 to 2.42)	.15|.08|.07	**7.58×10^-5^**	2.11 (1.46 to 3.04)	++−	61	0.076	**1.00×10^−12^**	1.98 (1.64 to 2.40)	43
SNP 3	*TERT*	5	rs2242652	A-	7.87×10^−7^	0.64 (0.53 to 0.76)	.13|.19|.19	**1.07×10^-3^**	0.48 (0.31 to 0.74)	−−−	0	0.814	**6.40×10^−9^**	0.61 (0.52 to 0.72)	0
SNP 4	*LINC00939*	12	rs12371263	A-	9.59×10^−7^	0.63 (0.52 to 0.76)	.16|.21|.20	0.332	0.83 (0.57 to 1.21)	−+−	0	0.535	–	–	–
SNP 5	*DMAC2*	19	rs17318596	A-	2.49×10^−6^	0.71 (0.61 to 0.82)	.33|.40|.37	0.849	1.03 (0.77 to 1.38)	−+−	15	0.308	–	–	–
SNP 6	*SP100*	2	rs6743289	C-	2.77×10^−6^	0.72 (0.62 to 0.82)	.45|.52|.47	0.046	0.75 (0.57 to 1.00)	−−−	0	0.936	–	–	–
SNP 7	*GPIHBP1*	8	rs118088203	T-	3.60×10^−6^	0.24 (0.13 to 0.44)	.01|.03|.02	0.229	1.64 (0.73 to 3.07)	+++	0	0.697	–	–	–
SNP 8	*CNPY1*	7	rs12698003	T+	3.65×10^−6^	1.39 (1.21 to 1.60)	.46|.39|.41	0.053	0.74 (0.55 to 1.00)	−−−	41	0.179	–	–	–
SNP 9	*GLYR1*	16	rs741692	T+	4.16×10^−6^	1.58 (1.30 to 1.92)	.18|.12|.15	0.783	1.05 (0.73 to 1.51)	++−	0	0.541	–	–	–
GWAS analysis 2 (pc, sex, age adjusted)†															
SNP 1	*PNPLA3§*	22	rs2294915	T+	**6.31×10^−14^**	1.76 (1.52 to 2.05)	.49|.36|.24	**3.24×10^-5^**	1.89 (1.40 to 2.54)	+++	0	0.428	**1.06×10^-17^**	1.79 (1.57 to 2.04)	0
	*PNPLA3*	22	rs738409	G+	**1.67×10^−13^**	1.75 (1.51 to 2.03)	.48|.35|.22	**4.17×10^-5^**	1.85 (1.37 to 2.50)	+++	0	0.448	**5.35×10^-17^**	1.77 (1.55 to 2.02)	0
SNP 2	*TM6SF2¶*	19	rs143988316	T+	**4.40×10^−8^**	1.91 (1.51 to 2.41)	.16|.09|.07	5.17×10^-2^	1.54 (1.00 to 2.38)	++−	0	0.621	**9.14×10^-9^**	1.81 (1.51 to 2.16)	0
	*TM6SF2*	19	rs58542926	T+	1.21×10^−7^	1.93 (1.51 to 2.45)	.15|.08|.07	**1.56×10^-4^**	2.16 (1.45 to 3.22)	++−	48	0.149	**8.80×10^-11^**	1.99 (1.61 to 2.44)	26
SNP 3	*SCN5A*	3	rs6599222	C+	2.86×10^−6^	1.53 (1.28 to 1.84)	.25|.20|.21	0.977	1.01 (0.68 to 1.48)	−++	0	0.984	–	–	–
SNP 4	*intergenic*	13	rs148892410	A-	3.77×10^−6^	0.16 (0.07 to 0.35)	.01|.02|.01	0.798	1.45 (0.09 to 24.2)	++−	17	0.299	–	–	–
SNP 5	*intergenic*	2	rs6739777	G-	5.03×10^−6^	0.69 (0.59 to 0.81)	.29|.34|.30	0.388	0.86 (0.61 to 1.21)	−+−	40	0.193	–	–	–
SNP 6	*ENSG00000269151*	19	rs143660337	A-	5.14×10^−6^	0.41 (0.28 to 0.60)	.03|.05|.04	0.151	1.68 (0.83 to 3.41)	++−	0	0.589	–	–	–
SNP 7	*LOC105374308*	3	rs58339845	T-	5.84×10^−6^	0.46 (0.33 to 0.65)	.05|.07|.07	0.361	1.34 (0.72 to 2.50)	+++	0	0.919	–	–	–
SNP 8	*intergenic*	7	rs16869539	G+	5.96×10^−6^	1.48 (1.25 to 1.75)	.36|.30|.37	0.537	0.90 (0.65 to 1.25)	−−−	0	0.983	–	–	–
SNP 9	*CELF2*	10	rs2277212	T+	6.84×10^−6^	1.57 (1.29 to 1.91)	.75|.70|.74	0.282	1.22 (0.85 to 1.74)	+−+	16	0.303	–	–	–
SNP 10	*intergenic*	7	rs6462611	C+	7.82×10^−6^	1.41 (1.21 to 1.64)	.49|.44|.50	0.017	0.68 (0.49 to 0.93)	++−	0	0.465	–	–	–
SNP 11	*ENSG00000227757*	21	rs2017196	T+	8.73×10^−6^	1.70 (1.34 to 2.14)	.89|.85|.88	0.092	0.68 (0.44 to 1.06)	−−−	0	0.513	–	–	–
SNP 12	*TERT*	5	rs2242652	A-	9.28×10^−6^	0.64 (0.52 to 0.78)	.13|.19|.19	**2.60×10^-4^**	0.41 (0.25 to 0.66)	++−	0	0.699	**4.08×10^-8^**	0.60 (0.50 to 0.72)	17
SNP 13	*RARB*	3	rs7617311	A+	9.32×10^−6^	1.50 (1.25 to 1.80)	.28|.21|.26	0.454	0.87 (0.61 to 1.25)	−+−	0	0.751	–	–	–
SNP 14	*CIAO2A*	15	rs2922508	T+	9.53×10^−6^	1.61 (1.30 to 1.99)	.17|.13|.15	0.153	1.33 (0.90 to 1.97)	+++	0	0.797	–	–	–
SNP 15	*intergenic*	2	rs56209271	T-	9.67×10^−6^	0.69 (0.59 to 0.82)	.28|.34|.30	0.168	0.78 (0.55 to 1.11)	−−−	61	0.075	–	–	–

*OR and p value adjusted for top 15 PCs of genetic ancestry.

†OR and p value adjusted for sex, age and top 15 PCs of genetic ancestry.

‡The results of the combined analyses are only provided for variants meeting a Bonferroni corrected p<0.05 at the replication stage (printed in bold face).

§The tag SNP rs2294915 in PNPLA3 is in LD (r2= 0.92) with the functional variant rs738409 previously reported at the PNPLA3 locus^58 59^.

¶The intergenic tag SNP rs143988316 is in strong LD (r2= 0.88) with the functional variant rs58542926 previously reported at the TM6SF2 locus.^60^

+, Significance derived from a fixed effect meta-analysis; Ca, Cases (Cirrhosis with HCC); Chr, chromosome; Co, Controls (Cirrhosis without HCC); EA, effect allele; EAF, allele frequency of the effect allele; ED, effect direction; HCC, hepatocellular carcinoma; I^2^, percentage of between cohort heterogeneity; LD, linkage disequilibrium; p_heterog_, heterogeneity p value of the meta-analysis; SNP, single-nucleotide polymorphism.

The other signal associated with HCC at genome-wide significance was rs58489806, located in intron 1 of *MAU2* (p=1.49×10^−9^) encoding MAU2 sister chromatid cohesion factor; 49 additional genome-wide significant SNPs were mapped to this locus. The variant rs58489806 is in strong LD (r^2^=0.80) with the coding variant rs58542926 p.E167K at the *TM6SF2* locus (encoding transmembrane 6 superfamily member 2) that yielded (p=2.81×10^−9^, OR (95% CI)=1.94 (1.56 to 2.42)) at the discovery stage.

In stage 2, the nine lead SNPs from HCC associated loci were validated in independent cohorts from the UK, Germany and Italy in fixed-effect meta-analysis (table 1; online supplemental tables 2–4). In addition to rs2294915 in *PNPLA3* (p=6.19×10^−6^) and rs58489806 in *TM6SF2/MAU2* (p=5.22×10^−4^), disease association was replicated for the minor allele in rs2242652:A (p=1.07×10^−3^) in *TERT* (telomerase reverse transcriptase) (table 2). In the combined analysis of all stage 1 and stage 2 samples, the association of rs2242652:A in *TERT* with alcohol-related HCC attained genome-wide significance (p=6.41×10^−9^, OR (95% CI)=0.61 (0.52 to 0.72) (table 2). The protective effect associated with carriage of *TERT* rs2242652:A remained significant after correction for sex, age, body mass index (BMI), type 2 diabetes and the top 15 PCs of genetic ancestry, but did not reach genome-wide significance (p=7.94×10^−5^; OR (95% CI)=0.63 (0.50 to 0.79) (online supplemental table 5) reflecting the loss of power associated with the high number of missing BMI and diabetes data points in the analysis (table 1).

A sensitivity analysis in which the genome-wide analysis was additionally adjusted for sex and age also showed genome-wide significant association with HCC for two independent genomic loci *PNPLA3* and *TM6SF2* with HCC and suggestive evidence of association for *TERT* (p=9.28×10^−6^). (table 2; online supplemental figures 2 and 3). Of the top 15 associated loci, only the variants in *PNPLA3*, *TM6SF2* and *TERT* were replicated (table 2).

The combined GWAS meta-analyses of stage 1 and 2 data sets of the primary and the sensitivity analyses confirmed genome-wide significant association with HCC for genomic loci in rs738409 in *PNPLA3*, rs58542926 in *TM6SF2* and rs2242652 in *TERT*. No additional risk locus attained genome-wide significance p<5.0×10^−8^ (online supplemental table 6). Forest plots showing the association between genomic loci in *PNPLA3, TM6SF2, TERT* and HCC are shown in online supplemental figures 4–6. Regional association plots of these three loci are shown in figure 1B–1D and in online supplemental figures 7–9.

Previously reported associations of HCC in the context of ArC with variants of *HSD17B13* rs72613567:TA (p=8.95×10^−3^; OR=0.81 (95% CI 0.69 to 0.95) and *APOE* rs429358:C (p=5.44×10^−3^; OR=0.74 (95% CI 0.60 to 0.91) were nominally significant in this study, but did not achieve genome-wide significance in the discovery cohort (online supplemental tables 5 and 7). In contrast, a recently reported association between rs708113:T near *WNT3A* was not confirmed (online supplemental tables 5 and 7). Other previously described HCC risk loci, for example, *DEPDC5* in HCV-related HCC^35^ or *STAT4* and *HLA-DQ* 36 were not significantly associated with ArC-related HCC in this study (online supplemental table 7).

**Figure 2:**
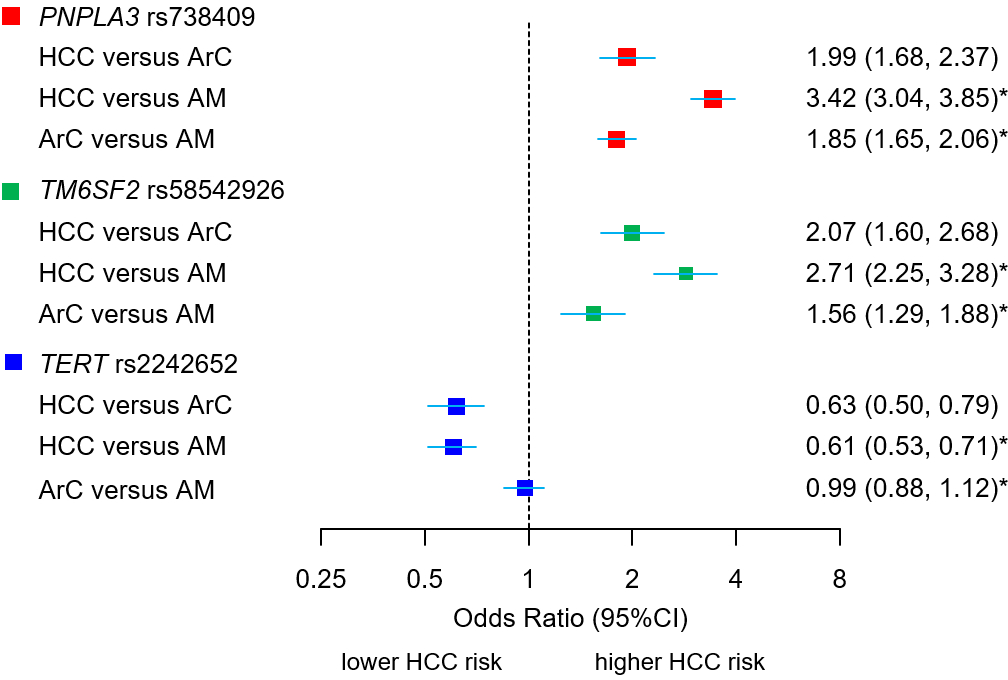
Association between novel (*TERT*) and confirmed loci (*PNPLA3*, *TM6SF2*) with HCC and cirrhosis phenotypes. ORs and 95% CIs for the susceptibility loci for alcohol-related HCC and alcohol-related cirrhosis (ArC) in comparison to alcohol misusers without cirrhosis (AM). The comparison HCC vs ArC displays allelic ORs of combined stage 1 and 2 samples (meta-analysis), derived from allele dosage data, adjusted for age, sex, BMI, type 2 diabetes status and top 15 principal components of genetic ancestry. *The comparison HCC versus AM and ArC versus AM display unadjusted allelic ORs derived from 2×2 contingency tables of allele counts observed in the total cohort, provided in online supplemental tables 2–4. BMI, body mass index; HCC, hepatocellular carcinoma.

Allelic and genotypic associations for *TERT* were highly significant, in the univariate analyses, for the comparisons HCC vs ArC (P_allelic_=2.81×10^−11^, P_genotypic_ 2.32×10^−10^) and HCC versus alcohol misuse but not for ArC vs alcohol misuse using combined genotype counts from the stage 1 and 2 data sets (online supplemental table 8; figure 2). The protective effect for HCC was greater in homozygous carriers of *TERT* rs2242652:A (OR=0.41 (95% CI 0.25 to 0.67)) than in heterozygous carriers (OR=0.61 (95% CI 0.51 to 0.72)). In contrast, variants in *PNPLA3* and *TM6SF2* were strongly associated both with ArC and ArC-related HCC (online supplemental tables 9 and 10, figure 2).

### Fine-mapping of *TERT* locus

In the primary meta-analysis of stage 1 and stage 2 samples, the strongest association signal was obtained for the minor allele in rs2242652:A (p=6.40×10^−09^; OR=0.61 (95% CI 0.52 to 0.72)), although the alternative allele in rs10069690:T was similarly associated (p=5.19×10^−08^, OR=0.66 (95% CI 0.57 to 0.77)). Both variants are located in intron 4 of *TERT* and are correlated (r^2^=0.70; online supplemental table 11). The analysis of LD structure at the *TERT* locus showed that the association signal spans a narrow range from intron 2 to intron 6 of *TERT*—here termed LD block B-3 region (online supplemental table 11 and figure 7). The conditional analysis on allele dosage of rs2242652:A or rs10069690:T on each of the 20 SNPs from the B-3 region confirmed rs2242652 to be the lead locus (online supplemental table 11 and 12). Indeed, none of the other variants within the B-3 block, including rs10069690 was associated with HCC after conditioning on rs2242652 (online supplemental table 11).

### Replication of the *TERT* variant’s association with HCC

Significant associations were observed between rs2242652:A and HCC in patients with HCV-related cirrhosis (p=0.047; OR=0.72 (95% CI 0.53 to 0.99) and in the population-based FinnGen, UKB and BioBank Japan cohorts (table 3, online supplemental figure 10 and table 13).

**Table 3:** Replication of *TERT* variants in patients with alcohol-related and chronic HCV-related cirrhosis and in population-based cohorts

Cohort	Controls	Cases phenotype (ICD-10)	N cases | controls	*TERT* Variant	EA	P value	OR (95% CI)
Current study*	ALD cirrhosis*	C22.0 Liver cell carcinoma (HCC) in alcohol-related cirrhosis	1214 | 1866	rs2242652	A	6.40×10^−9^	0.61 (0.52 to 0.72)
Replication cohorts							
Trépo *et al†*	ALD F0-F4 fibrosis	C22.0 HCC in alcohol-related liver disease (F0-F4 fibrosis)	775 | 1332	rs2242652	A	0.179	0.89 (0.75 to 1.06
Stop-HCV‡	HCV cirrhosis	C22.0 Liver cell carcinoma (HCC) in HCV-related cirrhosis	169 | 890	rs2242652	A	0.047 *§*	0.72 (0.53 to 0.99)
Zhang *et al*¶	Healthy volunteers	C22.0 Liver cell carcinoma (HCC)	473 | 564	rs2242652	A	0.004	0.70 (0.55 to 0.90)
Dong et al**	Healthy volunteers	C22.0 Liver cell carcinoma (HCC) (hepatitis-induced)	162 | 106	rs10069690	T	0.00014*§*	0.36 (0.21 to 0.63)
FinnGen††	General population	C22 malignant neoplasm of liver and intrahepatic bile duct	442 | 204 070	rs2242652	A	0.007	0.80 (0.68 to 0.94)
UKBB‡‡	General population	C22 malignant neoplasm of liver and intrahepatic bile duct	874 | 348 465	rs2242652	A	0.027	0.87 (0.78 to 0.97)
UKBB‡‡	General population	C22.0 Liver cell carcinoma (HCC)	383 | 348 956	rs2242652	A	0.028	0.80 (0.66 to 0.98)
BBJ Japan§§	BBJ population¶¶	C22.0 Liver cell carcinoma (HCC)	1866 | 195 745	rs72709458	T	0.00031	0.84 (0.76 to 0.92)

*Combined effect estimates of stage 1 and 2 samples of current study as shown in table 1 (for comparison).

†Cases: patients with ALD (80% with F3-4 fibrosis; 20% F0-2 fibrosis) and HCC, controls: patients with ALD (90% with F3-4 fibrosis, 10% F0-2 fibrosis) from Trépo *et al*.^14^

‡Cases: patients with HCV related cirrhosis and HCC, controls: patients with HCV related cirrhosis without HCC (online supplemental methods F).

§Allelic ORs were calculated from 2×2 tables on allele counts. Significance was calculated as 1 df χ^2^ test.

¶Zhang *et al*,^52^ Huang *et al* 51 Han Chinese patients with HCC (individuals were excluded from the study if they had HCV).

**Dong *et al* 61 62 male Han Chinese patients with viral hepatitis-induced primary hepatocellular carcinoma (r2=0.85 between rs10069690:T and rs2242652:A, both variants are in high linkage disequilibrium).

††General population controls (excluding all cancers).

‡‡As UKB data were incorporated into our discovery analysis, further interrogation of liver cancer phenotypes from UKB does not constitute independent validation.

§§Variants rs2242652 and rs10069690 were not available in the summary GWAS data from Ishigaki *et al* 63 (PMID: 32514122, publicly available from http://jenger.riken.jp/en/result) (rs72709458 is the closest proxy to rs2242652 (r2=0.973)).

¶¶Removed diseases from control samples (biliary tract cancer, oesophageal cancer, gastric cancer, colorectal cancer and pancreatic cancer).

ALD, alcohol liver disease; BBJ, BioBank Japan; EA, effect allele; FinnGen, FinnGen Biobank; GWAS, Genome-Wide Association Studies; HCV, hepatitis C virus; ICD, International Classification of Diseases;; UKB, UK Biobank.

### Association of *TERT* variants with non-liver cancers

Associations between *TERT* rs2242652:A and the 10 most frequent cancers were explored in the UKB and FinnGen (FG) cohorts (online supplemental figure 10). Significant associations were observed with bladder cancer (FG: p=6.10 × 10^-6^, OR=0.83 (95% CI 0.67 to 0.90)), UKB: p=5.82 × 10^−7^, OR=0.84 (95% CI 0.79 to 0.90)), and prostate cancer (FG: p=5.11 × 10^−11^, OR=0.87 (95% CI 0.84 to 0.90)), UKB: p=6.16 × 10^−16^; OR=0.86 (95% CI 0.83 to 0.89)) while weaker associations were observed for lung and skin cancer. The effect sizes for prostate and bladder cancer were smaller than those for HCC/primary liver cancer in these cohort (UKB: HCC: p=0.028; OR=0.80 (95% CI 0.66 to 0.89), FG: primary liver cancer: p=0.009; OR=0.81 (95% CI 0.69 to 0.95)). These effect sizes are broadly consistent with those reported in the NHGRI-EBI Catalogue of human GWASs (online supplemental table 14).

### Additive effect of risk variants

The proportions of patients with ArC, in the discovery and validation cohorts, who developed HCC increased with cumulative carriage of the risk increasing alleles rs738409:G in *PNPLA3*, rs58542926:T in *TM6SF2* and rs2242652:G in *TERT* (online supplemental figure 11). In the discovery cohort, the OR for alcohol-related HCC was 2.12 (95% CI 1.76 to 2.56) in patients carrying three to four risk alleles, and 5.24 (95% CI 2.82 to 9.77) in patients carrying five to six risk alleles (online supplemental table 15). In the UK replication cohort, the ORs for carriage of three to four risk alleles and five to six risk alleles were even higher at 3.25 (95% CI 1.84 to 5.73) and 17.8 (95% CI 6.38 to 49.6), respectively (online supplemental figure 12 and table 15).

### Association with leucocyte telomere length and liver fat content in the UKB

The minor allele of the lead variant in *TERT* rs2242652:A (p=2.12×10^−44^) was significantly associated with an increase in LTL, as was rs10069690:T which is in strong LD with the lead variant (p=4.08×10^−84^) (online supplemental table 16). Additional variants located in the tested interval, that is, rs7726159, showed even stronger association with LTL (p=1.16×10^−219^) despite weak LD with rs2242652 (r^2^=0.354) (online supplemental table 11). The main association signals for HCC and LTL were both located in the LD block B-3 region, but a direct correlation in the strength of association was not observed (online supplemental figure 13 and table 11). Lead variants in *PNPLA3* and *TM6SF2* were not significantly associated with LTL—rs738409 (p=0.458) and rs58542926 (p=0.475), but showed significant associations with liver fat content *viz.* rs738409 (p=3.39×10^−61^), rs58542926 (p=5.94×10^−45^), respectively (online supplemental table 16); rs2242652 in *TERT* was not significantly associated with liver fat content (p=0.144).

### eQTL Analysis

Carriage of rs2242652:A was associated with increased expression of *TERT* in blood leucocytes (p=1.39×10^−5^) (online supplemental table 11). However, no significant eQTLs were found for rs2242652 in liver using the GTEx data base or in any other tissues.^30^

### SNP Heritability Analysis

The percentage heritability for ArC- HCC explained by additive genome-wide SNPs expressed as *h* ^2^ was 29.6% on the observed scale (GWAS cohort) and either 20.4% or 25.7% on the liability scale assuming a disease prevalence of 1% or 2.5%, respectively (online supplemental table 20 20). The proportion of phenotypic variation due to the underlying genetic variation in the *PNPLA3*/*TM6SF2*/*TERT* LD regions, expressed as *h* ^2^, was 7.5% on the observed scale and 4.2% or 5.3% on the liability scale, assuming the same disease prevalence (online supplemental table 17). The proportion of the total SNP heritability due to variance component 1 (*PNPLA3*/*TM6SF2*/*TERT* variants) was 25.5% for model 1, adjusted for 15 PCs, and 22.2% for model 2 adjusted for sex, age and 15 PCs. After adjustment of variance component 1 for lead variants rs738409 in *PNPLA3*/rs58542926 in *TM6SF2*/rs2242652 in *TERT h2* was reduced to 0.000001%, indicating that the genetic risk of variance component 1 was fully captured by the three identified lead variants.

## Discussion

In this study, associations at genome-wide significance were identified between HCC in ArC and previously recognised variants in *PNPLA3* and *TM6SF2,* and with a variant in *TERT* (telomerase reverse transcriptase) on chromosome 5 not previously associated with this phenotype. In combination, these three loci may explain up to 25% of the total SNP heritability in HCC in patients with ArC.

The identification of host genetic risk factors for alcohol-related HCC has been largely undertaken using a candidate gene approach. Candidate genes have invariably been selected because of their association with progression of alcohol-related liver injury and positive robust associations for variants rs738409 in *PNPLA3,* and rs58542926 in *TM6SF2* have been identified.^8 9^ These variants are known to modify liver fat content and signalling, but how they influence the mechanisms leading to tumour initiation or promotion is largely unknown.^37 38^ In this study, the increased risk associations between HCC in ArC and rs738409 in *PNPLA3* and rs58542926 in *TM6SF2* were confirmed, at genome-wide significance.

Significant associations have also been identified between rs72613567 in *HSD17B13* and rs429358 in *APOE* and a *reduced* risk for developing HCC in ArC.^9–11^ In this study, these protective associations were confirmed but failed to reach a detectable genome-wide significance level (online supplemental table 5).

Further insights into the genetic landscape of HCC in the context of ArLD were recently provided by Trépo *et al* 14 who undertook a discovery GWAS of HCC in people with a spectrum of ArLD in a French-Belgian collaborative effort. Similar to this study, they confirmed genome-wide significant associations with an increased risk for developing alcohol-related HCC and variants in *PNPLA3 and TM6SF2.* In addition, they found an equally significant association with rs708113 in the *WNT3A-WNT9A* region on chromosome 1q42, which was associated with a reduced risk for development of alcohol-related HCC. The presence of this variant was associated with increased immune cell infiltration of tumour tissues and a lower frequency of beta-catenin mutations (*CTNNB1*) which frequently precede HCC occurrence.^39^ This protective effect of rs708113 was not observed in people with HCC on a background of chronic HCV infection or NAFLD.^14^

In this study, rs708113 in the *WNT3A-WNT9A* region was not significantly associated with the development of HCC, possibly reflecting differences in the cohort composition between the two studies although both comprised of participants of European descent. This assumption of population diversity is supported, to some extent, by the fact that in the French-Belgian cohorts the effect size of rs58542926 in *TM6SF2* surpassed that of rs738409 in *PNPLA3* which has been the strongest single genetic risk locus for ArLD in previous candidate gene association studies.^40^

The key finding in this study was the identification of a risk locus in *TERT*, that is not related to lipid turnover, inflammation or fibrogenesis but appears to be highly influential in HCC development.^41^ Like any cancer, HCC arises when healthy hepatocytes acquire mutations in specific genes regulating cell division. In HCC, *TERT* is the most commonly mutated gene, with mutations (mainly in the promoter region) present in up to 60% of tumours.^42^ This lends clear plausibility to the association reported in this study between inherited polymorphisms in *TERT* and alcohol-related HCC. Similar relationships between germline and somatic variants have been identified for other cancers types.^43^ The biology of telomere regulation is still being unravelled and remains incompletely understood. *TERT* encodes the catalytic subunit (hTERT) of the enzyme telomerase, which maintains telomeres, the repeated DNA segments found at the ends of chromosomes. In most cells telomeres progressively shorten as the cells repeatedly divide and this eventually triggers the cell to stop dividing or to undergo apoptosis. Telomerase counteracts the shortening of telomeres by adding small repetitive DNA segments to the ends of the chromosomes during each cell division cycle.^44^ Telomerase is also abnormally active in most cancer cells.^45^ *TERT* expression levels significantly affect telomerase activity in various cells and tissues.^46^ Previous studies show that older age, male gender and cirrhosis (all classic risk factors for HCC) are associated with shorter telomere length in liver tissue.^47^ Thus, this study, showing that rs2242652:A reduces HCC risk while at the same time increasing telomere length, is directionally concordant with this previous work. From a mechanistic perspective, it could be that shorter telomeres leave cells more vulnerable to mutations in driver genes, thus accelerating hepatocarcinogenesis.^47^ It is important to point out however that the association between rs2242652 and HCC may not be entirely mediated through telomere length alone. Indeed, for variants in *TERT*, we found that there was not a good correlation between strength of association with telomere length and strength of association with HCC. Thus, rs2242652 is not simply acting as a surrogate for telomere length. Relevant to this point is that, as part of its non-canonical functions *TERT* also regulates the WNT/β-catenin pathway.^48 49^ This signalling pathway is suggested to play a role in alcohol-induced fibrogenesis and hepatocarcinogenesis, too.^14 50^ However, regarding the risk of alcohol-induced fibrosis/cirrhosis, our data unequivocally show no association with rs2242652 in *TERT*.

There is also some support for the findings in this study in previous publications. In the GWAS undertaken by Trépo *et al* 14 rs2242652:A in *TERT* was associated with a reduced risk of HCC, but the OR was weaker than in this study and did not reach statistical significance (p=0.179; OR=0.89 (95% CI 0.75 to 1.06)). However, carriage of rs10069690:T in *TERT*—the nearest available proxy to rs2242652—was associated with a significantly reduced risk of HCC development (p=0.036; OR=0.84 (95% CI 0.71 to 0.99)). The significant association between rs2242652:A in *TERT* with liver and intrahepatic bile duct carcinoma in the population-based FinnGen cohort and with HCC in the BioBank Japan cohort additionally substantiate this study’s findings. A case–control study in Han Chinese involving 473 patients with HCC and 564 healthy volunteers, which is reported in two separate publications (Huang *et al* and Zhang *et al* 51 52), also identified associations between variants in *TERT* and the development of HCC; carriage of rs2242652:A in *TERT* was associated with a reduced risk for HCC development (OR =0.70, 95% CI 0.55 to 0.90, p =0.004), as was carriage of rs10069690:T (OR =0.75, 95% CI 0.59 to 0.96, p=0.021). Patients with chronic HCV infection were excluded from this study but otherwise it is unclear whether the patients with HCC had underlying chronic liver disease and, if so, its aetiology.

A number of HCC risk loci have been identified in patients developing HCC on a background of chronic HCV^35^ and chronic HBV,^36^ but none was significantly associated with ArC-related HCC in this study. However, there is some evidence that variants in *TERT* may predispose to HCC in other types of chronic liver disease. Thus, a significant association between rs2242652:A and the reduced risk for developing HCC was observed in patients with HCV-related cirrhosis in this study following reanalysis of the STOP-HCV^53^ data. Also, in a small study Dong *et al* 54 showed that carriage of the common allele T in rs10069690 is associated with an increased risk of developing HCC on a background of chronic viral hepatitis (OR = 2.78, 95% CI 1.62 to 4.78, p=0.00014). Thus, the association between rs2242652 and HCC may extend beyond its relationship with ArC. Further work is warranted to assess if a similar association applies to patients with NAFLD. A previous study showed that rare loss of function germline mutations in *TERT* are enriched in patients with NAFLD-HCC relative to controls—however, the specific relevance of the rs2242652 locus in this patient group is unknown.^55^

*TERT* rs2242652 has also been implicated in the susceptibility for developing other cancers but the direction of association seems to vary between cancer types (online supplemental table 14). In this study, rs2242652:A was significantly associated with reduced risks for developing bladder cancer and prostate cancer in the UKB and FinnGen cohorts. Kote-Jarai *et al* 56 found that carriage of *TERT* rs2242652:A: was associated with a lower risk for developing prostate cancer and with increased *TERT* expression which has been reported to improves survival, in prostate cancer. Further large studies involving diverse populations are clearly needed.

This study has a number of strengths including: (1) use of a two stage GWAS approach; (2) large, carefully selected case and control samples focusing on HCC in patients with established ArC; (3) careful exclusion of confounding comorbidities; (4) uniform inclusion of Caucasians participants of European ancestry; (5) the protective effect of rs2242652:A on HCC has been confirmed in the Japanese and Chinese population, suggesting that it may be applicable to East Asian population too, and (6) although the study was confined, by design, to patients with HCC on a background of ArC a cohort of patients with HCV-related HCC was also included to assess the generalisability of our findings to other aetiologies. The study also has a number of limitations: (1) it was performed retrospectively and hence potentially important information such as the lifetime alcohol history, information on diabetes and obesity were not generally available; (2) it had comparatively low power to detect true disease associations with smaller effect sizes (OR <1.4), at the levels of significance needed for GWAS analysis, and (3) only a minority of the HCC cases had histological confirmation of the diagnosis so tissue specimen for molecular analyses were not available.

In conclusion, this study identifies *TERT* rs2242652:A as a novel genetic factor for HCC development in ArC and confirmed the importance of the *PNPLA3* and *TM6SF2* as risk factors for HCC in this population. While the association between HCC and rs2242652:A in *TERT* is robust, the functional implications of carriage of this protective allele remains unclear. Carriage of rs2242652:A was significantly associated with an increase in leucocyte telomere lengths, but data on its effect on *TERT* transcription in liver tissue were not available. Thus, the functional implications of this association require further study in this specific context since the impact of *TERT* transcription, telomere length and the risk of malignancy remains controversial.^57^

## Data Availability

Data are available on reasonable request.
