# Supplementary material for: Genetic variation in TERT modifies the risk of hepatocellular carcinoma in alcohol-related cirrhosis: results from a genome-wide case-control study
Source: Gut. 2022 Jul 4;72(2):381–91. doi: 10.1136/gutjnl-2022-327196 (PMC9872243; doi:10.1136/gutjnl-2022-327196)
Supplement: Supplementary data [file gutjnl-2022-327196supp001.pdf]

Supplementary information

SUPPLEMENTARY METHODS A

Patient cohorts

Patients with alcohol-related cirrhosis referred to university centres in Germany (Dresden, Bonn, Rostock, Heidelberg, Munich, Leipzig, Halle, Homburg, Hannover, Hamburg, Kiel, Magdeburg, Frankfurt), Switzerland (Berne, Zürich, Lausanne), Austria (Salzburg/Oberndorf, Graz, Vienna), United Kingdom (London and UK Biobank), and Italy (Milan), were included and assigned to respective groups, as detailed in Table 1.

Ethics

The study protocol was approved by the ethics committees of the participating institutions; all included subjects provided written informed consent prior to inclusion into the study.

Germany / Switzerland / Austria Alcohol Cohort (Discovery cohort)

The diagnosis of alcohol-related cirrhosis was established as previously described [1]. In a substantial fraction of subjects included, transient elastography was performed to include patients with a liver stiffness measurement (Fibroscan, Echosens, Paris) of above 19 kPa (IQR<20%) indicating cirrhosis as per current consensus [2]. Morbidly obese (body mass index ≥45kg/m^2) patients were excluded. The diagnosis of HCC was established as previously described [3], [4] and based on histological examination of tumour tissue or typical evidence on magnetic resonance imaging, using liver-specific contrast enhancement of focal lesions that were hypervascular in the arterial phase with a fast wash-out in the portal venous or delayed phases, and revealed a serum-alpha-fetoprotein level of >200ng/ml [5], [6].

SUPPLEMENTARY METHODS B

ArC nested case-control dataset:

We created a nested case control study for ArC HCC using data from the UKB resource as described in the main text.

Participants were excluded from the case-control study for any of the following reasons

- non-White British ancestry (inferred via UKB field ID:22006);
- poor quality genetic sample (defined by UKB field ID: 22027);

- Second or first-degree relations to another participant in the case-control dataset (inferred via a kinship coefficient ≥0.121).

Individual-level data for approximately 6.2 million genetic variants were available in the version 3 UKB imputed genetic data sets, after exclusion of variants with (a) minor allele frequency <1%, (b) gross deviation from the Hardy-Weinberg equilibrium ($P < 1.0 \times 10^{-10}$), (c) imputation information score <0.8, (d) high level of missing data (>10%), and (e) non-biallelic or duplicate variants.

Evidence for alcohol-related cirrhosis was determined as:

A hospital admission for alcohol cirrhosis of liver.

- K70.3 (Alcoholic cirrhosis of liver)

We also excluded Individuals with other liver disease aetiologies (viral hepatitis, hemochromatosis and autoimmune liver disease).

Viral hepatitis and autoimmune liver disease were defined as the presence of one or more of the following ICD codes in any diagnostic position within an in-patient hospital admission record

- K754 (Autoimmune hepatitis)
- K743 (primary biliary cirrhosis)
- K744 (secondary biliary cirrhosis)
- K745 (biliary cirrhosis, unspecified)
- B16 (acute hepatitis B)
- B17 (other acute viral hepatitis)
- B18 (Chronic viral hepatitis)
- B19 (unspecified viral hepatitis)

Hemochromatosis was defined as homozygous carriage of the C282Y variant (rs18005762), as determined from the version 3 UKB genetic dataset (downloaded May 2019).

These nested case-control data were then pooled with additional patients recruited from the Centre for Hepatology at the Royal Free Hospital, London (N=306). These patients were similarly of self-reported English, Scottish, Welsh or Irish descent, and had a history of prolonged alcohol misuse as described previously [1]. All were examined by two experienced, senior clinicians for signs of liver injury. The diagnosis of HCC was based on histological examination of tumour tissue. Histological examination was undertaken, whenever possible, of liver biopsy material obtained by

percutaneous, ultrasound-guided or transjugular routes or else of explant or postmortem liver tissue. Blood was screened for antibodies to hepatitis B, hepatitis C, mitochondrial, nuclear, smooth muscle, liver and kidney autoantibodies; iron, total iron-binging capacity and ferritin; copper and caeruloplasmin; α1 antitrypsin and tissue transglutaminase. Patients were excluded if they had any other potential cause of liver injury such as chronic viral hepatitis or autoimmune liver disease; genetic haemochromatosis; Wilson's disease; alpha-1 antitrypsin deficiency or celiac disease. Patients with ArC (with no evidence of HCC) were diagnosed on the basis of a history of alcohol dependence and histological examination of liver tissue or on the basis of compatible historical, clinical, laboratory, radiological and endoscopic features.

University of Bonn (Germany) Alcohol Cohort (Replication cohort 2)

The replication cohorts included 238 Caucasian patients with ArC (42 with HCC) from the Hepatology/Gastroenterology department of the University Bonn, as detailed previously [7]**.**

University of Milan (Italy) Alcohol Cohort (Replication cohort 3)

The replication cohorts included 72 Caucasian patients with ArC (36 with HCC) from the department of Pathophysiology and Transplantation of the State University of Milan.

SUPPLEMENTARY METHODS C

Validation cohorts

For post-hoc risk assessment additional patients with alcohol misuse, but without cirrhosis were included. Non-cirrhotic control patients (n=1080) with alcohol misuse but no evidence of significant liver injury were recruited at psychiatry centers specialized in addiction medicine in Regensburg, Munich and Mannheim (all in Germany) as described, in detail, previously [1]. In brief, patients had a background of alcohol consumption of at least 60 g/d for women and 80 g/d for men for ≥10 years, all patients received a diagnosis of alcohol dependence (according to DSM-IV criteria). None had historical, clinical or laboratory evidence of liver disease, and its absence was confirmed either by a liver stiffness measurement (Fibroscan, Echosens, Paris) of below 6 kPa (IQR<20%) or by the absence of histological liver damage. These patients were then pooled together with additional non-cirrhotic patients with alcohol abuse (n=99) recruited from the Salem Medical Center, Heidelberg as described, in detail, previously [8].

Additional, non-cirrhosis control patients (n=341) recruited from the Centre for Hepatology at the Royal Free Hospital, London were diagnosed on the basis of a history of alcohol dependence and the absence of liver injury on histology or the absence of historical, clinical, radiological or

endoscopic features suggestive of significant liver injury either at presentation of during prolonged follow-up. All patients underwent abdominal ultrasound and/or abdominal computed tomography/magnetic resonance imaging, as indicated. All underwent routine upper gastrointestinal endoscopy.

SUPPLEMENTARY METHODS D

DNA preparation and genotyping

Discovery cohort

Genomic DNA was extracted from peripheral blood samples according to standard procedures, quantified using the PicoGreen dsDNA Assay kit (Invitrogen) and normalized to a concentration of 50 ng/µl. Genotyping on Illumina BeadChip arrays was performed according to the manufacturer's instructions, as described before [1].

For the present study, >665,000 SNPs (662,835 with assigned rs-numbers) were genotyped in a clinical case-control panel of 1910 patients with ArC from Germany and Switzerland with a total genotyping rate of 0.998. The discovery GWAS included 1,066 patients with ArC and HCC and 844 patients with ArC with no evidence of HCC from Germany and Switzerland (Table 1) genotyped on the Infinium®Global Screening Array (GSA) (version 24v2, Illumina). Samples were called using GenomeStudio (v2.0, Illumina), exported to Plink file format and imputed as described below.

Genotype data quality control

In all data sets, individuals with genotyping success <97%, outlying autosomal heterozygosity (more than 3 SD from the mean) or a kinship coefficient (pˆ) <0.185 and those failing gender checks were excluded from analysis. Multidimensional scaling (MDS) analysis was performed on a cleaned, LD-pruned data set (indep-pairwise; excluding the human leukocyte antigen (HLA) region at chr. 6: 28,477,797–33,448,354; minor allele frequency >0.01, Hardy-Weinberg equilibrium $P > 1\times10^{-6}$, genotyping rate threshold for each marker >98% and genotyping rate threshold for each individual >97%) that was merged with HapMap Phase 3 data from 11 different populations. Individuals deviating by more than 3 SD from the median European MDS cluster were excluded as population outliers. All quality control filtering was performed using PLINK (v1.9).

MDS plot of discovery cohort (non-European ancestry outlier removal)

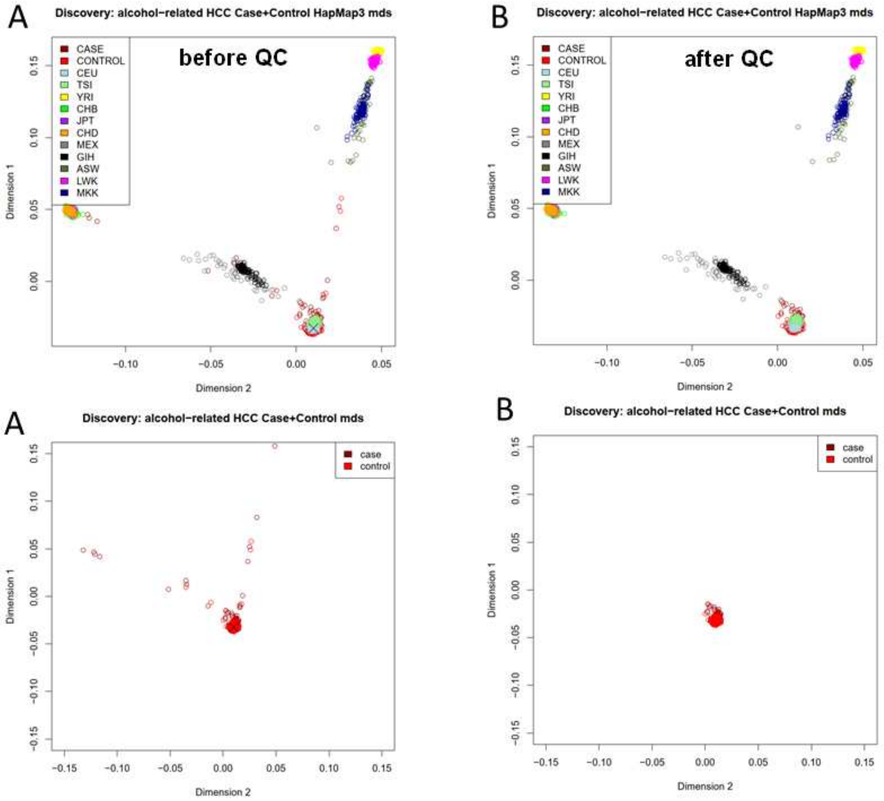

MDS plot of United Kingdom replication cohort (non-European ancestry outlier removal)

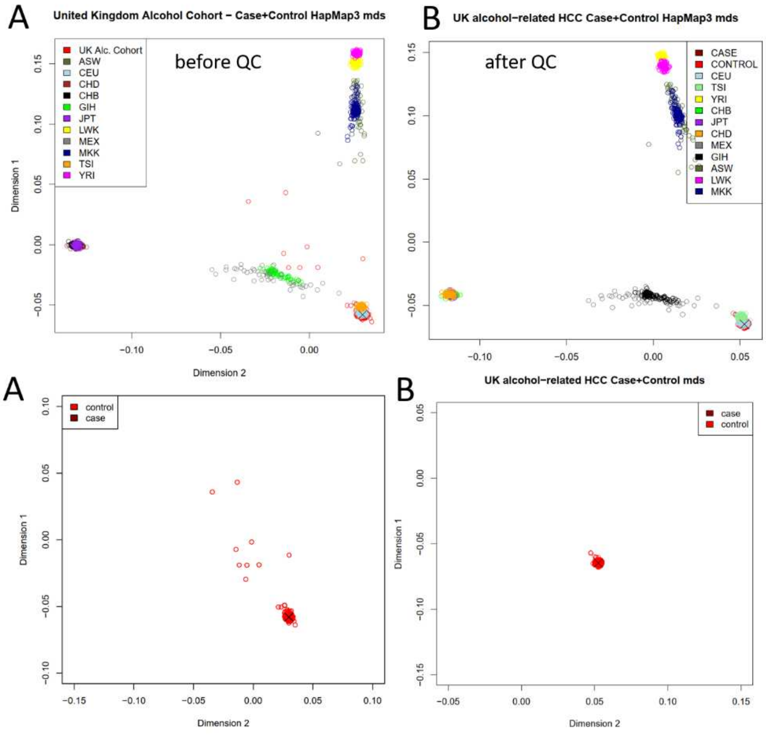

Replication cohorts

Germany:

Replication samples from Germany (cohort 2) were genotyped on the OmniExpress array (version 24v1-0a), exported to Plink format and imputed as described below.

Italy:

Replication samples from Italy (cohort 3) were genotyped on the Infinium®Global Screening Array (GSA) (version 24v2, Illumina), exported to Plink format and imputed as described below.

United Kingdom:

In total, 652 patients with a history of prolonged alcohol misuse (306 patients with alcohol-related cirrhosis and 346 non-cirrhotic controls from the UK) were genotyped using the OmniExpress array (version 24v1-0a, Illumina) as described previously [1]. Samples were called using GenomeStudio (v2.0, Illumina). Genotype data was then imputed to HRC reference panel as described below. For replication of lead variants, imputed VCF format probability data was exported to Plink format and merged with Plink format genotype data from 606 patients from the United Kingdom (UK) Biobank Resource based on data from the version 3 release. Imputed genotype data from 606 patients genotyped on the UK BiLEVE and the UK Biobank Axiom array were obtained from the UK Biobank Resource [9] (under application number 8764).

The merged data set was subjected to quality control procedures described below. Association tests were performed on the merged data set on discrete genotypes.

The UK Biobank analysis was based on data from the version 3 release of the UKB imputed genetic data set. Participants were excluded from the case-control study for any of the following: non-White British ancestry (inferred via UKB field ID:22006); poor quality genetic sample (defined by UKB field ID: 22027); Second or first-degree relations to another participant in the case-control dataset (inferred via a kinship coefficient ≥0.121). Individual-level data for approximately 6.2 million genetic variants were available in the version 3 UKB imputed genetic data sets, after exclusion of variants with (a) minor allele frequency <1%, (b) gross deviation from the Hardy-Weinberg equilibrium ($P < 1.0\times10^{-10}$), (c) imputation information score <0.8, (d) high level of missing data (>10%), and (e) non-biallelic or duplicate variants.

Validation samples

Control subjects drinking excessively but without evident alcohol-related liver disease (AM) were recruited at psychiatry centers specialized in addiction medicine in Regensburg, Munich and Mannheim (all in Germany). All patients received a diagnosis of alcohol dependence (according to DSM-IV criteria). These patients were genotyped on the HumanHap550 (n = 407), Human610Quad (n = 329) and Human660w-Quad (n = 383) Illumina BeadChip arrays. Individuals with genotyping success <97% were excluded from further analysis, as described in detail before [10]. To harmonize the German data sets, genotype probabilities were generated from signal intensity data for each array. Sample phasing and genotype imputation were performed using IMPUTE2 to the1000 Genomes Project Phase 3 reference (October 2014 release). Expected genotypes were calculated from the genotype posterior probabilities using the software SNPTEST (v2.5.6; snptest (ox.ac.uk) [11].

Additional 99 AM control subjects were recruited from the Salem Medical Center, Heidelberg and were genotyped on the Infinium®Global Screening Array (GSA) (version 24v2, Illumina), exported to Plink format and imputed as described below.

SUPPLEMENTARY METHODS E

Imputation and GWAS meta-analysis

Genotype imputation was performed with Minimac4 to the HRC r1.1 (hg19) reference panel using the Michigan Imputation Server[12], [13]. In total 7,946,762 variants with imputation r^2>0.3, MAF>0.01 and HWE ($P>1x10^{-6}$) were tested for association with HCC using linear regression on allele dosages adjusted for top 15 principal components of genetic ancestry. Quality filtered plink files from the discovery and replication cohorts were prepared for imputation to the Haplotype Reference Consortium reference panel (HRC.r1-1.GRCh37) using the "HRC-1000G-check-bim" tool (Version 4.3.0) [14]. Phasing and genotype imputation was performed using the Michigan Imputation Server [15]. After imputation 7,778,317 variants were available for the discovery GWAS, after exclusion of variants with a minor allele frequency <0.01, deviation from Hardy-Weinberg equilibrium $P < 1\times10^{-6}$ and imputation information score <0.3.

SUPPLEMENTARY METHODS F

Replication analysis

In stage 2, the selected SNPs were validated in independent samples from the UK (n=860), Germany (n=238) and Italy (n=72). Study-specific β estimates and standard errors were derived from stage 2 samples and further analyzed using fixed-effect meta-analysis. Cochran's Q and I2 statistics were employed to assess consistency of effect and to quantify heterogeneity between sample sets. Two criteria were required to demonstrate replication: a) P value $< 5.55 \times 10^{-3}$ (corresponding to $P < 0.05$ after Bonferroni correction for 9 tests); and b) and consistency of allelic effect direction between discovery and replication samples. Four different multiplicative allelic models were analyzed: model (1) included the top 15 PCs, model (2) included sex, age and the top 15 PCs, model (3) included sex, age, BMI and the top 15 PCs and model (4) included age, sex, BMI, diabetes type 2 status and the top 15 PCs using Plink 2.0 for. Model 1 results were used in the primary replication analysis, model 2 results in the secondary replication analysis, model 3 and 4 estimates in *post-hoc* risk assessment (Suppl. Table 5).

Additional replication analysis of *TERT* variants in population-based cohorts

FinnGen Biobank

FinnGen is a public–private partnership project, combining genotyping data from Finnish biobanks with electronic health record data derived from national health registries. Genome-wide association study (GWAS) summary statistics for more than 1,800 phenotypes/endpoints, including for primary liver cancer, have been publicly released.

This study utilized the latest R6 data released (autumn 2020) pertaining to a sample size of 260,405 individuals. Cases were individuals with a history/diagnosis of primary liver cancer (ICD-10: C22 and ICD-9: 155), while controls were all individuals without a diagnosis of primary liver cancer (excluding all other cancers), largely comprised of individuals without any preexisting liver disease. GWAS summary statistics relating specifically to HCC were not available.

United Kingdom Biobank

As UKB data were incorporated into the discovery analysis, further interrogation of liver cancer phenotypes from UKB does not constitute independent validation. However, for reference purposes, we calculated the association between variants in *TERT* and liver-related cancer at the level of the entire UKB population.

Associations with three specific phenotypes were assessed.

1) HCC (ICD 10: C22.0).
2) Intrahepatic bile duct cancer (ICD 10: C22.1)
3) All liver related neoplasms (ICD10: C22)

N.B. the latter phenotype was selected in order to align with data from the FinnGen cohort.

As with previous analyses, UKB participants were restricted to those of white British ancestry (UKB field ID: 22006) and excluded those with a poor-quality genetic sample (defined by UKB field ID: 22027); and excluded related participants (inferred by a kinship coefficient ≥0.1).

Cases were defined as participants with the selected ICD code(s) in a hospital admission, death, or cancer registration record, either before or after UKB enrollment. The control group comprised UKB participants who did not meet the above definition of a case. All analyses were adjusted for sex, age at UKB enrolment, and the top 5 principal components of genetic ancestry.

BioBank Japan

BBJ is a prospective genome biobank that collaboratively collected DNA and serum samples from 12 medical institutions in Japan, managed by the Institute of Medical Science, the University of Tokyo. BBJ has recruited approximately 260,000 patients, mainly of Japanese ancestry. All study participants had been diagnosed with one or more of 47 target diseases. RIKEN Center for Integrative Medical Sciences contributed to genotyping of the BBJ samples.

Cases were participants with a history of hepatic cancer, defined by cancer registration with HCC (ICD-10: **C22.0**, or ICD-9: **155.0**). The control group included all BBJ participants without a history of HCC, of which most individuals would have had no history of chronic liver disease.

Additional replication analysis of *TERT* variants

In total, the STOP-HCV cirrhosis study comprises 1,059 patients with hepatitis C-related cirrhosis. Participants were recruited from 31 specialist liver clinics in the UK between January 2015 and July 2016[16]. Cirrhosis was defined through histologic assessment, imaging, or a validated serum biomarker consistent with liver cirrhosis (i.e., aspartate aminotransferase [AST]-to-platelet ratio index >2, FibroTest >0.73, or enhanced liver fibrosis score >10.48). Blood specimens collected at enrollment were used to generate host-genotyping data through the Affymetrix UK Biobank array. Imputation was performed in March 2022 using the Topmed imputation server. Missing hard called

genotypes were filled from dosage information to obtain the Euclidean-nearest best-guess genotypes.To generate prospective phenotype data, participants from England have been linked to national hospital admission, cancer registrations, and mortality data, in a similar way to the UKB.

The present analysis was restricted to participants from England to ensure complete data on hospital admissions, cancer registrations, and mortality. No restrictions were made for ethnicity/European ancestry. Participants with missing genotype data for the lead variant rs2242652 were also excluded. As with the UKB, cases were defined on the basis of an in-patient hospital admission, death, or cancer registration indicating HCC (ICD-10: C22.0; ICD-9: 155.0) either before or after study enrollment. Controls were all participants without a history of HCC. Allelic odds ratios were calculated from 2 × 2 tables on allele counts. Significance was calculated as one degree of freedom Chi-squared test.

Association with non-liver related cancers (pleiotropy analysis)

The association of *TERT* variant rs2242652 with non-liver related cancers were tested in the UKB and FinnGen population-based cohorts. Specifically, we quantified rs2242652's and rs10069690´s association individually with each of the 10 most frequent non-liver related cancers observed in the UKB population: C43 Malignant melanoma of skin; C44 Other malignant neoplasms of skin; C50 Malignant neoplasm of breast; C61 Malignant neoplasm of prostate; C18 Malignant neoplasm of colon; C20 Malignant neoplasm of rectum; C34 Malignant neoplasm of bronchus and lung; C67 Malignant neoplasm of bladder; C54 Malignant neoplasm of corpus uteri; C85 Other and unspecified types of non-Hodgkin's lymphoma. As before, each cancer phenotype was defined using data from: 1) hospital admissions; 2) mortality; and 3) cancer registries.

SUPPLEMENTARY METHODS G

SNP Heritability Analysis

The proportion of phenotypic variance explained by the additive genetic effect of common genome-wide significant SNPs (h^2_{SNP}: SNP heritability) was estimated by genome-based restricted maximum likelihood analysis using GCTA-GREML[17]. To obtain A SNP-based estimate of relatedness for each pair of individuals in the discovery GWAS cohort was obtained using imputed autosomal SNPs (N=7,585,576) with MAF > 1%, P_{HWE} >$1{\times}10^{-6}$ and imputation r2 > 0.3, were used to calculate the

genetic relationship matrix (GRM) between pairs of individuals. Missing hard called genotypes were filled from dosage information to obtain the Euclidean-nearest best-guess genotypes. The heritability (proportion of variance) explained by variants in the *PNPLA3* / *TM6SF2* / *TERT* LD regions that are associated with HCC in ArC with genome-wide significance were calculated using, a two genetic variance components model was established using a set of n=2026 variants from these regions as variance component 1 to allow the full genetic signal of these regions to be captured and the remaining >7.5 million GWAS variants (MAF >1%) as variance component 2, with sex, age and top 15 PCs as environment variance component. Obtained h^2 estimates were then transformed to the liabitly scale valid for binary traits assuming HCC frequencies of 1%-2.5% in ArC using the formula implemented in GCTA.

$$h2_{liability=} = h2_{observed} \frac{K(1-K)}{\varphi(\Phi_{-1}[K])_2} \frac{K(1-K)}{P(1-P)}$$

KK is the frequency of the binary trait in the population, PP is the frequency of the binary trait in the observed sample. The denominator of the first fraction is the squared probability density function evaluated at the KK quantile of the inverse cumulative density function of the standard normal distribution.

The proportion of the total SNP heritability due to variance component 1 (*PNPLA3* / *TM6SF2* / *TERT* variants) was calculated $\frac{h2(\mathrm{vc1})}{h2(\mathrm{vc0})+h2(\mathrm{vc1})}$ =%(h^2).

The three lead variants at the HCC risk loci (viz rs738409 in *PNPLA3*, rs58542926 in *TM6SF2* and rs2242652 in *TERT*) were included as covariates when estimating the additive genetic SNP heritability (h^2 SNP) in the joint analysis in order to validate the heritability explained by component 1.

SUPPLEMENTARY METHODS H

United Kingdom Biobank cohort:

Liver fat content and telomere length data

Leukocyte telomere length was available for 471,172 participants in UKB (Field ID: 22191), whilst liver imaging fat content data was available for 8315 imaging sub-study participants (Field ID: 22436). The analysis was based on data from the version 3 release of the UKB imputed genetic data set. Participants were excluded from these analyses for any of the following reasons: non-White British ancestry (inferred via UKB field ID:22006); poor quality genetic sample (defined by UKB field ID: 22027); Second or first-degree relations to another participant in the case-control dataset (inferred via a kinship coefficient ≥0.121).

Data Availability

All data generated or analyzed during this study are included in this published article (and its supplementary information files).

REFERENCES:

[1] S. Buch *et al.*, "A genome-wide association study confirms PNPLA3 and identifies TM6SF2 and MBOAT7 as risk loci for alcohol-related cirrhosis," *Nat Genet*, vol. 47, no. 12, pp. 1443–1448, Dec. 2015, doi: 10.1038/NG.3417.

[2] E. Nguyen-Khac *et al.*, "Non-invasive diagnosis of liver fibrosis in patients with alcohol-related liver disease by transient elastography: an individual patient data meta-analysis," *Lancet Gastroenterol Hepatol*, vol. 3, no. 9, pp. 614–625, Sep. 2018, doi: 10.1016/S2468-1253(18)30124-9.

[3] F. Stickel *et al.*, "Genetic variants in PNPLA3 and TM6SF2 predispose to the development of hepatocellular carcinoma in individuals with alcohol-related cirrhosis," *Am J Gastroenterol*, vol. 113, no. 10, pp. 1475–1483, Oct. 2018, doi: 10.1038/S41395-018-0041-8.

[4] F. Stickel *et al.*, "Genetic Variation in HSD17B13 Reduces the Risk of Developing Cirrhosis and Hepatocellular Carcinoma in Alcohol Misusers," *Hepatology*, vol. 72, no. 1, pp. 88–102, Jul. 2020, doi: 10.1002/HEP.30996.

[5] P. R. Galle *et al.*, "EASL Clinical Practice Guidelines: Management of hepatocellular carcinoma," *J Hepatol*, vol. 69, no. 1, pp. 182–236, Jul. 2018, doi: 10.1016/J.JHEP.2018.03.019.

[6] J. M. Llovet *et al.*, "EASL-EORTC clinical practice guidelines: management of hepatocellular carcinoma," *J Hepatol*, vol. 56, no. 4, pp. 908–943, Apr. 2012, doi: 10.1016/J.JHEP.2011.12.001.

[7] H. D. Nischalke *et al.*, "A common polymorphism in the NCAN gene is associated with hepatocellular carcinoma in alcoholic liver disease," *J Hepatol*, vol. 61, no. 5, pp. 1073–1079, 2014, doi: 10.1016/J.JHEP.2014.06.006.

[8] S. Mueller *et al.*, "Inflammation-adapted liver stiffness values for improved fibrosis staging in patients with hepatitis C virus and alcoholic liver disease," *Liver International*, vol. 35, no. 12, pp. 2514–2521, Dec. 2015, doi: 10.1111/LIV.12904/SUPPINFO.

[9] C. Bycroft *et al.*, "The UK Biobank resource with deep phenotyping and genomic data," *Nature 2018 562:7726*, vol. 562, no. 7726, pp. 203–209, Oct. 2018, doi: 10.1038/s41586-018-0579-z.

[10] S. Buch *et al.*, "A genome-wide association scan identifies the hepatic cholesterol transporter ABCG8 as a susceptibility factor for human gallstone disease," *Nature Genetics*, vol. 39, no. 8, pp. 995–999, Aug. 2007, doi: 10.1038/ng2101.

[11] J. Marchini and B. Howie, "Genotype imputation for genome-wide association studies," *Nature Reviews Genetics*, vol. 11, no. 7, pp. 499–511, 2010, doi: 10.1038/nrg2796.

[12] S. McCarthy *et al.*, "A reference panel of 64,976 haplotypes for genotype imputation," *Nat Genet*, vol. 48, no. 10, pp. 1279–1283, Oct. 2016, doi: 10.1038/NG.3643.

[13] S. Das *et al.*, “Next-generation genotype imputation service and methods,” *Nat Genet*, vol. 48, no. 10, p. 1284, Oct. 2016, doi: 10.1038/NG.3656.

[14] “McCarthy Tools.” https://www.well.ox.ac.uk/~wrayner/tools/ (accessed Nov. 09, 2021).

[15] “Home - Michigan Imputation Server.” https://imputationserver.readthedocs.io/en/latest/ (accessed Nov. 09, 2021).

[16] H. Innes *et al.*, “Comprehensive Comparative Analysis of Standard Validated, Genetic, and Novel Biomarkers to Enhance Prognostic Risk-Stratification in Patients With Hepatitis C Virus Cirrhosis,” *Clinical and Translational Gastroenterology*, vol. 13, no. 3, p. e00462, Mar. 2022, doi: 10.14309/CTG.0000000000000462.

[17] J. Yang, S. H. Lee, M. E. Goddard, and P. M. Visscher, “GCTA: a tool for genome-wide complex trait analysis,” *Am J Hum Genet*, vol. 88, no. 1, pp. 76–82, Jan. 2011, doi: 10.1016/J.AJHG.2010.11.011.

[18] E. Trépo *et al.*, “Common genetic variation in alcohol-related hepatocellular carcinoma: a case-control genome-wide association study,” *Lancet Oncol*, vol. 23, no. 1, pp. 161–171, Jan. 2022, doi: 10.1016/S1470-2045(21)00603-3.

[19] “hTERT single nucleotide polymorphism is associated with increased risks of hepatocellular carcinoma and tumor metastasis.” http://caod.oriprobe.com/articles/26058367/hTERT_single_nucleotide_polymorphism_is_associated_with_increased_risk.htm (accessed Apr. 28, 2022).

[20] “[hTERT single nucleotide polymorphism is associated with increased risks of hepatocellular carcinoma and tumor metastasis] - PubMed.” https://pubmed.ncbi.nlm.nih.gov/21269955/ (accessed Apr. 28, 2022).

SUPPLEMENTARY FIGURES

Supplementary Figure 1: QQ plot for Discovery GWAS 1 (pc adjusted)

Quantile-quantile (QQ) plot: A λ of 1.03 was obtained for the primary analysis. Y axis observed -log10P values, Y axis expected -log10P values.

Supplementary Figure 2: Manhattan Plot of Discovery GWAS 2 (sex, age and pc adjusted, secondary analysis)

Genome-wide association analysis of 1066 with ArC and HCC and 844 controls with ArC without HCC. GWAS analysis was adjusted for sex, age and top 15 principal components of genetic ancestry. P values are shown for SNPs that passed quality control. The genome-wide significance threshold ($P=5\times10^{-8}$) is shown as a solid line. The threshold for replication follow-up ($P=1\times10^{-5}$) is shown as a dashed line. The nearest gene is annotated for replicating loci. Variants with significance $P<5\times10^{-8}$ are highlighted in red, those with $P<1\times10^{-5}$ are highlighted in green.

Supplementary Figure 3: QQ plot of secondary GWAS 2: sex, age, PC adjusted

Quantile-quantile (QQ) plot: A λ of 1.02 was obtained for the secondary analysis. Y axis observed -log10P values, Y axis expected -log10P values.

Supplementary Figure 4: Forest plot of confirmed HCC risk locus *PNPLA3* for discovery and replication cohorts

Forrest plot showing the fixed-effects model (k=4) meta-analysis of allelic ORs of the *PNPLA3* rs738409:G allele, for regression model 1 adjusted for top principal components.

Supplementary Figure 5: Forest plot of confirmed HCC risk locus *TM6SF2* for discovery and replication cohorts

Forrest plot showing the fixed-effects model (k=4) meta-analysis of allelic ORs of the *TM6SF2* rs58542926:T allele, for regression model 1 adjusted for top principal components.

Supplementary Figure 6: Forest plot of the novel HCC associated locus *TERT* for discovery and replication cohorts

Forrest plot showing the fixed-effects model (k=4) meta-analysis of allelic ORs of the *TERT* rs2242652:A allele, for regression model 1 adjusted for top principal components.

Supplementary Figure 7: Regional plot *TERT* (Discovery and Replication cohorts combined)

Fine-mapping analysis of the *TERT* association signals. Annotated LD-Blocks are clusters of strong pairwise LD SNPs and reflect the LD pattern in the Discovery GWAS cohort. The lead association signal is located in intron 4 of the *TERT* gene (annotated on the reverse strand), located in LD block B-3 spanning from intron 4 to intron 2 of *TERT* (see Suppl. Table 12). The plot was generated using LocusZoom and R package ”gpart”.

Supplementary Figure 8: Regional plot *TM6SF2* (Discovery and Replication cohorts combined)

Fine-mapping analysis of the *TM6SF2* association signals. The −log10 (P values) are plotted against SNP genomic position based on NCBI Build 37. SNPs are colored to reflect correlation with the most significant SNP, with red denoting the highest LD (r2>0.8) with the lead SNP. Annotated LD-Blocks are clusters of strong pairwise LD SNPs and reflect the LD pattern in the Discovery GWAS cohort.

The lead association signal is located in exon 6 of the *TM6SF2* gene (annotated on the reverse strand). Estimated recombination rates from the 1000 Genomes Project (hg19/genomes March 2012 release, EUR population) are plotted in blue to reflect the local LD structure. Gene annotations were obtained from the UCSC Genome Browser.

Supplementary Figure 9: Regional plot *PNPLA3* (Discovery and Replication cohorts combined)

Fine-mapping analysis of the *PNPLA3* association signals. The −log10 (P values) are plotted against SNP genomic position based on NCBI Build 37. SNPs are colored to reflect correlation with the most significant SNP, with red denoting the highest LD (r2 >0.8) with the lead SNP. Annotated LD-Blocks are clusters of strong pairwise LD SNPs and reflect the LD pattern in the Discovery GWAS cohort. The lead association signal is located in exon 6 of the *PNPLA3* gene (annotated on the forward strand). Estimated recombination rates from the 1000 Genomes Project (hg19/genomes March 2012 release, EUR population) are plotted in blue to reflect the local LD structure. Gene annotations were obtained from the UCSC Genome Browser.

Supplementay Figure 10: Forest plots for the associations of *TERT* lead variant rs2242652 [A allele] with the 10 most common cancer in UK Biobank and FinnGen biobank populated-based cohorts

Forest plot showing association between lead variant rs2242652:A in *TERT* associated with HCC in ArC in the present study and the top 10 most frequent cancers observed in the UK Biobank and FinnGen cohort. In addition, the primary liver cancer phenotypes are highlighted in red. Associations are presented in terms of the LOR. An LOR of 0 indicates that the frequency of rs2242652:A is the same for cases as for controls. LORs were calculated using logistic regression under an additive genetic model.

Supplementay Figure 11: The relative proportion of patients with HCC grouped by the number of risk alleles in *PNPLA3*, *TM6SF2*, and *TERT*

Percentage of patient with HCC in the discovery and validation cohorts stratified by the sum of crude risk alleles of *PNPLA3* rs738409 ‘G’, *TM6SF2* rs58542926 ‘T’ and *TERT* rs2242652 ‘G’ carried by each patient, grouped into 3 categories.

Supplementay Figure 12: Association between the number of risk alleles in *PNPLA3*, *TM6SF2* and *TERT* and alcohol-related hepatocellular carcinoma

The association between the sum of crude risk alleles of *PNPLA3* rs738409 ‘G’, *TM6SF2* rs58542926 ‘T’ and *TERT* rs2242652 ‘G’ carried by each patient and alcohol-related hepatocellular carcinoma in the discovery and validation cohorts.

Supplementay Figure 13: HCC association results overlayed with leukocyte telomere length association results and regulation for gene *TERT*

GWAS association signals (-log10 P values) of the combined of discovery and replication samples plotted for the entire *TERT* gene in comparison to association results (-log10 P values) for leukocyte telomere length observed in the total European UK-Biobank population. Annotated LD-Blocks reflect the LD pattern in the discovery GWAS cohort. The lead association signal is located in intron 4 of the *TERT* gene (annotated on the reverse strand), located in LD block B-3 spanning from intron 4

to intron 2 of *TERT*. Strongest association signals for leukocyte telomere length are also located to LD block B-3. (for further details see Suppl. Table 12). The plot was generated using Ensembl.

Supplementary Figure 1: QQ plot for Discovery GWAS 1 (PC adjusted)

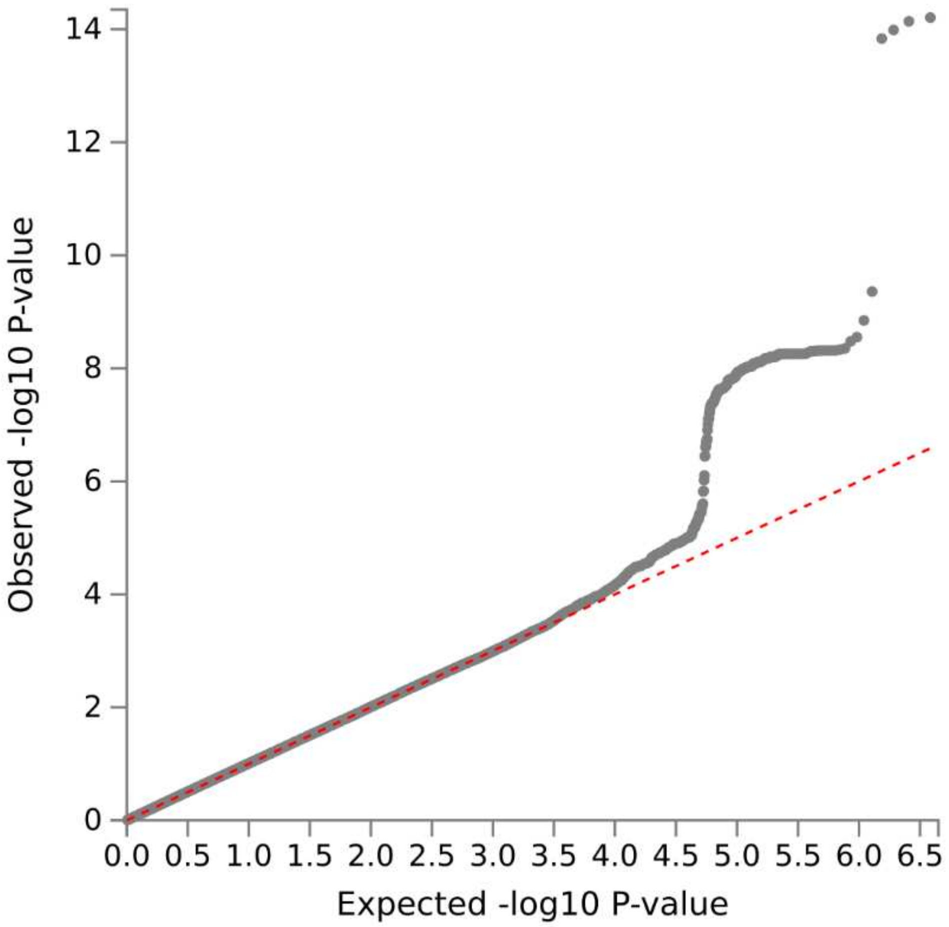

Supplementary Figure 2: Manhattan Plot of Discovery GWAS 2 (sex, age and PC adjusted)

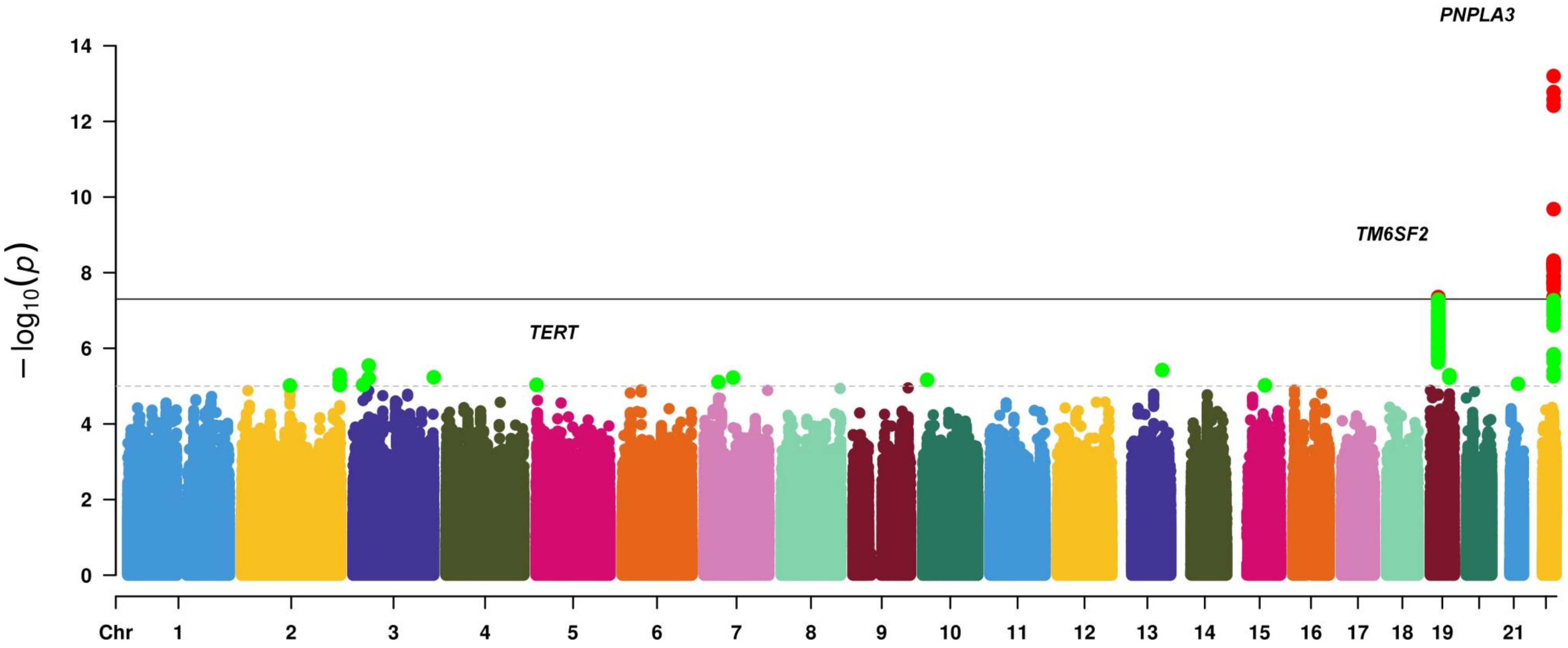

Supplementary Figure 3: QQ plot of secondary GWAS 2 (sex, age and PC adjusted)

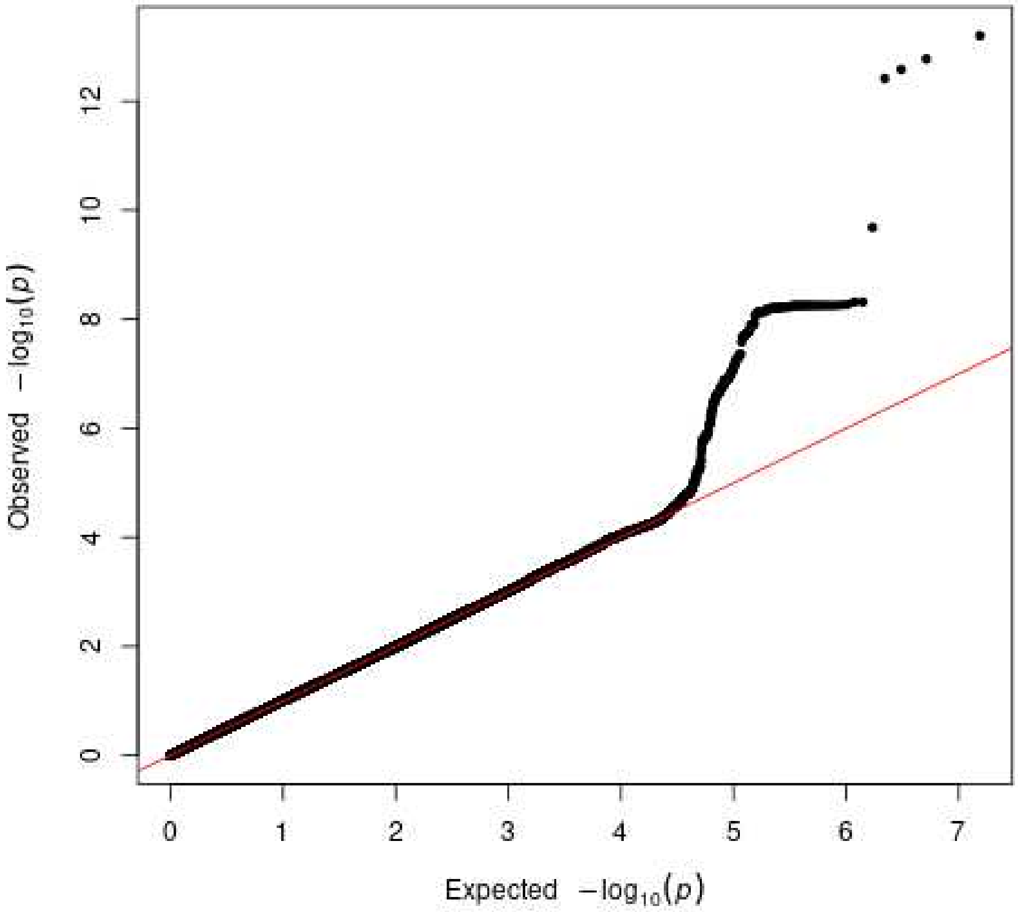

Supplementary Figure 4: Forest plot of confirmed HCC risk locus *PNPLA3* for discovery and replication cohorts

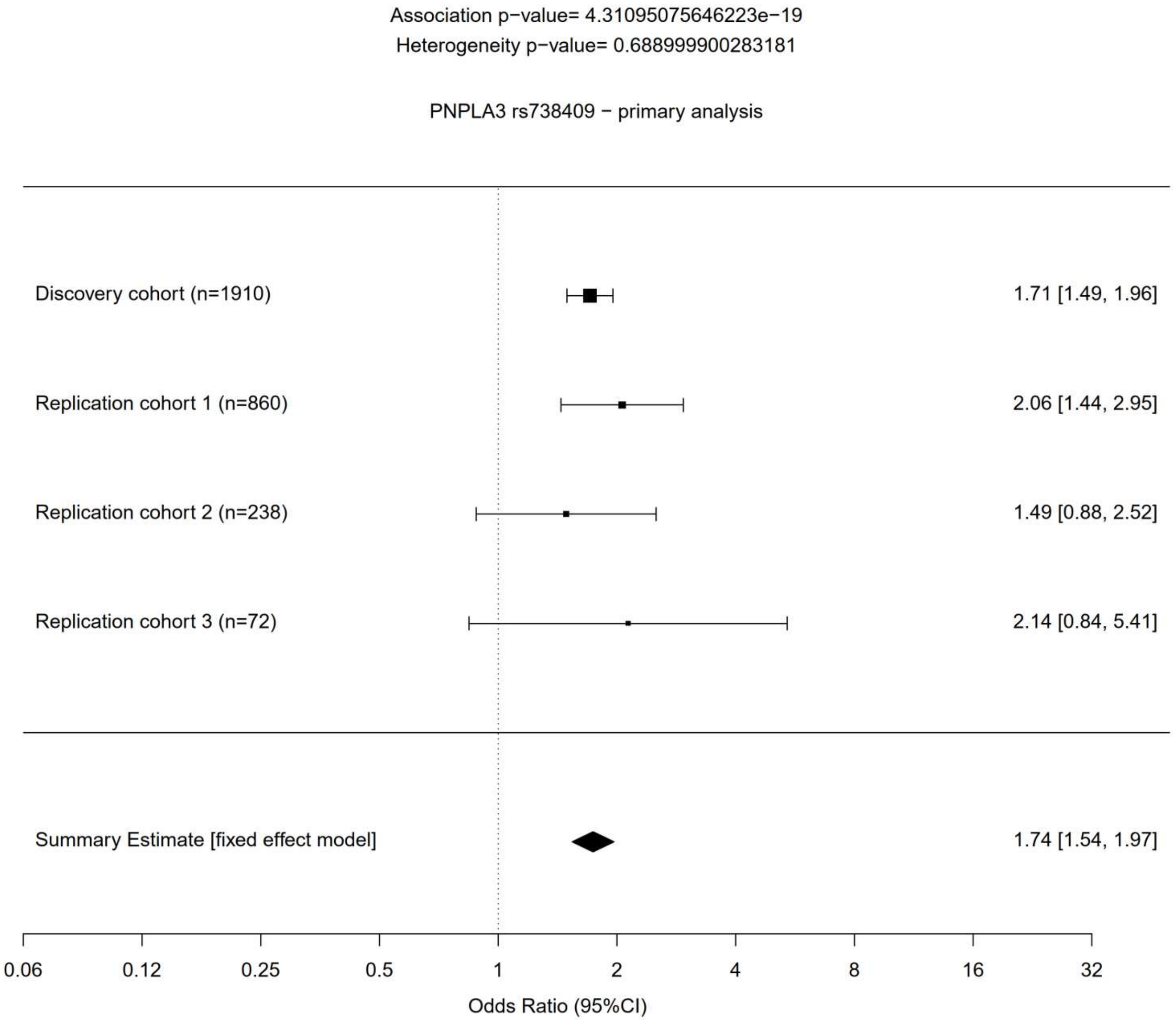

Supplementary Figure 5: Forest plot of confirmed HCC risk locus *TM6SF2* for discovery and replication cohorts

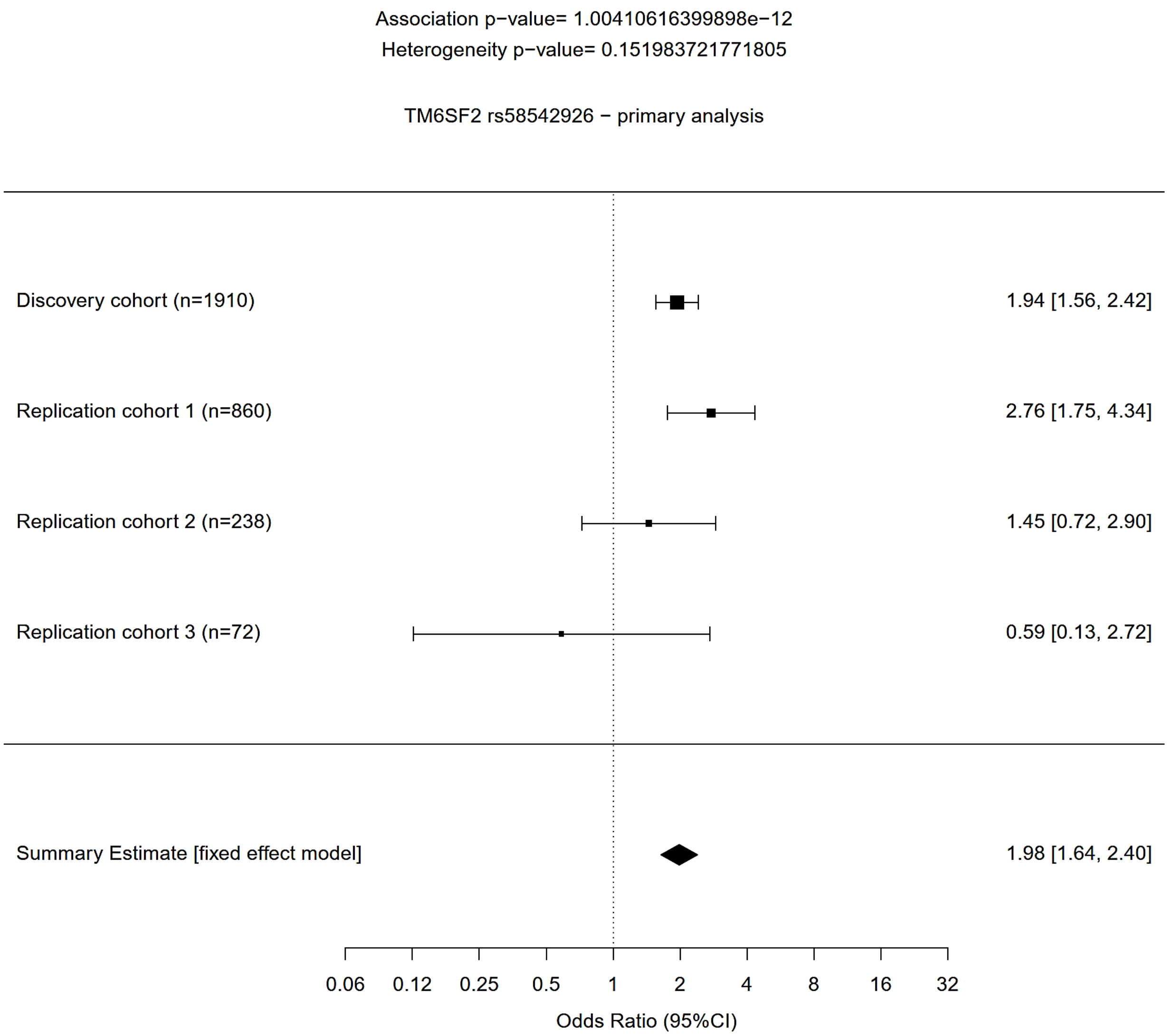

Supplementary Figure 6: Forest plot of the novel HCC associated locus rs2242652:A in *TERT* for discovery and replication cohorts

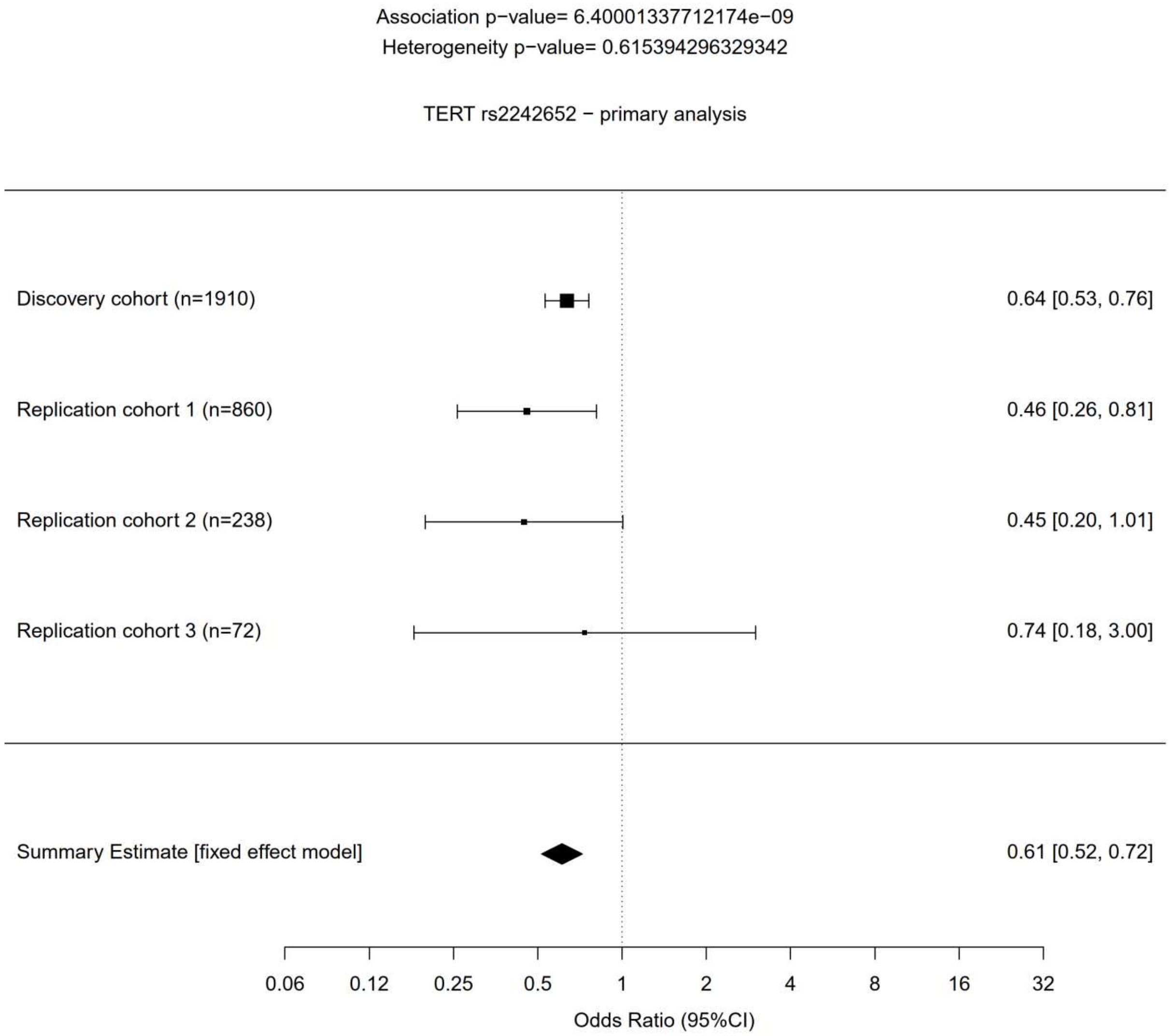

Supplementary Figure 7: Regional plot *TERT* (Discovery and Replication cohorts combined)

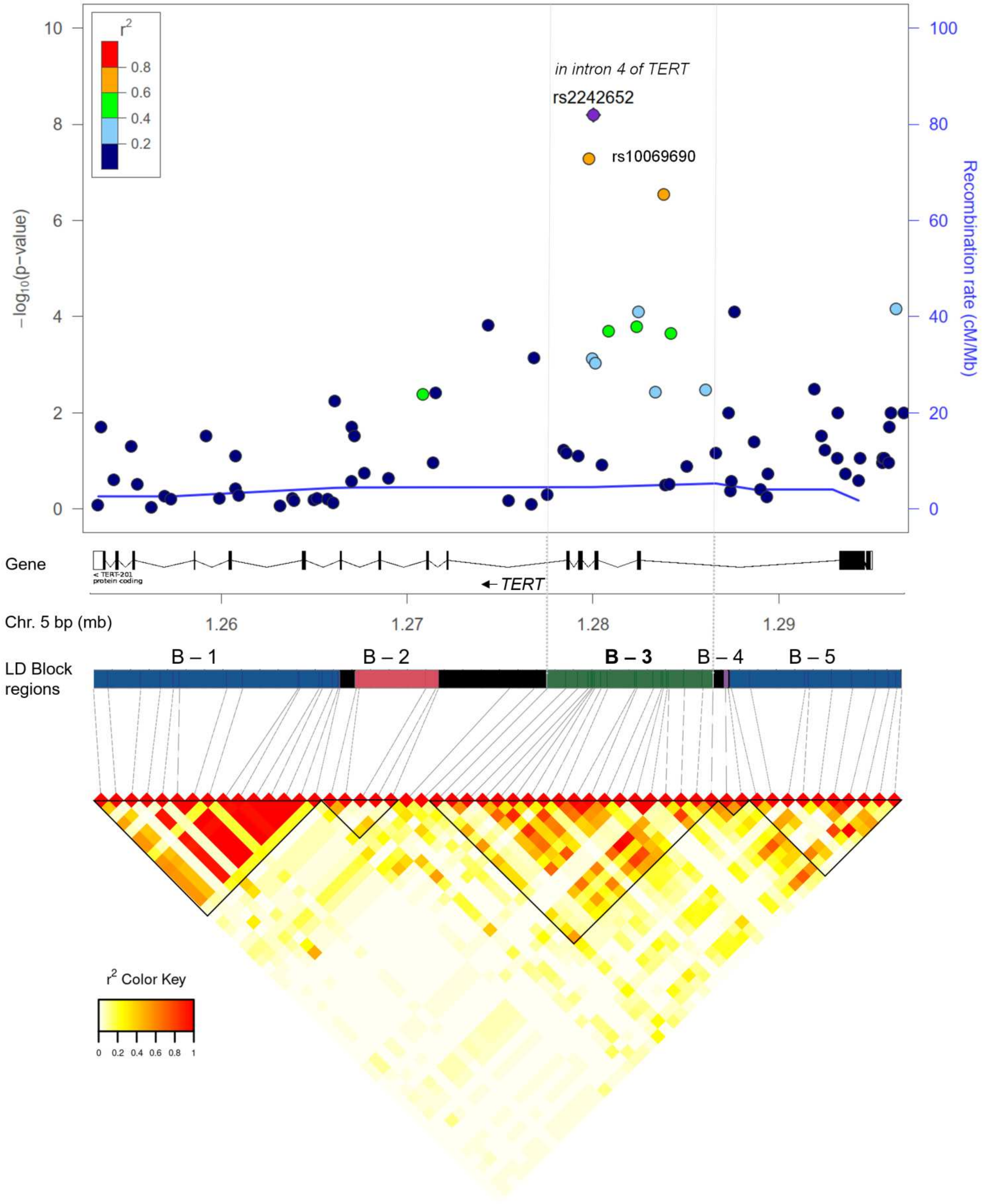

Supplementary Figure 8: Regional plot *TM6SF2* (Discovery and Replication cohorts combined)

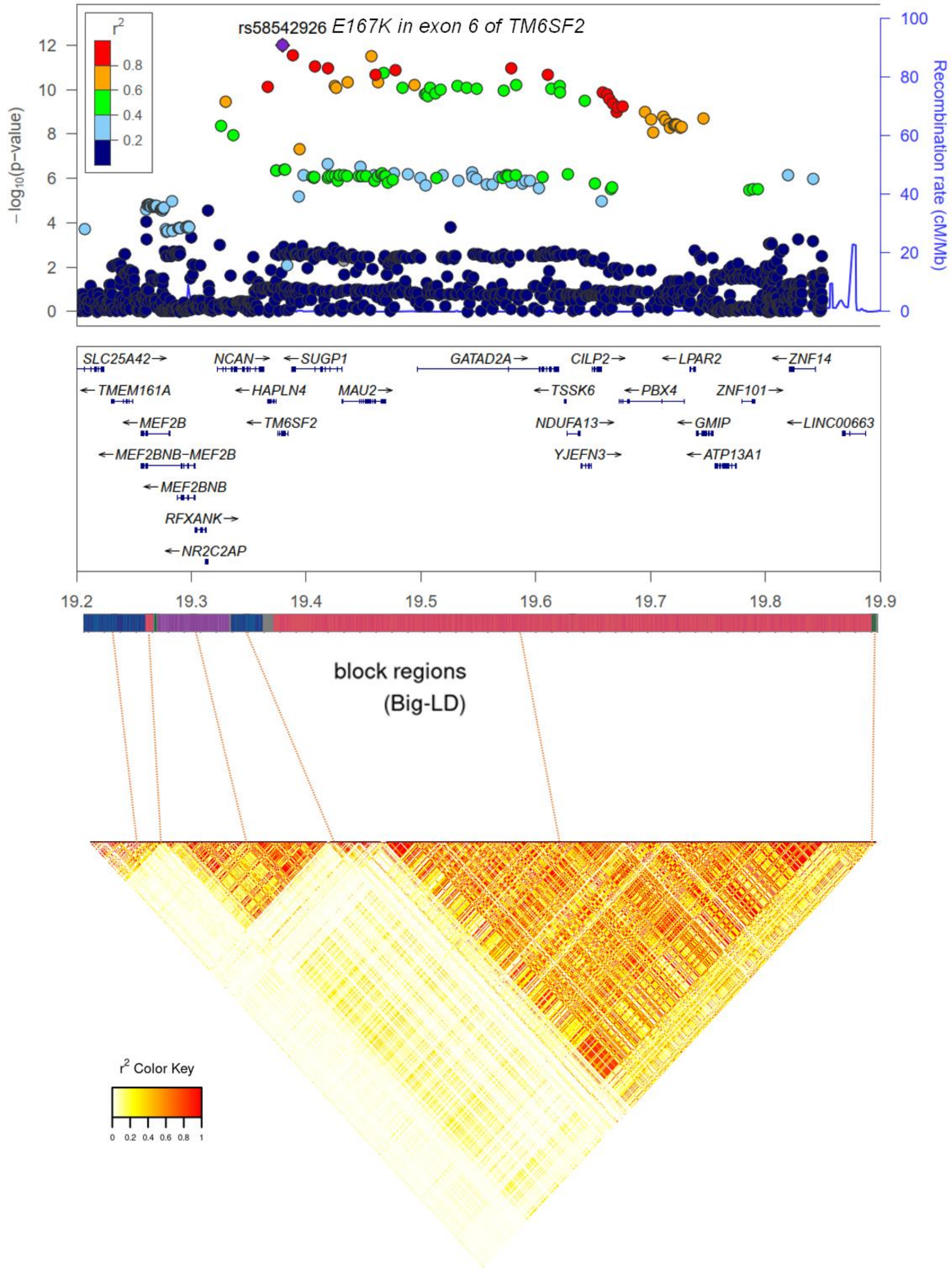

Supplementary Figure 9: Regional plot *PNPLA3* (Discovery and Replication cohorts combined)

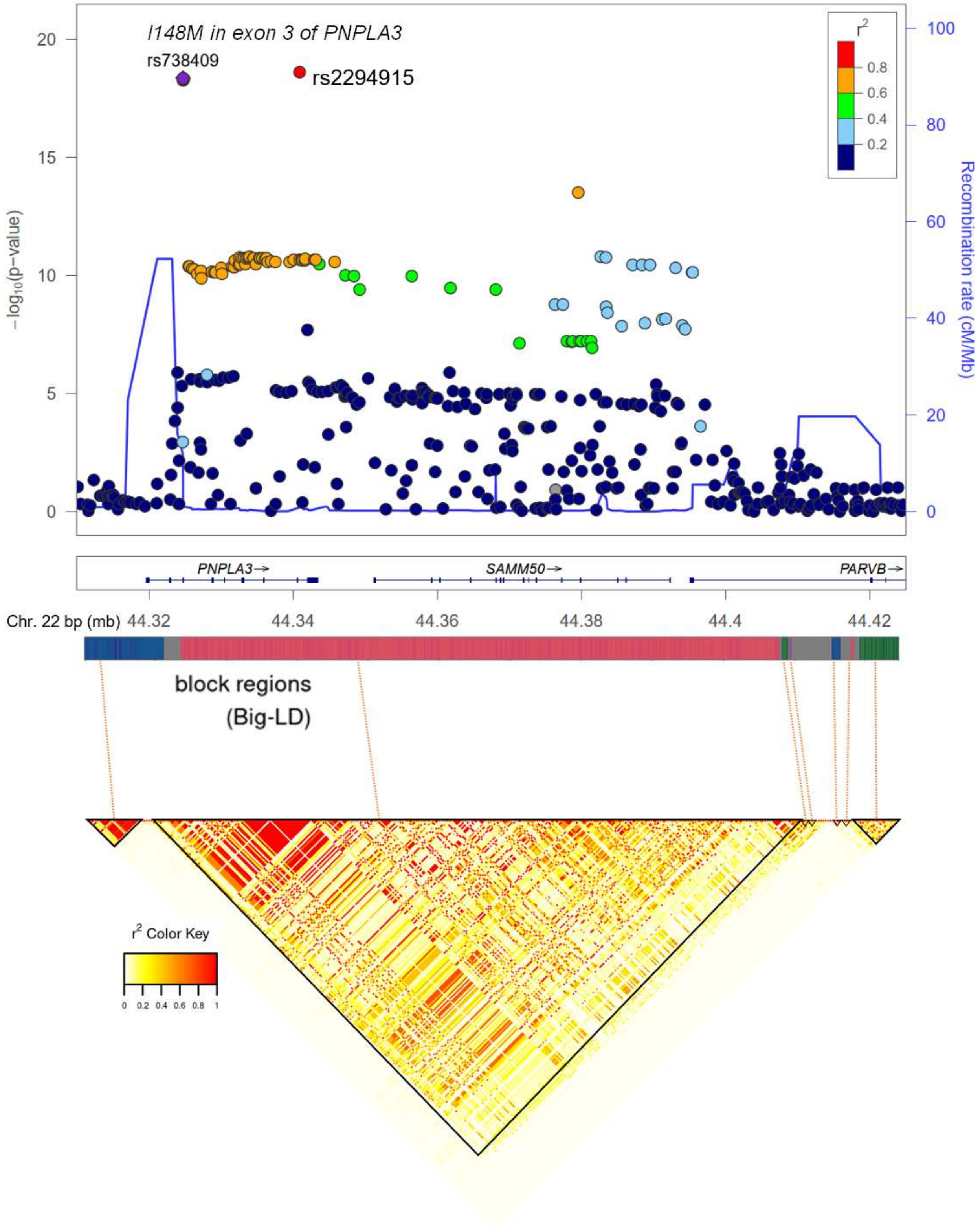

Supplementay Figure 10: Forest plots for the associations of *TERT* variant rs2242652[A allele] with the 10 most common cancer in UK Biobank and FinnGen biobank populated-based cohorts

TERT rs2242652:A

Cohort	Cancer		Beta	#case	#control	P value
FinnGen	C44	Other malignant neoplasms of skin	-0.02 (-0.06, 0.01)	13705	204070	.167
UKB	C44	Other malignant neoplasms of skin	-0.05 (-0.08, -0.03)	25168	324171	**2.85** × **10^{-5}**
FinnGen	C50	Malignant neoplasm of breast	0.03 (-0.00, 0.07)	11573	135488	.058
UKB	C50	Malignant neoplasm of breast	0.04 (0.01, 0.07)	13778	335561	**.008**
FinnGen	C61	Malignant neoplasm of prostate	-0.14 (-0.18, -0.10)	8709	104635	**5.11** × **10^{-11}**
UKB	C61	Malignant neoplasm of prostate	-0.15 (-0.19, -0.12)	11217	338122	**6.16** × **10^{-16}**
FinnGen	C18	Malignant neoplasm of colon	-0.06 (-0.12, 0.01)	2594	257811	.095
UKB	C18	Malignant neoplasm of colon	0.04 (-0.01, 0.09)	5093	344246	.114
FinnGen	C34	Malignant neoplasm of bronchus and lung	-0.04 (-0.10, 0.02)	3061	257344	.207
UKB	C34	Malignant neoplasm of bronchus and lung	-0.08 (-0.13, -0.02)	4200	345139	**.008**
FinnGen	C67	Malignant neoplasm of bladder	-0.19 (-0.27, -0.11)	1701	258704	**6.10** × **10^{-6}**
UKB	C67	Malignant neoplasm of bladder	-0.17 (-0.23, -0.10)	3316	346023	**5.82** × **10^{-7}**
FinnGen	C43	Malignant melanoma of skin	0.09 (-0.18, 0.36)	143	260262	.525
UKB	C43	Malignant melanoma of skin	0.07 (0.01, 0.12)	4217	345122	**.017**
FinnGen	C20	Malignant neoplasm of rectum	-0.06 (-0.15, 0.02)	1609	258796	.145
UKB	C20	Malignant neoplasm of rectum	-0.05 (-0.13, 0.02)	2372	346967	.158
FinnGen	C54	Malignant neoplasm of corpus uteri	-0.04 (-0.12, 0.05)	1430	145631	.430
UKB	C54	Malignant neoplasm of corpus uteri	0.03 (-0.05, 0.12)	1943	347396	.404
FinnGen	C85	Other and unspecified types of non-Hodgkin's lymphoma	0.05 (-0.08, 0.18)	643	259762	.437
UKB	C85	Other and unspecified types of non-Hodgkin's lymphoma	0.03 (-0.05, 0.11)	1832	347507	.465
FinnGen	C22	Malignant neoplasm of liver and intrahepatic bile ducts	-0.21 (-0.37, -0.05)	442	259963	**.009**
UKB	C22	Malignant neoplasm of liver and intrahepatic bile ducts	-0.14 (-0.27, -0.02)	874	348465	**.027**
UKB	C22.1	Intrahepatic bile duct carcinoma	-0.10 (-0.27, 0.08)	434	348905	.274
UKB	C22.0	Liver cell carcinoma	-0.22 (-0.42, -0.02)	383	348956	**.028**

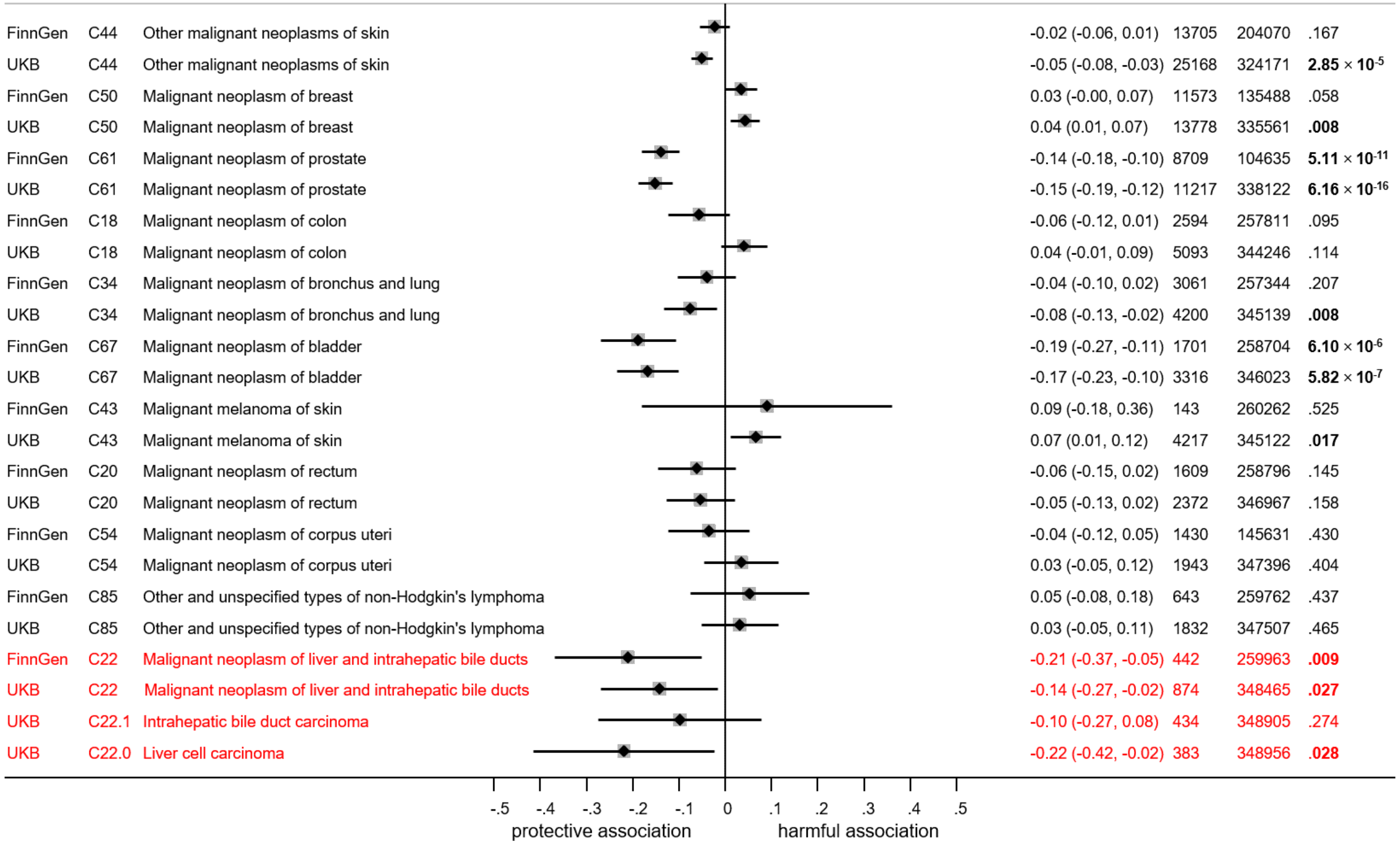

Supplementay Figure 11: The relative proportion of patients with HCC grouped by the number of risk alleles in *PNPLA3*, *TM6SF2*, and *TERT*

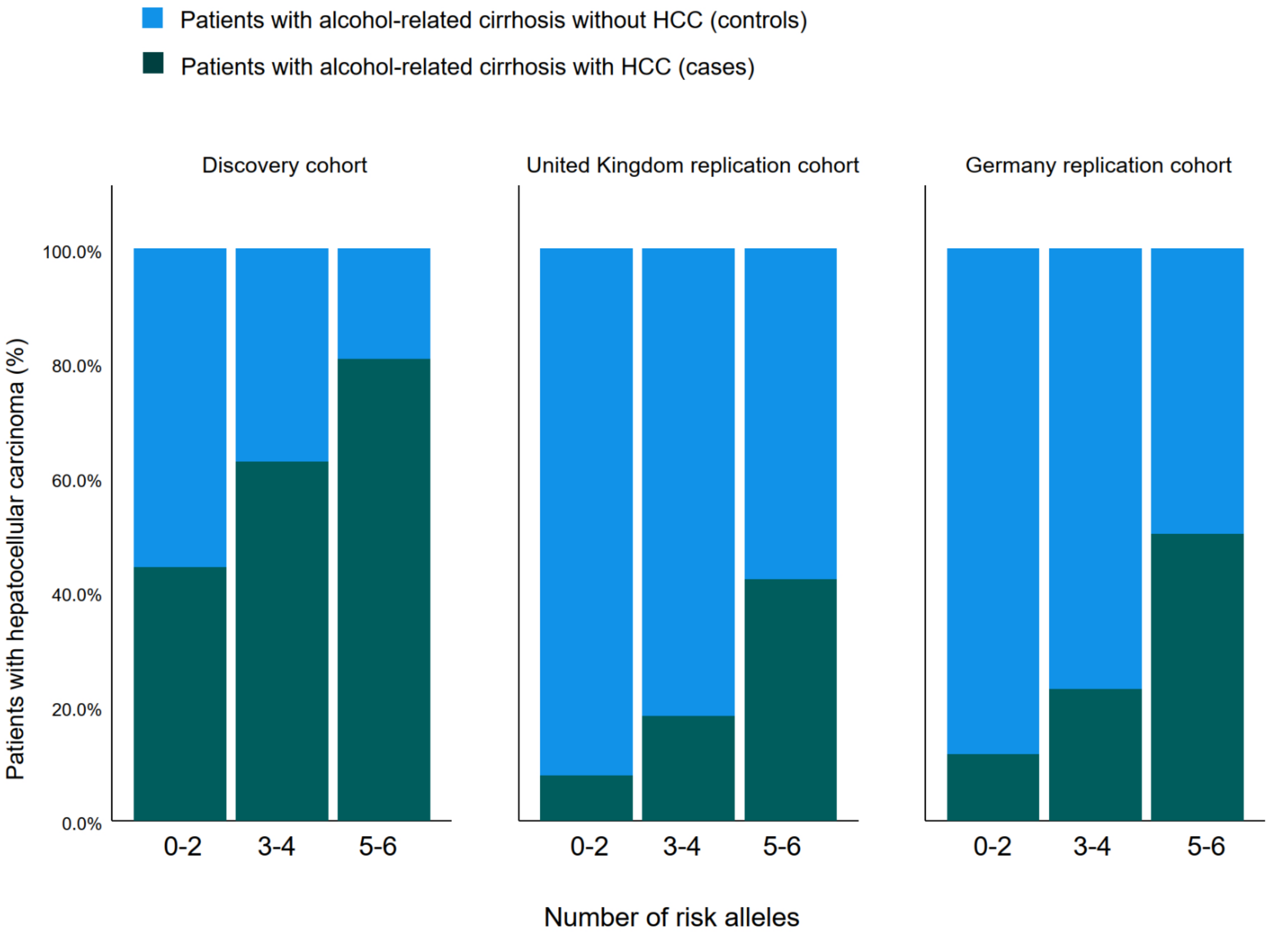

Supplementay Figure 12: Association between the number of risk alleles in *PNPLA3, TM6SF2* and *TERT* and alcohol-related hepatocellular carcinoma.

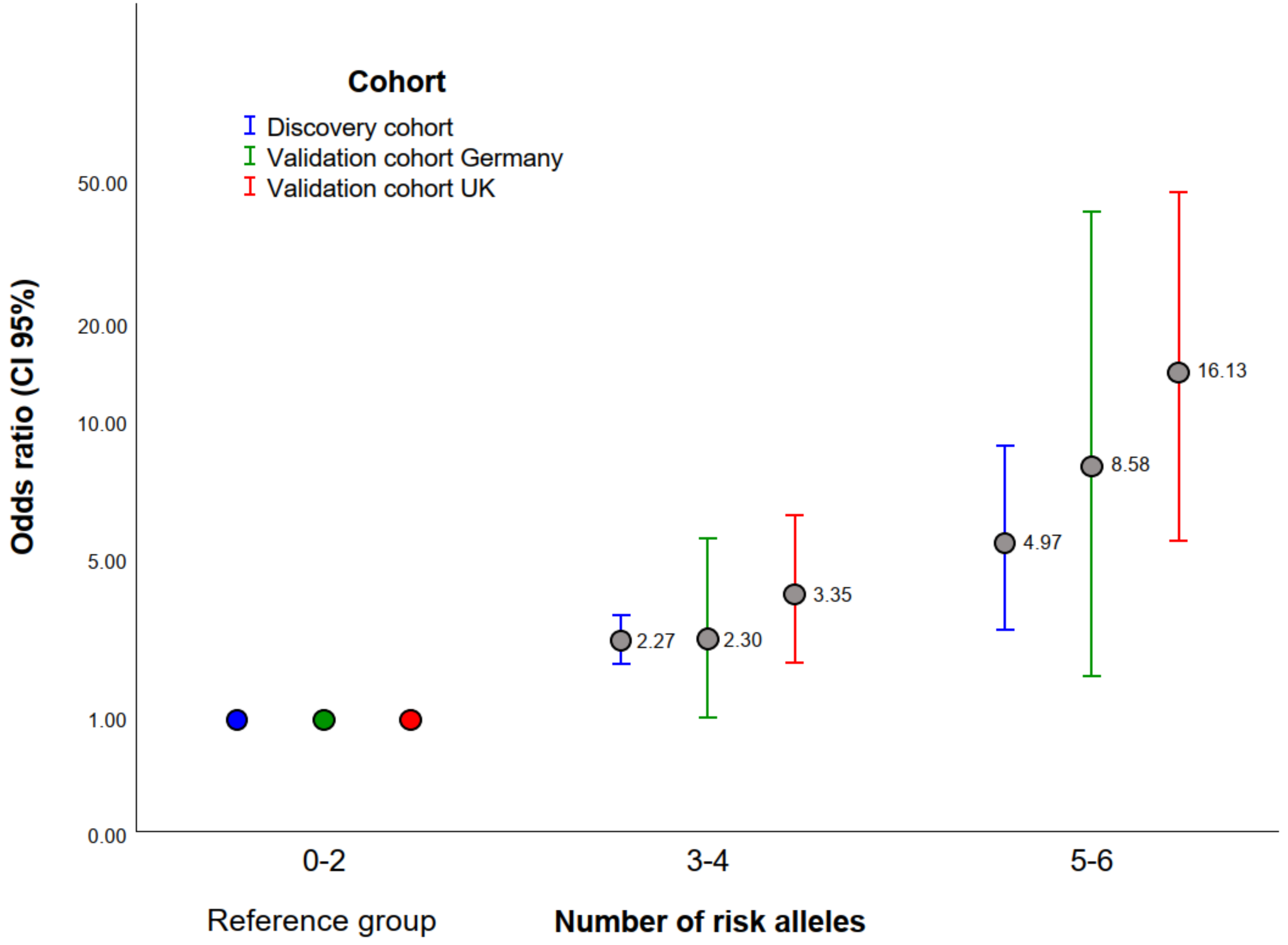

Supplementay Figure 13: HCC association results overlayed with leukocyte telomere length association results and regulation for gene *TERT*

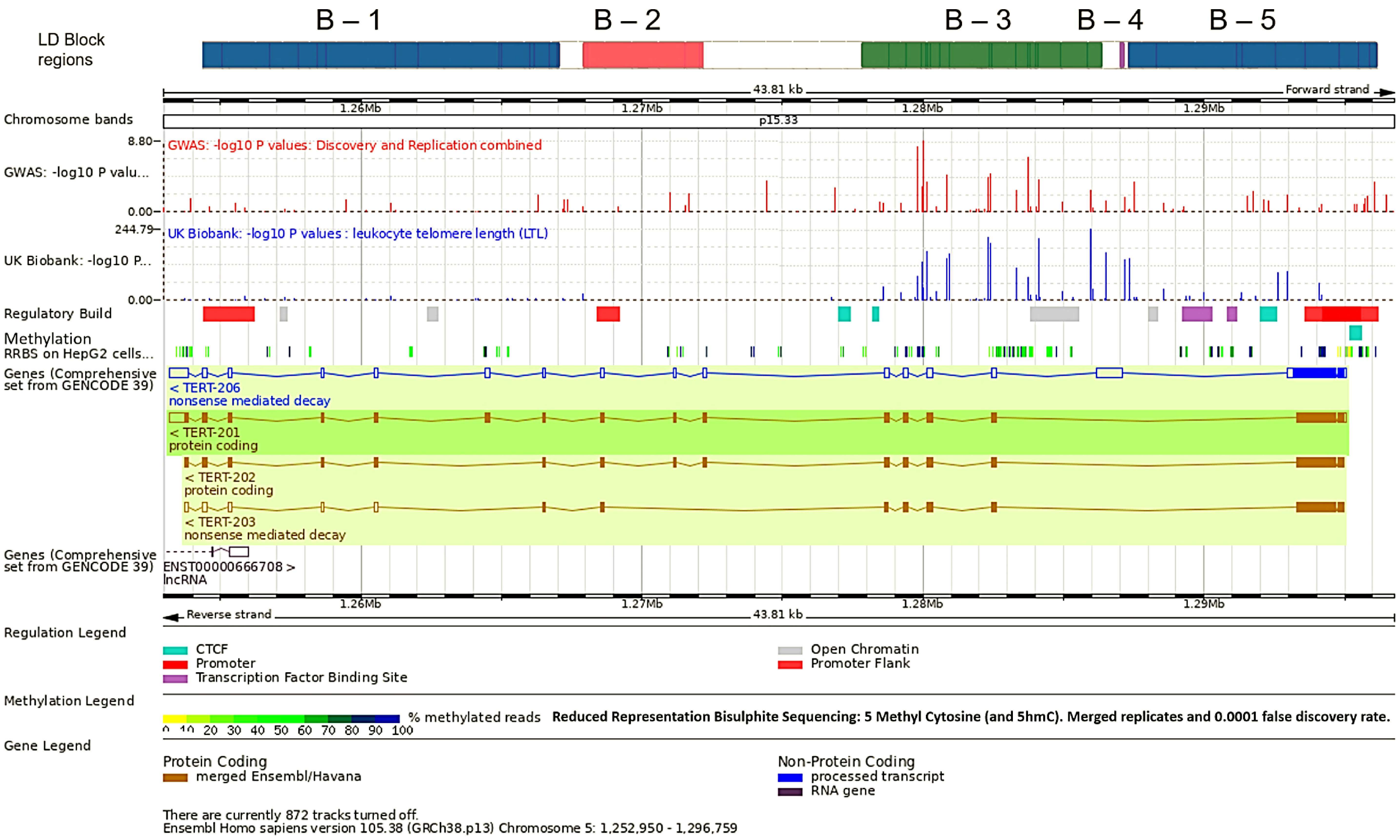

SUPPLEMENTARY TABLES

Supplementary Table 1: Power analysis for discovery GWAS (expected power to reject the null hypothesis)

Relative Risk (~OR)	1.4	1.5	1.6	1.7	1.8	1.9	2.0	2.1	2.2	2.3
**Power (p-value threshold 5×10^{-6})**										
Disease Allele Frequency*: 5%	0.019	0.066	0.167	0.329	0.525	0.709	**0.847**	**0.931**	**0.973**	**0.991**
Disease Allele Frequency: 10%	0.122	0.342	0.626	**0.846**	**0.995**	**0.991**	**1**	**1**	**1**	**1**
Disease Allele Frequency: 20%	0.453	**0.803**	**0.962**	**0.996**	1	**1**	**1**	**1**	1	**1**
Disease Allele Frequency: 30%	0.667	**0.933**	**0.994**	1	**1**	**1**	**1**	**1**	**1**	**1**
Disease Allele Frequency: 40%	0.750	**0.961**	**0.997**	**1**	**1**	**1**	**1**	**1**	**1**	**1**
**Power (p-value threshold 5×10^{-8})**										
Disease Allele Frequency: 5%	0	0.008	0.032	0.092	0.205	0.369	0.555	0.725	**0.852**	**0.931**
Disease Allele Frequency: 10%	0.020	0.098	0.554	0.554	**0.791**	**0.928**	**1**	**1**	**1**	**1**
Disease Allele Frequency: 15%	0.069	0.252	0.537	**0.790**	**0.930**	**0.983**	**0.997**	**1**	**1**	**1**
Disease Allele Frequency: 20%	0.158	0.486	**0.813**	**0.962**	**0.996**	**1**	**1**	**1**	**1**	**1**
Disease Allele Frequency: 30%	0.325	0.729	**0.947**	**0.995**	**1**	**1**	**1**	**1**	**1**	**1**
Disease Allele Frequency: 40%	0.416	**0.810**	**0.971**	**0.998**	**1**	**1**	**1**	**1**	**1**	**1**

Power of study design. Estimate of the power of the study, with 1066 cases and 844 controls using for SNPs above 5% MAF using the software Genetic Association Study (GAS) Power Calculator (https://csg.sph.umich.edu/abecasis/gas_power_calculator/index.html). *Disease allele frequency of the risk associated allele in controls. If the true odds ratio for disease in exposed subjects relative to unexposed subjects is 1.4 to 2.3, we will be able to reject the null hypothesis that this odds ratio equals 1 with probability (power) see values in table, probabilities >0.80 are shown in bold print. The Type I error probability associated with this test of this null hypothesis is 5×10^{-6} for suggestive association or 5×10^{-8} for genome-wide evidence of association.

Supplementary Table 2: Summary results for SNP rs738409 in *PNPLA3* for Discovery and Replication cohorts

Cohort	Disease status	*PNPLA3* rs738409 C>G (p.I148M)							*P*-value *	Allelic OR (95%CI)
**Discovery (Germany/Switzerland/Austria)**		**N**	**CC**	**CG**	**GG**	**Freq. C**	**Freq. G**	**HWE *P***	Chi-Square test	(Allele G)
Cases (HCC)	Cirrhosis (with HCC)	1066	302	503	261	0.519	0.481	0.073	-	-
Controls (ArC)	Cirrhosis (without HCC)	844	361	371	112	0.648	0.352	0.282	1.62×10^{-15}	1.70 (1.49-1.94)
**Replication (United Kingdom)**										
Cases (HCC)	Cirrhosis (with HCC)	70	18	36	16	0.514	0.486	0.805	-	-
Controls (ArC)	Cirrhosis (without HCC)	790	393	319	78	0.699	0.301	0.264	6.31×10^{-6}	2.20 (1.55-3.11)
Controls (AM) #1	Alcohol misusers +	340	223	108	9	0.815	0.185	0.337	2.60×10^{-14}	4.15 (2.83-6.10)
Analysis of cirrhosis risk**: Cirrhosis (without HCC) vs. Alcohol misusers +									1.26×10^{-8}	1.89 (1.51-2.36)
Replication (Germany)										
Cases (HCC)	Cirrhosis (with HCC)	42	12	18	12	0.500	0.500	0.355	-	-
Controls (ArC)	Cirrhosis (without HCC)	196	73	93	30	0.610	0.390	0.966	0.064	1.56 (0.97-2.51)
**Replication (Italy)**										
Cases (HCC)	Cirrhosis (with HCC)	36	7	19	10	0.458	0.542	0.706		
Controls (ArC)	Cirrhosis (without HCC)	36	13	16	7	0.583	0.417	0.607	0.133	1.65 (0.86-3.20)
**Risk validation in joined discovery (Germany/Switzerland) & replication (Germany) cohort**										
Cases (HCC)	Cirrhosis (with HCC)	1108	314	521	273	0.519	0.481	0.052		
Controls (ArC)	Cirrhosis (without HCC)	1040	434	464	142	0.640	0.360	0.312	6.35×10^{-16}	1.65 (1.46-1.87)
Controls (AM) #2	Alcohol misusers +	1105	664	387	54	0.776	0.224	0.804	4.56×10^{-64}	3.22 (2.82-3.66)
Population controls #3	European population controls	25362	15344	8784	1234	0.778	0.222	0.610	2.15×10^{-176}	3.26 (2.99-3.55)
Analysis of cirrhosis risk**: Cirrhosis (without HCC) vs. Alcohol misusers +									6.59×10^{-23}	1.99 (1.74-2.29)

* Significance was calculated as one degree of freedom Chi-squared test of allelic counts; + heavy drinkers with no cirrhosis and no HCC; Association results for alcohol-related HCC (cases) in comparison to alcohol-related cirrhosis (controls); and in comparison, to alcohol misusers with cirrhosis (non-cirrhosis controls) and to population controls. Genotype counts reflect the rounded sum of imputed genotype probabilities for individuals in the sample. Allelic odds ratios were calculated from 2 × 2 tables on rounded genotype counts. **Analysis of cirrhosis risk performed in individual cohorts by comparing ArC vs. alcohol misuser without cirrhosis. HWE P: Hardy Weinberg Equilibrium P value; #1 Alcohol misusers (ALC) with a clinical diagnosis of alcohol dependence recruited from Centre for Hepatology at the Royal Free Hospital, London without clinical or laboratory evidence of liver cirrhosis or HCC (data from Buch et al. Nat Genet. 2015 Dec;47(12):1443-8). #2 Alcohol misusers with a clinical diagnosis of alcohol dependence from Germany/Switzerland without clinical or laboratory evidence of liver disease or HCC (data from Buch et al. Nat Genet. 2015 Dec;47(12):1443-8); #3 North-western European population controls from gnomAD (v2.1.1).

Supplementary Table 3: Summary results for SNP rs58542926 in *TM6SF2* for Discovery and Replication cohorts

Cohort	Disease status	*TM6SF2* rs58542926 C>T (p.E167K)							*P*-value *	Allelic OR (95%CI)
**Discovery (Germany/Switzerland/Austria)**		**N**	**CC**	**CT**	**TT**	**Freq. C**	**Freq. T**	**HWE *P***	Chi-Square test	(Allele T)
Cases (HCC)	Cirrhosis (with HCC)	1066	781	253	32	0.851	0.149	0.041	-	-
Controls (ArC)	Cirrhosis (without HCC)	844	710	125	9	0.915	0.085	0.191	1.61×10^{-9}	1.89 (1.53-2.33)
**Replication (United Kingdom)**										
Cases (HCC)	Cirrhosis (with HCC)	70	45	20	5	0.786	0.214	0.205	-	-
Controls (ArC)	Cirrhosis (without HCC)	790	648	135	7	0.906	0.094	0.991	8.35×10^{-6}	2.55 (1.65-3.95)
Controls (AM) #1	Alcohol misusers +	340	296	43	1	0.934	0.066	0.668	3.09×10^{-8}	3.85 (2.32-6.37)
Analysis of cirrhosis risk**: Cirrhosis (without HCC) vs. Alcohol misusers +									0.0286	1.47 (1.04-2.08)
**Replication (Germany)**										
Cases (HCC)	Cirrhosis (with HCC)	42	33	7	2	0.869	0.131	0.083	-	-
Controls (ArC)	Cirrhosis (without HCC)	196	159	32	5	0.893	0.107	0.040	0.529	1.26 (0.62-2.55)
**Replication (Italy)**										
Cases (HCC)	Cases (HCC)	36	31	5	0	0.931	0.069	0.654		
Controls (ArC)	Controls (ArC)	36	28	8	0	0.889	0.111	0.453	0.383	0.60 (0.19-1.92)
**Risk validation in joined discovery (Germany/Switzerland) & replication (Germany) cohort**										
Cases (HCC)	Cirrhosis (with HCC)	1108	814	260	34	0.852	0.148	0.021		
Controls (ArC)	Cirrhosis (without HCC)	1040	869	157	14	0.911	0.089	0.027	2.42×10^{-9}	1.78 (1.47-2.15)
Controls (AM) #2	Alcohol misusers +	1115	987	124	4	0.941	0.059	0.960	2.41×10^{-22}	2.76 (2.24-3.41)
Population controls #3	European population controls	23816	20633	3082	101	0.931	0.069	0.218	9.57×10^{-45}	2.35 (2.08-2.65)
Analysis of cirrhosis risk**: Cirrhosis (without HCC) vs. Alcohol misusers +									1.44×10^{-4}	1.58 (1.25-2.01)

* Significance was calculated as one degree of freedom Chi-squared test of allelic counts. + heavy drinkers with no cirrhosis and no HCC; Association results for alcohol-related HCC (cases) in comparison to alcohol-related cirrhosis (controls); and in comparison, to alcohol misusers with cirrhosis (non-cirrhosis controls) and to population controls. Genotype counts reflect the rounded sum of imputed genotype probabilities for individuals in the sample. Allelic odds ratios were calculated from 2 × 2 tables on rounded genotype counts. **Analysis of cirrhosis risk performed in individual cohorts by comparing ArC vs. alcohol misuser without cirrhosis. HWE P: Hardy Weinberg Equilibrium P value; #1 Alcohol misusers (ALC) with a clinical diagnosis of alcohol dependence recruited from Centre for Hepatology at the Royal Free Hospital, London without clinical or laboratory evidence of liver cirrhosis or HCC (data from Buch et al. Nat Genet. 2015 Dec;47(12):1443-8). #2 Alcohol misusers with a clinical diagnosis of alcohol dependence from Germany/Switzerland without clinical or laboratory evidence of liver disease or HCC (data from Buch et al. Nat Genet. 2015 Dec;47(12):1443-8); #3 North-western European population controls from gnomAD (v2.1.1).

Supplementary Table 4: Summary results for SNP rs2242652 in *TERT* for Discovery and Replication cohorts

Cohort	Disease status	*TERT* rs2242652 G>A							*P*-value *	Allelic OR (95%CI)
**Discovery (Germany/Switzerland/Austria)**		**N**	**GG**	**GA**	**AA**	**Freq. G**	**Freq. A**	**HWE *P***	Chi-Square test	2 × 2 table (A allele)
Cases (HCC)	Cirrhosis (with HCC)	1066	803	243	20	0.867	0.133	0.746	-	-
Controls (ArC)	Cirrhosis (without HCC)	844	555	256	33	0.809	0.191	0.610	1.07×10^{-6}	0.65 (0.55-0.77)
**Replication (United Kingdom)**										
Cases (HCC)	Cirrhosis (with HCC)	70	57	11	2	0.893	0.107	0.135	-	-
Controls (ArC)	Cirrhosis (without HCC)	783	500	253	30	0.800	0.200	0.775	7.64×10^{-3}	0.48 (0.28-0.83)
Controls (AM) #1	Alcohol misusers +	341	221	112	8	0.812	0.188	0.154	2.20×10^{-2}	0.52 (0.29-0.92)
Analysis of cirrhosis risk**: Cirrhosis (without HCC) vs. Alcohol misusers +									0.503	1.08 (0.86-1.36)
**Replication (Germany)**										
Cases (HCC)	Cirrhosis (with HCC)	42	32	10	0	0.881	0.119	0.381	-	-
Controls (ArC)	Cirrhosis (without HCC)	196	119	71	6	0.788	0.212	0.233	0.052	0.50 (0.25-1.02)
**Replication (Italy)**										
Cases (HCC)	Cirrhosis (with HCC)	36	27	9	0	0.875	0.125	0.391	-	-
Controls (ArC)	Cirrhosis (without HCC)	36	27	8	1	0.861	0.139	0.670	0.806	0.89 (0.34-2.33)
**Risk validation in joined discovery (Germany/Switzerland) & replication (Germany) cohort**										
Cases (HCC)	Cirrhosis (with HCC)	1108	835	253	20	0.868	0.132	0.869		
Controls (ArC)	Cirrhosis (without HCC)	1040	674	327	39	0.805	0.195	0.932	2.87×10^{-8}	0.63 (0.53-0.74)
Controls (AM) #2	Alcohol misusers +	1179	760	367	52	0.800	0.200	0.366	9.43×10^{-10}	0.61 (0.52-0.72)
Population controls #3	European population controls	4290	2797	1344	149	0.809	0.191	0.423	9.64×10^{-11}	0.64 (0.56-0.74)
Analysis of cirrhosis risk**: Cirrhosis (without HCC) vs. Alcohol misusers +									0.511	0.95 (0.82-1.11)

* Significance was calculated as one degree of freedom Chi-squared test of allelic counts. + heavy drinkers with no cirrhosis and no HCC; Association results for alcohol-related HCC (cases) in comparison to ArC (controls); and in comparison, to alcohol misusers with cirrhosis (non-cirrhosis controls) and to population controls. Genotype counts reflect the rounded sum of imputed genotype probabilities for individuals in the sample. Allelic odds ratios were calculated from 2 × 2 tables on rounded genotype counts. **Analysis of cirrhosis risk performed in individual cohorts by comparing ArC vs. alcohol misuser without cirrhosis. HWE P: Hardy Weinberg Equilibrium P value; #1 Alcohol misusers (ALC) with a clinical diagnosis of alcohol dependence recruited from Centre for Hepatology at the Royal Free Hospital, London without clinical or laboratory evidence of liver cirrhosis or HCC (data from Buch et al. Nat Genet. 2015 Dec;47(12):1443-8). #2 Alcohol misusers with a clinical diagnosis of alcohol dependence from Germany/Switzerland without clinical or laboratory evidence of liver disease or HCC (data from Buch et al. Nat Genet. 2015); #3 North-western European population controls from gnomAD (v2.1.1).

Supplementary Table 5: Replicated and known HCC risk loci successively adjusted for sex, age, BMI and type 2 diabetes status

			**Discovery cohort**				**Combined (Discovery and Replication cohorts)**			
Covariate adjustments			PCs	PCs, sex, age	PCs, sex, age, BMI	PCs, sex, age, BMI, Diabetes	PCs	PCs, sex, age	PCs, sex, age, BMI	PCs, sex, age, BMI, Diabetes
N (cases)			1066	1066	740	616	1214	1214	857	731
N (controls)			844	844	765	423	1866	1866	1684	1337
Effective sample size*			1884	1884	1505	1003	2352	2352	1889	1382
Locus	Chr.	SNP ID (effect allele)	Adjusted OR[a] (*P value*)	Adjusted OR[b] (*P value*)	Adjusted OR[c] (*P value*)	Adjusted OR[d] (*P value*)	Adjusted OR[a] (*P value*)[a,e]	Adjusted OR[b] (*P value*)[e]	Adjusted OR[c] (*P value*)[e]	Adjusted OR[d] (*P value*)[e]
PNPLA3	22	rs738409	1.71 (1.49-1.96)	1.75 (1.51-2.03)	1.87 (1.58-2.22)	2.07 (1.69-2.54)	1.74 (1.54-1.97)	1.77 (1.55-2.02)	1.86 (1.60-2.16)	1.99 (1.68-2.37)
p.I148M		(G)	7.23×10^{-15}	1.67×10^{-13}	3.22×10^{-13}	3.21×10^{-12}	4.31×10^{-19}	5.35×10^{-17}	4.93×10^{-16}	5.62×10^{-15}
TM6SF2	19	rs58542926	1.94 (1.56-2.42)	1.93 (1.51-2.45)	2.03 (1.55-2.65)	1.98 (1.43-2.74)	1.98 (1.64-2.40)	1.99 (1.61-2.44)	2.08 (1.66-2.61)	2.07 (1.60-2.68)
p.E167K		(T)	2.81×10^{-9}	1.21×10^{-7}	2.01×10^{-7}	3.43×10^{-5}	1.00×10^{-12}	8.80×10^{-11}	2.24×10^{-10}	3.63×10^{-8}
TERT	5	rs2242652	0.64 (0.53-0.76)	0.64 (0.52-0.78)	0.68 (0.55-0.85)	0.69 (0.54-0.90)	0.61 (0.52-0.72)	0.60 (0.50-0.72)	0.64 (0.52-0.78)	0.63 (0.50-0.79)
		(A)	7.87×10^{-7}	9.28×10^{-6}	5.19×10^{-4}	5.89×10^{-3}	6.40×10^{-9}	4.08×10^{-8}	9.11×10^{-6}	7.94×10^{-5}
WNT3A-WNT9A	1	rs708113	0.97 (0.85-1.11)	0.97 (0.83-1.13)	0.93 (0.79-1.10)	0.94 (0.77-1.16)	0.99 (0.87-1.14)	0.98 (0.86-1.13)	0.92 (0.79-1.07)	0.91 (0.77-1.09)
		(T)	0.666	0.680	0.402	0.579	0.932	0.800	0.279	0.321
APOE	19	rs429358	0.74 (0.60-0.91)	0.76 (0.60-0.96)	0.74 (0.57-0.96)	0.68 (0.50-0.93)	0.71 (0.58-0.86)	0.72 (0.58-0.89)	0.68 (0.54-0.87)	0.63 (0.47-0.83)
		(C)	5.44×10^{-3}	2.35×10^{-2}	2.50×10^{-2}	1.59×10^{-2}	5.76×10^{-4}	2.72×10^{-3}	2.17×10^{-3}	1.07×10^{-3}
HSD17B13	4	rs72613567	0.81 (0.69-0.95)	0.84 (0.71-1.01)	0.85 (0.69-1.03)	0.79 (0.63-1.01)	0.85 (0.73-0.99)	0.82 (0.71-0.95)	0.83 (0.69-0.99)	0.79 (0.64-0.96)
		(TA)	8.95×10^{-3}	5.91×10^{-2}	0.102	5.91×10^{-2}	3.63×10^{-2}	7.22×10^{-3}	3.73×10^{-2}	1.96×10^{-2}

Multivariate logistic regression analyses of susceptibility loci for alcohol-related HCC in comparison to ArC, under the additive genetic model; PCs: principal components of of genetic ancestry; all analyses were adjusted by top 15 PCs; Chr., chromosome; OR, odds ratio. Odds ratios are provided with 95% confidence interval; Phenotypic information for BMI and type 2 diabetes status was available for cases and controls accordingly to Table 1.

[a] adjusted for top 15 principal components of genetic ancestry
[b] adjusted for age, sex, and top 15 principal components of genetic ancestry
[c] adjusted for age, sex, BMI, and top 15 principal components of genetic ancestry
[d] adjusted for age, sex, BMI, type II diabetes mellitus and top 15 principal components of genetic ancestry
[e] derived from fixed effect model summary estimates of discovery and replication cohorts
* Effective sample size = 4 / (1/number of cases + 1/number of controls); in the combined analysis the sum of the effective sample sizes of each cohort is reported

Supplementary Table 6: Meta-analysis GWAS association results for loci with P<1×10^{-7}

				EA	Meta-analysis of stage 1 and 2 cohorts (pc adjusted)			
*Locus**	CHR	Position	SNP		P-value [a]	OR	HetISq	Direction
TERT	5	1280028	rs2242652	A	6.40E-09	0.61	0	----
TERT	5	1279790	rs10069690	T	5.19E-08	0.66	0	----
TM6SF2	19	19379549	rs58542926	T	1.00E-12	1.98	43.25	+++-
TM6SF2	19	19388500	rs8107974	A	2.93E-12	1.94	44.39	---+
TM6SF2	19	19456917	rs58489806	T	3.04E-12	1.88	32.17	+++-
TM6SF2	19	19407718	rs10401969	T	8.98E-12	1.89	40.74	---+
TM6SF2	19	19419071	rs739846	A	1.09E-11	1.9	41.15	+++-
TM6SF2	19	19578743	rs73002956	A	1.09E-11	1.88	1.57	---+
TM6SF2	19	19477877	rs56255430	A	1.28E-11	1.88	4.72	---+
TM6SF2	19	19467545	rs2285626	T	1.83E-11	1.75	54.01	+++-
TM6SF2	19	19610596	rs3794991	T	2.07E-11	1.86	3.28	+++-
TM6SF2	19	19460541	rs73001065	C	2.19E-11	1.96	28.52	+++-
TM6SF2	19	19436229	rs111234557	C	4.46E-11	1.75	59.43	---+
TM6SF2	19	19462702	rs11672355	C	4.67E-11	1.75	59.19	+++-
TM6SF2	19	19582992	rs73002960	T	6.20E-11	1.72	44.85	+++-
TM6SF2	19	19494483	rs150268548	A	6.32E-11	1.95	0	+++-
TM6SF2	19	19621004	rs56273306	T	6.60E-11	1.72	44.48	---+
TM6SF2	19	19531910	rs11668386	A	6.61E-11	1.72	43.7	---+
TM6SF2	19	19425025	rs57962361	T	6.88E-11	1.74	59.86	+++-
TM6SF2	19	19366632	rs72999033	T	7.69E-11	1.97	10.34	+++-
TM6SF2	19	19539891	rs8182472	T	8.03E-11	1.72	44.23	---+
TM6SF2	19	19426181	rs11668104	A	8.06E-11	1.73	60.03	+++-
TM6SF2	19	19484008	rs59148799	A	8.25E-11	1.72	44	---+
TM6SF2	19	19508013	rs10424702	A	8.34E-11	1.71	42.5	---+
TM6SF2	19	19613622	rs57009615	A	8.91E-11	1.7	28.05	---+
TM6SF2	19	19548643	rs79954596	T	9.47E-11	1.71	43.81	---+
TM6SF2	19	19517169	rs188552254	A	9.61E-11	1.71	41.81	---+
TM6SF2	19	19572220	rs28720066	T	1.09E-10	1.71	45.39	+++-
TM6SF2	19	19621197	rs113365218	A	1.38E-10	1.72	49.71	+++-
TM6SF2	19	19658472	rs16996148	T	1.38E-10	1.82	0	+++-
TM6SF2	19	19512657	rs10408596	A	1.48E-10	1.7	44.39	---+
TM6SF2	19	19505087	rs10415849	T	1.51E-10	1.7	44.45	+++-
TM6SF2	19	19503573	rs10408875	T	1.61E-10	1.7	43.6	---+
TM6SF2	19	19662220	rs17216525	T	1.65E-10	1.83	0	+++-
TM6SF2	19	19506092	rs56241616	T	1.93E-10	1.7	44.7	+++-
TM6SF2	19	19664077	rs17216588	T	2.69E-10	1.81	0	+++-
TM6SF2	19	19642795	rs56397647	T	3.22E-10	1.7	9.17	+++-
TM6SF2	19	19329924	rs2228603	T	3.47E-10	1.85	0	+++-
TM6SF2	19	19667254	rs143988316	T	4.28E-10	1.8	0	+++-
TM6SF2	19	19675696	rs73004933	T	5.83E-10	1.8	0	+++-
TM6SF2	19	19671266	rs73004926	T	6.08E-10	1.8	0	+++-
TM6SF2	19	19670610	rs150824230	A	9.71E-10	1.78	0	+++-
TM6SF2	19	19695228	rs73004951	T	9.80E-10	1.78	0	+++-
TM6SF2	19	19711139	rs73004959	T	1.57E-09	1.76	0	+++-
TM6SF2	19	19746151	rs2304128	T	2.00E-09	1.77	0	++++
TM6SF2	19	19700552	rs12608729	T	2.26E-09	1.75	0	+++-
TM6SF2	19	19713069	rs73004962	A	2.47E-09	1.75	0	---+
TM6SF2	19	19721722	rs12610185	A	3.56E-09	1.73	0	+++-
TM6SF2	19	19716558	rs73004966	T	3.57E-09	1.74	0	+++-

TM6SF2	19	19720399	rs57504626	T	3.81E-09	1.74	0	+++-
TM6SF2	19	19720788	rs16996185	T	3.81E-09	1.74	0	---+
TM6SF2	19	19721976	rs12610191	T	3.81E-09	1.74	0	+++-
TM6SF2	19	19723215	rs10500212	T	3.81E-09	1.73	0	+++-
TM6SF2	19	19325963	rs3761077	T	4.38E-09	1.68	0	+++-
TM6SF2	19	19727152	rs73004975	A	4.66E-09	1.72	0	---+
TM6SF2	19	19726022	rs58847337	A	5.05E-09	1.72	0	+++-
TM6SF2	19	19717056	rs73004967	A	5.40E-09	1.82	0	---+
TM6SF2	19	19702384	rs17217098	A	8.56E-09	1.81	0	+++-
TM6SF2	19	19336608	rs2238675	T	1.18E-08	1.62	0	+++-
TM6SF2	19	19394368	rs138295924	A	4.64E-08	1.92	0	---+
PNPLA3	22	44340904	rs2294915	T	2.44E-19	1.75	0	++++
PNPLA3	22	44324727	rs738409	G	4.31E-19	1.74	0	++++
PNPLA3	22	44324855	rs3747207	A	4.71E-19	1.77	0	++++
PNPLA3	22	44324730	rs738408	T	5.62E-19	1.77	0	++++
PNPLA3	22	44379565	rs2294922	C	3.11E-14	1.62	16.85	++++
PNPLA3	22	44333968	rs2896020	T	1.62E-11	1.56	0	----
PNPLA3	22	44382684	rs2294927	T	1.63E-11	1.51	0	----
PNPLA3	22	44332570	rs2281135	A	1.70E-11	1.55	0	++++
PNPLA3	22	44333694	rs2896019	T	1.70E-11	1.55	0	----
PNPLA3	22	44383400	rs6006602	T	1.73E-11	1.51	0	++++
PNPLA3	22	44334476	rs4823176	T	1.86E-11	1.56	0	----
PNPLA3	22	44333172	rs2072906	A	1.87E-11	1.56	0	----
PNPLA3	22	44333479	rs2072905	C	1.87E-11	1.56	0	----
PNPLA3	22	44333945	rs2401512	C	1.87E-11	1.56	0	----
PNPLA3	22	44335331	rs16991175	T	1.87E-11	1.56	0	----
PNPLA3	22	44335406	rs35621602	A	1.87E-11	1.56	0	++++
PNPLA3	22	44335416	rs34352134	T	1.87E-11	1.56	0	++++
PNPLA3	22	44335744	rs2073081	T	1.87E-11	1.56	0	----
PNPLA3	22	44334529	rs4823178	T	1.87E-11	1.56	0	----
PNPLA3	22	44335453	rs34376930	T	1.87E-11	1.56	0	++++
PNPLA3	22	44334486	rs4823177	T	1.88E-11	1.56	0	----
PNPLA3	22	44332878	rs34879941	T	1.90E-11	1.56	0	++++
PNPLA3	22	44336310	rs1010022	A	1.91E-11	1.56	0	----
PNPLA3	22	44336098	rs1010023	T	1.96E-11	1.56	0	----
PNPLA3	22	44341666	rs13055900	A	2.00E-11	1.55	0	----
PNPLA3	22	44341672	rs13055874	T	2.04E-11	1.55	0	----
PNPLA3	22	44340086	rs36069781	T	2.10E-11	1.55	0	++++
PNPLA3	22	44341193	rs4823179	T	2.15E-11	1.55	0	----
PNPLA3	22	44340922	rs2294916	T	2.16E-11	1.55	0	----
PNPLA3	22	44343151	rs1810508	A	2.18E-11	1.55	0	----
PNPLA3	22	44342969	rs2008451	T	2.18E-11	1.55	0	----
PNPLA3	22	44341606	rs4823181	T	2.28E-11	1.55	0	----
PNPLA3	22	44341298	rs4823180	A	2.28E-11	1.55	0	++++
PNPLA3	22	44331943	rs1883349	A	2.35E-11	1.55	0	++++
PNPLA3	22	44336957	rs73176497	A	2.49E-11	1.55	0	++++
PNPLA3	22	44339526	rs13056555	C	2.66E-11	1.55	0	----
PNPLA3	22	44336496	rs8142145	T	2.66E-11	1.55	0	----
PNPLA3	22	44337533	rs926633	A	2.66E-11	1.55	0	++++
PNPLA3	22	44345771	rs13054885	A	2.73E-11	1.55	0	++++
PNPLA3	22	44343626	rs12484795	A	3.26E-11	1.55	0	----
PNPLA3	22	44334842	rs2281293	T	3.29E-11	1.55	0	----
PNPLA3	22	44333370	rs2076207	A	3.33E-11	1.55	0	----
PNPLA3	22	44332653	rs2072907	C	3.37E-11	1.55	0	----
PNPLA3	22	44387108	rs1986095	A	3.55E-11	1.5	0	----
PNPLA3	22	44389514	rs2235778	T	3.57E-11	1.5	0	----

PNPLA3	22	44388417	rs3788604	A	3.58E-11	1.5	0	----
PNPLA3	22	44332477	rs2281138	T	3.65E-11	1.55	0	----
PNPLA3	22	44332493	rs2281137	T	3.65E-11	1.55	0	----
PNPLA3	22	44331513	rs1997693	C	4.24E-11	1.54	0	----
PNPLA3	22	44325631	rs12484809	T	4.26E-11	1.56	0	++++
PNPLA3	22	44325565	rs12484801	T	4.26E-11	1.56	0	++++
PNPLA3	22	44325516	rs12485100	T	4.28E-11	1.56	0	++++
PNPLA3	22	44331778	rs13056638	C	4.29E-11	1.54	0	----
PNPLA3	22	44331815	rs1883348	C	4.29E-11	1.54	0	----
PNPLA3	22	44330031	rs1977080	T	4.76E-11	1.55	0	++++
PNPLA3	22	44393075	rs6006473	T	4.91E-11	1.49	0	++++
PNPLA3	22	44325996	rs12483959	A	5.14E-11	1.56	0	++++
PNPLA3	22	44326272	rs9625962	T	5.36E-11	1.56	0	----
PNPLA3	22	44327179	rs16991158	A	6.36E-11	1.54	0	++++
PNPLA3	22	44327192	rs36055245	A	6.36E-11	1.54	0	----
PNPLA3	22	44328730	rs4823173	A	7.00E-11	1.54	0	++++
PNPLA3	22	44329078	rs2076211	T	7.14E-11	1.54	0	++++
PNPLA3	22	44395451	rs1007863	T	7.26E-11	1.49	0	----
PNPLA3	22	44329275	rs2294433	A	7.29E-11	1.54	0	++++
PNPLA3	22	44395389	rs2281292	A	7.29E-11	1.48	0	----
PNPLA3	22	44330128	rs1977081	T	8.72E-11	1.54	0	----
PNPLA3	22	44326700	rs11090617	T	8.76E-11	1.54	0	++++
PNPLA3	22	44347251	rs2092501	A	1.02E-10	1.54	0	++++
PNPLA3	22	44348446	rs34912062	T	1.06E-10	1.54	0	++++
PNPLA3	22	44356468	rs56373884	A	1.09E-10	1.53	0	++++
PNPLA3	22	44327273	rs12484700	A	1.31E-10	1.53	0	----
PNPLA3	22	44361842	rs2294921	T	3.46E-10	1.51	6.21	+++-
PNPLA3	22	44349236	rs1474745	T	3.81E-10	1.51	7.06	---+
PNPLA3	22	44368122	rs3761472	A	3.84E-10	1.51	10.13	---+
PNPLA3	22	44376335	rs67450864	T	1.67E-09	1.44	28.91	++++
PNPLA3	22	44377442	rs4823182	A	1.68E-09	1.44	28	----
PNPLA3	22	44391686	rs2143571	A	6.56E-09	1.47	0	++++
PNPLA3	22	44391234	rs2281298	A	7.20E-09	1.47	0	++++
PNPLA3	22	44388817	rs3827385	T	1.02E-08	1.46	0	---+
PNPLA3	22	44394019	rs2401514	A	1.31E-08	1.46	0	+++-
PNPLA3	22	44385594	rs2073079	A	1.42E-08	1.46	0	---+
PNPLA3	22	44394402	rs2073080	T	1.87E-08	1.45	0	+++-
PNPLA3	22	44341986	rs2294917	T	2.00E-08	0.65	0	++++
PNPLA3	22	44378809	rs2235777	T	5.95E-08	1.45	45.46	+++-
PNPLA3	22	44380767	rs12167845	T	5.95E-08	1.45	44.77	---+
PNPLA3	22	44380009	rs9626079	A	6.07E-08	1.44	44.82	---+
PNPLA3	22	44379740	rs2294923	A	6.08E-08	1.44	44.81	+++-
PNPLA3	22	44377999	rs2235776	T	6.12E-08	1.44	45.71	+++-
PNPLA3	22	44381340	rs4823108	T	6.21E-08	1.44	45.54	---+
PNPLA3	22	44378672	rs4823183	A	6.70E-08	1.44	45.69	+++-

Abbreviations: SNP: single nucleotide polymorphism; Chr: chromosome; OR: odds ratio; HetISq: I^2-measure of percentage of between cohort heterogeneity Meta; P value: Significance derived from a fixed effect meta-analysis.[a] Odds ratio and *P* value adjusted for top 15 principal components of genetic ancestry; * risk loci *TM6SF2* and *PNPLA3* annotate to multiple genes not listed in detail

Supplementary Table 7: Known alcohol- and HCV/HBV-related HCC associated variants detailed in the current study analyses cohorts

**Previously known HCC disease associations**				**Discovery cohort (Current study)**			**Replication cohort 1 UK (Current study)**			**Replication cohort 2 Germany (Current study)**		
Study	Study type	Gene, Chrom.	SNP (Effect Allele)	AF in Ca|Co	Odds Ratio	P value	AF in Ca|Co	Odds Ratio	P value	AF in Ca|Co	Odds Ratio	P value
Trepo et al./ Stickel et al.	GWAS / CGS	*PNPLA3* (Chr 22)	rs738409 (G)	0.48|0.35	1.71 (1.49-1.96)	7.23×10^{-15}	0.49|0.30	2.20 (1.55-3.11)	6.31×10^{-6}	0.49|0.38	1.57 (0.97-2.56)	0.068
Trepo et al./ Stickel et al.	GWAS / CGS	*TM6SF2* (Chr 19)	rs58542926 (T)	0.15|0.08	1.94 (1.56-2.42)	2.81×10^{-9}	0.21|0.09	2.55 (1.65-3.95)	8.35×10^{-6}	0.13|0.11	1.21 (0.63-2.35)	0.565
Trepo et al.	GWAS	*WNT3A* (Chr 1)	rs708113 (T)	0.37|0.38	0.97 (0.85-1.11)	0.678	0.37|0.38	0.95 (0.70-1.29)	0.741	0.43|0.35	1.48 (0.89-2.47)	0.132
Stickel, Lutz, Buch et al.	CGS	*HSD17B13* (Chr 4)	rs72613567 (TA)	0.19|0.22	0.83 (0.71-0.97)	0.020	0.23|0.26	0.86 (0.61-1.21)	0.381	0.18|0.22	0.81 (0.47-1.41)	0.457
Innes et al.	CGS	*APOE* (Chr 19)	rs429358 (C)	0.09|0.12	0.74 (0.60-0.92)	6.35×10^{-3}	0.07|0.14	0.51 (0.28-0.93)	0.027	0.13|0.15	0.84 (0.42-1.70)	0.633
Innes et al.	CGS	*TM6SF2* (Chr 19)	rs187429064 (G)	0.02|0.01	1.82 (1.10-3.00)	0.019	0.08|0.02	3.21 (1.67-6.18)	4.79×10^{-4}	0.01|0.02	0.71 (0.05-9.78)	0.799
Miki et. al	GWAS (HCV)	*DEPDC5* (Chr 22)	rs1012068 (G)	0.27|0.24	1.15 (0.99-1.35)	0.069	0.27|0.23	1.36 (0.91-2.02)	0.134	0.28|0.28	1.00 (0.59-1.68)	1
Jiang et al.	GWAS (HBV)	*STAT4* (Chr 2)	rs7574865 (G)	0.77|0.78	0.97 (0.83-1.14)	0.730	0.76|0.77	0.88 (0.58-1.32)	0.529	0.76|0.81	0.74 (0.42-1.30)	0.316
Jiang et al.	GWAS (HBV)	*HLA-DQ* (Chr 6)	rs9275319 (A)	0.88|0.87	1.03 (0.83-1.27)	0.799	0.86|0.89	0.78 (0.43-1.43)	0.443	0.86|0.86	0.94 (0.49-1.80)	0.859

AF: Allele frequency, Ca: Cases (ArC with HCC), Co: Controls (ArC without HCC). Trepo et al.: Common genetic variation in alcohol-related HCC: a case-control genome-wide association study. The Lancet Oncology Dec 2021; Stickel, Lutz, Buch et al.: Genetic Variation in *HSD17B13* Reduces the Risk of Developing Cirrhosis and HCC in Alcohol Misusers. Hepatology Jul 2020; Innes et al.: The rs429358 locus in APOE is associated with HCC in patients with cirrhosis. Hepatology Communications Dec 2021; Stickel et al.: Genetic variants in PNPLA3 and TM6SF2 predispose to the development of HCC in individuals with ArC. Am J Gastroenterol. 2018 Oct; Miki et.al.: Variation in the DEPDC5 locus is associated with progression to HCC in chronic hepatitis C virus carriers. Nature Genetics Jul 2011.; Jiang et al.: Genetic variants in STAT4 and HLA-DQ genes confer risk of hepatitis B virus-related HCC. Nature Genetics Jan 2013.

Supplementary Table 8: Univariate analyses for association of *TERT* rs2242652 with alcohol-related cirrhosis and HCC in the whole cohort

Cohorts	*TERT* (rs2242652)	Comparative groups		Genotypic OR (95% CI), P value	Allelic OR (95% CI), P value	Cases / Controls (n)
		**HCC**	**ArC**			
Alcoholic-related cirrhosis and HCC **(HCC)**	G|G	919	1201			
	A|G	273	588	0.61 (0.51-0.72)	0.62 (0.53-0.71)	1214 | 1859
	A|A	22	70	0.41 (0.25-0.67)		
	MAF	0.131	0.196	2.32×10^{-10}	2.81×10^{-11}	
		**ArC**	**AM**			
Alcohol-related cirrhosis without HCC **(ArC)**	G|G	1201	981			
	A|G	588	479	1.00 (0.87-1.16)	0.99 (0.88-1.12)	1859| 1520
	A|A	70	60	0.95 (0.67-1.36)		
	MAF	0.196	0.197	0.963	0.899	
		**HCC**	**AM**			
Alcohol misusers **(AM)**	G|G	919	981			
	A|G	273	479	0.61 (0.51-0.72)	0.61 (0.53-0.71)	1214 | 1520
	A|A	22	60	0.39 (0.24-0.64)		
	MAF	0.131	0.197	6.38×10^{-10}	6.13×10^{-11}	

CI: confidence intervals, MAF: minor allele frequency, OR: odds ratio. Genotype counts reflect the rounded sum of imputed genotype probabilities for individuals in the sample. Genotypic odds ratios for heterozygous and homozygous carriership of the rare allele and allelic odds ratios were calculated from 2 × 2 tables on genotype counts. Significance was calculated as one degree of freedom Chi-squared test of allelic counts and 2 degree of freedom Chi-squared test of genotype counts.

Supplementary Table 9: Univariate analyses for association of *PNPLA3* rs738409 with alcohol-related cirrhosis and HCC in the whole cohort

Cohorts	*PNPLA3* (rs738409)	Comparative groups		Genotypic OR (95% CI), P value	Allelic OR (95% CI), P value	Cases/ Controls (n)
		**HCC**	**ArC**			
Alcoholic-related cirrhosis and HCC **(HCC)**	C|C	339	840			
	C|G	576	799	1.79 (1.51-2.11)	1.85 (1.67-2.06)	1214 | 1866
	G|G	229	227	3.26 (2.64-4.04)		
	MAF	0.484	0.336	3.28×10^{-28}	4.15×10^{-31}	
		**ArC**	**AM**			
Alcohol-related cirrhosis without HCC **(ArC)**	C|C	840	887			
	C|G	799	495	1.70 (1.47-1.97)	1.85 (1.65-2.06)	1866 | 1455
	G|G	227	63	3.80 (2.83-5.11)		
	MAF	0.336	0.215	1.99×10^{-25}	2.52×10^{-27}	
		**HCC**	**AM**			
	C|C	339	887			
Alcohol misusers **(AM)**	C|G	576	495	3.04 (2.56-3.62)	3.42 (3.04-3.85)	1214 | 1455
	G|G	229	63	12.42 (9.21-16.75)		
	MAF	0.484	0.215	6.21×10^{-85}	1.29×10^{-94}	

CI: confidence intervals, MAF: minor allele frequency, OR: odds ratio. Genotype counts reflect the rounded sum of imputed genotype probabilities for individuals in the sample. Genotypic odds ratios for heterozygous and homozygous carriership of the rare allele and allelic odds ratios were calculated from 2 × 2 tables on genotype counts. Significance was calculated as one degree of freedom Chi-squared test of allelic counts and 2 degree of freedom Chi-squared test of genotype counts.

Supplementary Table 10: Univariate analyses for association of *TM6SF2* rs58542926 with alcohol-related cirrhosis and HCC in the whole cohort

Cohorts	*TM6SF2* (rs58542926)	Comparative groups		Genotypic OR (95% CI), P value	Allelic OR (95% CI), P value	Cases/ Controls (n)
		**HCC**	**ArC**			
Alcoholic-related cirrhosis and HCC **(HCC)**	C|C	890	1545			
	C|T	285	300	1.65 (1.37-1.98)	1.74 (1.49-2.04)	1214 | 1866
	T|T	39	21	3.22 (1.88-5.52)		
	MAF	0.150	0.092	1.02×10^{-10}	3.14×10^{-12}	
		**ArC**	**AM**			
Alcohol-related cirrhosis without HCC **(ArC)**	C|C	1545	1283			
	C|T	300	167	1.49 (1.22-1.83)	1.56 (1.29-1.88)	1866 | 1455
	T|T	21	5	3.49 (1.31-9.28)		
	MAF	0.092	0.061	2.19×10^{-5}	3.44×10^{-6}	
		**HCC**	**AM**			
Alcohol misusers **(AM)**	C|C	890	1283			
	C|T	285	167	2.46 (2.00-3.03)	2.71 (2.25-3.28)	1214 | 1455
	T|T	39	5	11.24 (4.41-28.64)		
	MAF	0.150	0.061	5.09×10^{-24}	1.02×10^{-26}	

CI: confidence intervals, MAF: minor allele frequency, OR: odds ratio. Genotype counts reflect the rounded sum of imputed genotype probabilities for individuals in the sample. Genotypic odds ratios for heterozygous and homozygous carriership of the rare allele and allelic odds ratios were calculated from 2 × 2 tables on genotype counts. Significance was calculated as one degree of freedom Chi-squared test of allelic counts.

Supplementary Table 11: Finemapping, conditional analysis and expression analysis of the *TERT* Locus

GWAS	*TERT* Gene Variants Annotation				Discov.	Meta-analysis stage 1&2				MAF	Imputation		Conditional A.		LD	Expression		Leukocyte telomere length		
**TOP**	**SNP:ALT**	**Pos Chr 5**	**Type**	**Location**	**Pgwas**	**Pfix**	**ORfix**	**Prand**	**ORrand**	**Ca | Co**	**Type**	**Rsq**	**Pcon**	**R2Lead**	**Block**	**eQTL_P**	**Zscore**	**Beta**	**P_Telo**	**TOP**
	rs35535053:T	1252972	downstr.	DIST=315	0.396	0.49	0.83	0.69	0.86	0.016 | 0.018	Imp	0.74	0.918	0.016	B-1	0.71	0.37 (T)	-	-	
	rs2853690:A	1253744	3´UTR	EX=16/16	0.531	0.83	0.98	0.83	0.98	0.173 | 0.179	Imp	0.90	0.277	0.005	B-1	0.71	0.37 (A)	-0.0010	7.99E-08	
	rs35033501:T	1253918	synonym	EX=16/16	9.1E-3	0.02	1.67	0.02	1.67	0.035 | 0.021	Imp	0.85	0.023	0.003	B-1	0.44	0.77 (T)	0.0001	0.885	
	rs35719940:T	1254594	missense	EX=15/16	0.386	0.25	1.31	0.25	1.31	0.021 | 0.017	Imp	0.85	0.612	0.004	B-1	0.17	1.37 (T)	0.0021	8.17E-06	
	rs33954691:A	1255520	synonym	EX=14/16	0.084	0.05	1.24	0.05	1.24	0.103 | 0.088	Imp	0.88	0.155	0.008	B-1	5.0E-5	-4.05 (A)	0.0004	0.119	
	rs35387865:T	1255844	intronic	IN=13/15	0.280	0.31	1.33	0.31	1.33	0.017 | 0.013	Imp	0.86	0.448	0.004	B-1	0.87	0.17 (T)	0.0055	5.58E-16	
	rs2853687:A	1256585	intronic	IN=13/15	0.857	0.94	1.01	0.94	1.01	0.265 | 0.273	Imp	0.96	0.335	0.023	B-1	0.72	-0.35 (A)	-0.0009	5.81E-08	
	rs35041195:T	1257288	intronic	IN=13/15	0.801	0.55	0.94	0.55	0.94	0.119 | 0.128	Imp	0.90	0.573	0.002	B-1	0.96	0.05 (T)	-0.0018	7.79E-12	
	rs2736122:A	1257621	intronic	IN=13/15	0.389	0.62	0.97	0.62	0.97	0.261 | 0.280	Gen	0.99	0.133	0.014	B-1	0.60	-0.52 (A)	-0.0007	1.54E-05	
	rs144704378:T	1259489	intronic	IN=12/15	0.028	0.03	1.39	0.03	1.39	0.054 | 0.041	Imp	0.94	0.072	0.007	B-1	0.01	-2.44 (T)	0.0008	0.021	
	rs2736118:C	1260195	intronic	IN=12/15	0.530	0.60	0.96	0.60	0.96	0.262 | 0.278	Imp	0.98	0.204	0.014	B-1	0.73	-0.35 (C)	-0.0006	4.62E-05	
	rs34041736:T	1261051	intronic	IN=11/15	0.518	0.38	1.17	0.38	1.17	0.036 | 0.033	Imp	0.90	0.739	0.005	B-1	0.21	1.24 (T)	-0.0033	5.87E-15	
	rs11133715:A	1261052	intronic	IN=11/15	0.063	0.08	0.88	0.08	0.88	0.329 | 0.344	Imp	0.90	0.452	0.051	B-1	0.13	1.51 (A)	-	-	
	rs36077395:A	1261220	intronic	IN=11/15	0.679	0.53	1.18	0.53	1.18	0.016 | 0.015	Imp	0.87	0.854	0.002	B-1	0.25	1.14 (A)	-0.0012	0.020	
	rs34529095:C	1263408	intronic	IN=11/15	0.749	0.87	0.97	0.97	1.01	0.031 | 0.030	Imp	0.80	0.492	0.026	B-1	0.07	1.84 (C)	0.0011	0.014	
	rs2736115:T	1264068	intronic	IN=11/15	0.420	0.61	0.97	0.61	0.97	0.259 | 0.276	Imp	0.95	0.143	0.015	B-1	0.77	-0.29 (T)	-0.0008	1.16E-06	
	rs2853685:G	1264152	intronic	IN=11/15	0.463	0.68	0.97	0.68	0.97	0.270 | 0.285	Imp	0.94	0.152	0.017	B-1	0.80	-0.25 (G)	-0.0009	7.74E-09	
	rs2736114:T	1265204	intronic	IN=10/15	0.422	0.65	0.97	0.65	0.97	0.260 | 0.277	Imp	0.95	0.142	0.015	B-1	0.75	-0.32 (T)	-0.0008	3.69E-07	
	rs2736113:A	1265373	intronic	IN=10/15	0.376	0.60	0.96	0.60	0.96	0.259 | 0.277	Imp	0.95	0.120	0.015	B-1	0.77	-0.29 (A)	-0.0008	4.05E-07	
	rs2736111:A	1265935	intronic	IN=10/15	0.404	0.63	0.97	0.63	0.97	0.259 | 0.277	Imp	0.95	0.133	0.015	B-1	0.78	-0.28 (A)	-0.0008	8.65E-07	
	rs2853684:C	1266226	intronic	IN=10/15	0.515	0.75	0.98	0.75	0.98	0.262 | 0.278	Imp	0.95	0.182	0.016	B-1	0.76	-0.3 (C)	-0.0008	1.67E-06	
	rs2075786:G	1266310	intronic	IN=10/15	5.8E-3	5.7E-03	0.82	5.7E-03	0.82	0.623 | 0.659	Imp	0.88	0.061	0.035	B-1	0.49	-0.7 (A)	-	-	
	rs3891054:G	1267202	intronic	IN=9/15	0.374	0.27	1.11	0.27	1.11	0.147 | 0.146	Imp	0.87	0.974	0.025	B-2	N/A	N/A	-0.0014	2.16E-08	
	rs34194491:C	1267213	intronic	IN=9/15	0.024	0.02	1.61	0.02	1.61	0.033 | 0.022	Gen	1.00	0.051	0.004	B-2	0.01	-2.5 (C)	0.0010	0.042	
	rs4246742:A	1267356	intronic	IN=9/15	0.042	0.03	1.22	0.03	1.22	0.179 | 0.159	Imp	0.88	0.259	0.030	B-2	N/A	N/A	-	-	
	rs35812074:G	1267881	intronic	IN=9/15	0.218	0.18	1.42	0.18	1.42	0.020 | 0.015	Imp	0.75	0.379	0.004	B-2	N/A	N/A	0.0061	3.86E-25	
	rs114401494:T	1269161	intronic	IN=8/15	0.248	0.23	0.74	0.23	0.74	0.016 | 0.027	Imp	0.88	0.124	0.005	B-2	0.31	1.03 (T)	-	-	
	rs11742908:G	1270983	intronic	IN=8/15	5.0E-3	4.1E-03	0.77	4.1E-03	0.77	0.154 | 0.185	Imp	0.87	0.306	0.516	B-2	N/A	N/A	-	-	
	rs11133719:C	1271524	intronic	IN=7/15	0.172	0.11	0.86	0.11	0.86	0.832 | 0.849	Imp	0.84	0.631	0.030	B-2	0.02	-2.37 (T)	-	-	
	rs13172201:C	1271661	intronic	IN=7/15	4.9E-3	3.9E-03	1.25	3.9E-03	1.25	0.284 | 0.252	Imp	0.91	0.099	0.055	B-2	0.75	-0.31 (C)	-	-	

4	rs35517815:A	1274445	intronic	IN=6/15	1.7E-4	1.5E-04	5.11	1.5E-04	5.11	0.020 | 0.006	Imp	0.73	0.0004	0.002		0.37	0.9 (A)	-	-	
	rs4975605:A	1275528	intronic	IN=6/15	0.505	0.68	0.97	0.68	0.97	0.462 | 0.469	Imp	0.92	0.060	0.061		0.04	-2.04 (A)	-	-	
	rs145685051:G	1276736	intronic	IN=6/15	0.869	0.80	1.07	0.80	1.07	0.018 | 0.016	Imp	0.88	0.038	0.109		0.68	0.41 (G)	0.0033	3.74E-09	
6	rs56345976:A	1276873	intronic	IN=6/15	8.7E-4	7.2E-04	0.80	7.2E-04	0.80	0.569 | 0.624	Gen	0.99	0.070	0.107		0.002	-3.13 (G)	-	-	
	rs33961405:A	1277577	intronic	IN=6/15	0.682	0.51	1.05	0.51	1.05	0.533 | 0.534	Imp	0.93	0.100	0.150	B-3	0.74	-0.34 (A)	-	-	
	rs144020096:A	1278447	intronic	IN=6/15	0.116	0.06	0.54	0.06	0.54	0.008 | 0.015	Imp	0.95	0.740	0.066	B-3	0.43	-0.78 (A)	-0.0008	0.278	
	rs2075785:T	1278584	intronic	IN=6/15	0.199	0.07	1.18	0.07	1.18	0.135 | 0.128	Imp	0.94	0.691	0.029	B-3	0.37	0.90 (T)	-0.0034	6.56E-48	
	rs35241335:G	1279224	intronic	IN=5/15	0.260	0.08	1.23	0.08	1.23	0.077 | 0.075	Imp	0.94	0.697	0.016	B-3	0.55	0.59 (G)	-0.0036	4.40E-29	
**Top 2**	**rs10069690:T**	**1279790**	**intronic**	**IN=4/15**	**5.7E-6**	**5.2E-08**	**0.66**	**5.2E-08**	**0.66**	**0.183 | 0.242**	**Gen**	**1.00**	**0.485**	**0.696**	**B-3**	**0.003**	**2.96 (T)**	**0.0031**	**4.08E-84**	
9	rs10054203:C	1279964	intronic	IN=4/15	3.4E-3	7.6E-04	0.79	0.03	0.76	0.352 | 0.392	Imp	0.95	0.967	0.328	B-3	0.04	2.01 (C)	0.0036	8.83E-133	10
**Top1**	**rs2242652:A**	**1280028**	**intronic**	**IN=4/15**	**7.9E-7**	**6.4E-09**	**0.61**	**6.4E-09**	**0.61**	**0.133 | 0.191**	**Gen**	**1.00**	**NA**	**1.000**	**B-3**	**1.4E-5**	**4.35 (A)**	**0.0026**	**2.12E-44**	
	rs7734992:C	1280128	intronic	IN=4/15	0.019	9.3E-04	0.81	5.9E-03	0.78	0.367 | 0.401	Imp	0.96	0.555	0.315	B-3	0.04	2.03 (C)	0.0041	2.15E-168	5
	rs13167280:A	1280477	intronic	IN=3/15	0.099	0.12	1.15	0.12	1.15	0.158 | 0.139	Imp	0.95	0.482	0.033	B-3	0.26	-1.13 (A)	0.0026	3.28E-32	
10	rs4975538:C	1280830	intronic	IN=3/15	3.9E-3	2.0E-04	0.78	2.0E-04	0.78	0.316 | 0.356	Imp	0.95	0.826	0.383	B-3	0.01	2.57 (C)	0.0039	5.02E-145	8
	**rs7726159:A**	**1282319**	**intronic**	**IN=3/15**	**5.2E-3**	**1.6E-04**	**0.78**	**9.2E-03**	**0.72**	**0.292 | 0.329**	**Imp**	**0.99**	**0.795**	**0.354**	**B-3**	**0.02**	**2.32 (A)**	**0.0048**	**1.16E-219**	**Top2**
8	rs7725218:A	1282414	intronic	IN=3/15	3.2E-3	8.1E-05	0.77	4.4E-03	0.73	0.302 | 0.342	Gen	1.00	0.997	0.334	B-3	0.05	1.96 (A)	0.0045	2.77E-198	4
	rs7713218:G	1283312	intronic	IN=2/15	0.018	3.7E-03	1.20	3.7E-03	1.20	0.554 | 0.523	Imp	0.95	0.830	0.185	B-3	0.004	2.87 (A)	0.0033	5.31E-114	
**Top3**	**rs72709458:T**	**1283755**	**intronic**	**IN=2/15**	**3.9E-5**	**2.9E-07**	**0.66**	**1.1E-03**	**0.58**	**0.156 | 0.208**	**Imp**	**0.97**	**0.771**	**0.749**	**B-3**	**3.4E-5**	**4.14 (T)**	**0.0034**	**1.10E-81**	
	rs6420019:C	1283841	intronic	IN=2/15	0.656	0.32	0.91	0.32	0.91	0.845 | 0.852	Imp	0.89	0.595	0.034	B-3	0.57	-0.57 (A)	-0.0019	4.95E-20	
	rs6420020:C	1284046	intronic	IN=2/15	0.651	0.31	0.91	0.32	0.90	0.845 | 0.852	Imp	0.89	0.600	0.034	B-3	0.62	-0.5 (T)	-0.0019	3.03E-20	
	**rs4449583:T**	**1284135**	**intronic**	**IN=2/15**	**5.3E-3**	**2.2E-04**	**0.78**	**1.5E-03**	**0.77**	**0.291 | 0.329**	**Imp**	**0.99**	**0.796**	**0.353**	**B-3**	**0.02**	**2.27 (T)**	**0.0048**	**9.93E-215**	**Top3**
	rs35029535:T	1284976	intronic	IN=2/15	0.165	0.13	1.10	0.13	1.10	0.406 | 0.377	Imp	0.95	0.687	0.125	B-3	0.02	-2.31 (T)	-0.0017	9.77E-30	
	**rs7705526:A**	**1285974**	**intronic**	**IN=2/15**	**0.046**	**3.3E-03**	**0.82**	**6.6E-03**	**0.81**	**0.295 | 0.322**	**Gen**	**1.00**	**0.647**	**0.210**	**B-3**	**0.27**	**1.1 (A)**	**0.0052**	**1.64E-245**	**Top1**
	rs2736100:A	1286516	intronic	IN=2/15	0.333	0.07	1.12	0.12	1.18	0.536 | 0.528	Gen	1.00	0.230	0.162	B-3	0.11	-1.61 (A)	-0.0039	1.47E-166	6
	rs2853677:A	1287194	intronic	IN=2/15	0.022	0.01	1.18	0.01	1.18	0.596 | 0.571	Gen	0.99	0.349	0.077	B-4	0.11	1.58 (G)	0.0036	1.31E-139	9
	rs35838177:T	1287290	intronic	IN=2/15	0.554	0.43	1.14	0.43	1.14	0.04 | 0.031	Gen	0.99	0.492	0.000	B-4	0.87	-0.17 (T)	0.0001	0.891	
	rs2736099:G	1287340	intronic	IN=2/15	0.504	0.27	1.08	0.35	1.11	0.661 | 0.655	Imp	0.94	0.686	0.047	B-4	0.78	0.28 (A)	0.0040	8.35E-146	7
5	rs7710703:C	1287505	intronic	IN=2/15	6.6E-4	7.9E-05	1.47	0.02	1.57	0.888 | 0.859	Imp	0.89	0.146	0.164	B-5	0.002	3.16 (T)	0.0015	3.16E-12	
	rs2853676:C	1288547	intronic	IN=2/15	0.168	0.04	1.15	0.04	1.15	0.753 | 0.740	Gen	1.00	0.642	0.132	B-5	0.31	1.02 (T)	0.0020	1.71E-37	
	rs34677523:A	1288883	intronic	IN=2/15	0.165	0.40	0.79	0.92	1.08	0.012 | 0.020	Imp	0.87	0.107	0.001	B-5	N/A	N/A	-	-	
	rs72709460:A	1289220	intronic	IN=2/15	0.571	0.57	1.22	0.57	1.22	0.012 | 0.010	Imp	0.77	0.696	0.001	B-5	0.39	-0.85 (A)	-	-	
	rs115451758:A	1289277	intronic	IN=2/15	0.223	0.19	1.61	0.19	1.61	0.012 | 0.007	Imp	0.94	0.302	0.001	B-5	0.005	-2.79 (A)	-	-	
	rs796501027:T	1291530	intronic	IN=2/15	0.052	0.02	1.41	0.71	1.24	0.127 | 0.113	Imp	0.43	0.192	0.008	B-5	N/A	N/A	-	-	

	rs56023411:T	1291740	intronic	IN=2/15	7.0E-3	3.2E-03	1.32	0.13	1.38	0.804 | 0.778	Imp	0.77	0.632	0.226	B-5	0.004	2.84 (G)	-	-
	rs71595003:A	1292118	intronic	IN=2/15	0.031	0.03	1.51	0.07	1.50	0.035 | 0.022	Gen	0.99	0.073	0.005	B-5	0.63	0.48 (A)	0.0000	0.982
	rs35334674:A	1292299	intronic	IN=2/15	0.051	0.06	0.70	0.80	0.88	0.030 | 0.038	Imp	0.85	0.030	0.002	B-5	0.89	0.14 (A)	0.0010	0.014
	rs114616103:T	1292958	intronic	IN=2/15	0.294	0.09	0.72	0.10	0.29	0.025 | 0.031	Gen	1.00	0.571	0.011	B-5	0.19	-1.31 (T)	-0.0039	8.23E-21
	rs2853672:A	1292983	intronic	IN=2/15	0.044	0.01	1.17	0.15	1.21	0.527 | 0.507	Gen	1.00	0.441	0.062	B-5	0.69	-0.4 (A)	-0.0030	2.91E-99
	rs79662648:G	1293389	intronic	IN=2/15	0.392	0.19	1.24	0.19	1.24	0.040 | 0.031	Imp	0.95	0.498	0.001	B-5	N/A	N/A	-0.0009	0.027
	rs2736098:T	1294086	synonym	EX=2/16	0.398	0.26	1.08	0.40	1.10	0.271 | 0.253	Imp	0.98	0.936	0.036	B-5	0.36	-0.92 (T)	0.0026	1.48E-58
	rs61748181:T	1294166	missense	EX=2/16	0.294	0.09	0.72	0.10	0.29	0.025 | 0.031	Gen	1.00	0.571	0.011	B-5	0.47	-0.72 (T)	-0.0040	6.40E-21
	rs2853669:G	1295349	upstream	DIST=187	0.246	0.11	1.11	0.18	1.12	0.315 | 0.289	Gen	1.00	0.830	0.037	B-5	0.06	-1.88 (G)	-	-
	rs35226131:T	1295373	upstream	DIST=211	0.294	0.09	0.72	0.10	0.29	0.025 | 0.031	Gen	1.00	0.571	0.011	B-5	0.26	-1.13 (T)	-	-
	rs35161420:G	1295452	upstream	DIST=290	0.296	0.09	0.71	0.10	0.29	0.025 | 0.031	Imp	0.99	0.575	0.011	B-5	0.23	-1.19 (G)	-	-
	rs33958877:T	1295682	upstream	DIST=520	0.328	0.11	0.73	0.11	0.29	0.025 | 0.031	Gen	1.00	0.616	0.011	B-5	0.16	-1.42 (T)	-	-
	rs34768248:A	1295716	upstream	DIST=554	0.017	0.02	0.50	0.02	0.50	0.008 | 0.015	Gen	1.00	0.099	0.017	B-5	0.79	0.26 (A)	-	-
	rs34685900:C	1295803	upstream	DIST=641	7.5E-3	0.01	0.51	0.01	0.51	0.009 | 0.018	Gen	1.00	0.042	0.014	B-5	0.71	0.37 (C)	-	-
7	rs7712562:G	1296072	upstream	DIST=910	9.1E-4	6.9E-05	1.45	0.00	1.46	0.878 | 0.843	Imp	0.96	0.484	0.302	B-5	8.5E-5	3.93 (A)	-	-
	rs2735940:G	1296486	upstream	DIST=1324	0.006	0.01	1.17	0.14	1.21	0.529 | 0.507	Gen	1.00	0.409	0.061	B-5	0.65	1.54 (C)	-	-
	rs33977403:T	1296727	upstream	DIST=1565	0.931	0.67	1.12	0.67	1.12	0.013 | 0.014	Gen	0.99	0.748	0.002	B-5	0.25	-1.91 (T)	-	-

LD-Block information at the TERT locus, *cise*QTL Expression, UK Biobank Leukocyte telomere length data and conditional analysis on the lead SNP rs2242652 in the primary GWAS samples. Abbreviations: Top 10 GWAS variants ranked by P value in the discovery GWAS, top 3 in bold print.; SNP:ALT: SNP with alternative allele (reference allele for odds ratio); POS: genomic position on Chr 5; IN: intron, EX: exon; DIST:distance to *TERT* gene; Pgwas: Association P value in discovery; Pfix: fixed-effects meta-analysis and Prand random-effects meta-analysis association P value in the combined analysis of stage 1 discovery and stage 2 replication; ORfix/rand (CI95): Allelic odds ratio with 95% confidence interval; MAF Ca | Co minor allele freq in cases (HCC) and controls (CIRR); Imputation information: Rsq (imputation r2 info score); Annotated LD-Blocks are clusters of strong pairwise LD SNPs and reflect the LD pattern in the discovery GWAS cohort. Conditional Analysis: Pcon: Logistic regression P value of the respective variant conditioned on the allele dosage of the lead variant rs2242652 shown in bold print. Upon conditional analysis, the variant rs2242652 captures the association information in LD block B3 and of the entire *TERT* locus; R2Lead: R2 value of the respective variant in relation to the lead variant rs2242652:A in the discovery sample; Block: LD block assignment based on R2 value; Expression: CiseQTL P values for cis-eQTL effect on TERT expression in blood, from eQTLGen (https://eqtlgen.org/). Zscore of effect strength and effect direction of assed allele in brackets. Leukocyte telomere length information obtained from UK Biobank: P_Telo/Beta: P value and beta of association of the respective variant with leukocyte telomere length; TOP: Top 10 variants ranked by P value in the leukocyte telomere length analysis, top 3 in bold print.

Supplementary Table 12: *TERT* Locus - conditional analysis in the primary discovery samples

**Conditioned on rs2242652**	**OR (CI 95%)**	**P value**
rs2242652	NA	NA
rs10069690	OR = 0.90 (0.67-1.21)	0.485
rs72709458	OR = 1.05 (0.75-1.47)	
**Conditioned on rs10069690**		
rs2242652	OR = 0.70 (0.50-0.98)	**0.036**
rs10069690	NA	NA
rs72709458	OR = 0.91 (0.67-1.24)	

Conditional analysis of the top 3 associated variants at the *TERT* locus, located in LD block B3. Upon conditional analysis, the variant rs2242652 captures the association information at the locus, as shown in Supplementary Table 12**.** The variant rs2242652 remains significantly associated with HCC after conditioning on variant rs10069690.

Supplementary Table 13: Summary of data set used for additional replication of *TERT* variants

		Characteristics				[Minor allele] frequency (%)			
**Cohort**	**Phenotypes (ICD10 codes)**	HCC (n,%)	N	Age* (mean)	% male	*TERT* [A] rs2242652	*TERT* [T] rs10069690	*PNPLA3*[G] rs738409	*TM6SF2*[T] rs58542926
Stop-HCV	Cases: C22.0 Liver cell carcinoma (HCC) in HCV-related cirrhosis	169 (100)	169	60	73	0.151	0.210	0.281	0.080
	Controls: HCV-related cirrhosis without HCC		890	56	77	0.197	0.270	0.253	0.090
Trépo *et al*	Cases: C22.0 HCC in ALD (with advanced fibrosis/cirrhosis)	775 (100)	775	65	90	n/a	n/a	0.43	0.13
	Controls: alcohol-related liver disease advanced fibrosis/cirrhosis		1332	56	72	n/a	n/a	0.33	0.08
Zhang *et al*.	Cases: Chinese patients with C22.0 HCC (patients with HCV were excluded)	473 (100)	473	56	83	0.133	0.135	n/a	n/a
	Controls: non-cancer individuals from the Physical Examination Center of Haikou People's Hospital		564	54	60	0.179	0.171	n/a	n/a
Dong *et al*.	Cases: male Chinese patients with C22.0 HCC (hepatitis-induced)	181 (100)	181	n/a	100	n/a	0.072	n/a	n/a
	Controls: male, non-cancer individuals from General Hospital of PLA, Beijing		106	n/a	100	n/a	0.184	n/a	n/a
FinnGen	Cases: C22 malignant neoplasm of liver and intrahepatic bile duct	265 (60)	442	71	82	0.193	0.253	0.320	0.105
	Controls: FinnGen biobank participants without a diagnosis of primary liver cancer (excluding all other cancers)		204,070	63	43	0.230	0.296	0.227	0.064
UKBB**	Cases: C22 malignant neoplasm of liver and intrahepatic bile duct	383 (44)	874	62	78	0.169	0.226	0.264	0.109
	Controls: UKB participants who did not meet the above definition of a case		348,465	58	47	0.190	0.258	0.216	0.076
UKBB*	Cases: C22.0 Liver cell carcinoma (HCC)	383 (100)	383	62	77	0.159	0.202	0.325	0.142
	Controls: UKB participants who did not meet the above definition of a case		348,956	58	47	0.190	0.258	0.216	0.076
BBJ Japan***	Cases: C22.0 Liver cell carcinoma (HCC)	1866 (100)	1866	68	74	n/a	n/a | 0.183	0.495	0.085
	Controls: BBJ participants who did not meet the above definition of a case			62	50	n/a	n/a | 0.209	0.458	0.078

n/a: data not available; *Mean age at first event (years) ** As UKB data were incorporated into our discovery analysis, further interrogation of liver cancer phenotypes from UKB does not constitute independent validation. *** Variants rs2242652 and rs10069690 were not available in BioBank Japan summary GWAS data from Ishigaki et al.(67) (PMID: 32514122), publicly available from http://jenger.riken.jp/en/result; maf data is reported for proxy variants rs72709458 (r2=0.973 between rs72709458 and rs2242652, both variants are in high LD).

Supplementary Table 14: Information for association of *TERT* variants rs2242652, rs10069690 with other cancer from the NHGRI-EBI Catalog of human genome-wide association studies

Reported cancer trait NHGRI-EBI -GWAS Catalog	Variant	P-value	Effect allele	Association	OR	CI	First Author	PubMed ID
Prostate cancer	rs2242652	8 x 10-55	A - minor allele	protective	0.88	[0.86-0.89]	Conti DV	33398198
Prostate cancer	rs2242652	4 x 10-52	A - minor allele	protective	0.85	[0.84-0.87]	Schumacher	29892016
Prostate cancer	rs2242652	3 x 10-24	A - minor allele	protective	0.87	[0.84-0.90]	Kote-Jarai Z	21743467
Prostate cancer	rs2242652	1 x 10-15	A - minor allele	protective	0.86	NR	Emami NC	33293427
Prostate cancer	rs2242652	5 x 10-12	A - minor allele	protective	0.85	NR	Rashkin SR	32887889
Prostate cancer	rs2242652	8 x 10-6	A - minor allele	protective	0.87	NR	Takata R	31562322
Uterine leiomyoma	rs2242652	2 x 10-14	A - minor allele	protective	0.90	[0.88-0.92]	Sakai K	31988393
Multiple myeloma	rs2242652	1 x 10-3	A - minor allele	protective	0.81	[0.72-0.92]	Campa D	25066524
Breast cancer (ER negative)	rs2242652	2 x 10-14	A - minor allele	risk increasing	1.18	[1.13-1.23]	Couch FJ	27117709
Gastric cancer	rs2242652	4 x 10-4	A - minor allele	risk increasing	1.46	[1.28-2.92]	Lili M	32502020
Skin cancer	rs2242652	4 x 10-3	A - minor allele	risk increasing	1.50	[1.14-1.98]	Nan H	21116649
Esophageal cancer	rs2242652	1 x 10-3	A - minor allele	risk increasing	1.48	[1.17-1.89]	Wu Y	28060765
Lung cancer	rs2242652	0.04	A - minor allele	risk increasing	1.47	[1.02-2.13]	Gao L	25254308
Breast cancer (ER negative)	rs10069690	2 x 10-35	T - minor allele	risk increasing	1.18	[1.15-1.21]	Milne RL	29058716
Breast cancer	rs10069690	2 x 10-20	T - minor allele	risk increasing	1.06	[1.05-1.08]	Shu X	32139696
Breast cancer	rs10069690	8 x 10-17	T - minor allele	risk increasing	1.06	[1.04-1.08]	Michailidou K	29059683
Breast cancer	rs10069690	5 x 10-12	T - minor allele	risk increasing	1.15	[1.11-1.20]	Garcia-Closas	23535733
Breast cancer	rs10069690	1 x 10-10	T - minor allele	risk increasing	1.18	[1.13-1.25]	Haiman CA	22037553
Breast cancer	rs10069690	7 x 10-9	T - minor allele	risk increasing	1.06	[1.04-1.09]	Michailidou K	23535729
Breast cancer (ER negative)	rs10069690	1 x 10-7	T - minor allele	risk increasing	1.24	[1.14-1.34]	Purrington KS	24325915
Glioblastoma	rs10069690	8 x 10-74	T - minor allele	risk increasing	1.61	[1.53-1.69]	Melin BS	28346443
Glioblastoma	rs10069690	3 x 10-35	T - minor allele	risk increasing	1.64	[1.52-1.78]	Ostrom QT	29743610
Glioma	rs10069690	3 x 10-66	T - minor allele	risk increasing	1.45	[1.39-1.51]	Melin BS	28346443
Glioma	rs10069690	8 x 10-31	T - minor allele	risk increasing	1.49	[1.39-1.60]	Ostrom QT	29743610
Non-glioblastoma glioma	rs10069690	1 x 10-16	T - minor allele	risk increasing	1.27	[1.20-1.34]	Melin BS	28346443
Non-glioblastoma glioma	rs10069690	8 x 10-7	T - minor allele	risk increasing	1.25	[1.15-1.37]	Kinnersley B	26424050
Serous ovarian cancer	rs10069690	1 x 10-9	T - minor allele	risk increasing	1.22	[1.14-1.29]	Phelan CM	28346442
Epithelial ovarian cancer	rs10069690	9 x 10-9	T - minor allele	risk increasing	1.14	[1.10-1.19]	Kuchenbaecker	25581431
Epithelial ovarian cancer	rs10069690	3 x 10-8	T - minor allele	risk increasing	1.09	[1.06-1.12]	Phelan CM	28346442
Thyroid cancer	rs10069690	3 x 10-7	T - minor allele	risk increasing	1.20	[1.12-1.29]	Gudmundsson J	28195142

Supplementary Table 15: Association between the number of risk alleles in *PNPLA3*, *TM6SF2* and *TERT* and alcohol-related HCC

**unweighted genetic risk score**	Number of risk alleles (reference: 0-2 risk alleles) *PNPLA3*:rs738409:G; *TM6SF2*:rs58542926:T; *TERT*:rs2242652:G			
**Discovery (n=1910)**	**3-4 risk alleles**		**5-6 risk alleles**	
Adjustments	OR (CI95%)	P value	OR (CI95%)	P value
unadjusted	2.12 (1.76-2.56)	4.88×10^{-15}	5.24 (2.82-9.77)	1.76×10^{-07}
pc, sex, age	2.27 (1.83-2.82)	1.36×10^{-15}	4.97 (2.50-9.91)	5.01×10^{-06}
**Validation UK (n=860)**	**3-4 risk alleles**		**5-6 risk alleles**	
unadjusted	3.25 (1.84-5.73)	4.60×10^{-05}	17.78 (6.38-49.57)	3.78×10^{-08}
pc, sex, age	3.35 (1.84-6.08)	7.60×10^{-05}	16.13 (5.07-51.31)	2.00×10^{-06}
**Validation Germany (n=238)**	**3-4 risk alleles**		**5-6 risk alleles**	
unadjusted	2.28 (1.08-4.83)	0.031	7.67 (1.69-34.73)	8.23×10^{-03}
pc, sex, age	2.30 (1.03-5.15)	0.031	8.58 (1.62-45.49)	0.012

Supplementary Table 16: Analysis of factors associated with HCC in patients with alcoholic cirrhosis with liver fat content (FFD%) and leukocyte telomere length in the UK Biobank

		Liver fat content			Leukocyte telomere length			Association with HCC		
Variable	SNP	N	Adjusted Beta (se) [a,b]	Significance (*P*) [b]	N	Adjusted Beta [a,b]	Significance (*P*) [b]	GWAS *P*-value	Per-allele OR (95%CI)	MAF Cases (HCC) / Controls (CIRR)
PNPLA3	rs738409:G	8,315	0.2204	3.39×10^{-61}	471,172	-0.00013	0.458	7.23×10^{-15}	1.71 (1.49-1.96)	0.48 / 0.35
TM6SF2	rs58542926:T	8,315	0.2904	5.94×10^{-45}	62,296	-0.00053	0.475	2.81×10^{-9}	1.94 (1.56-2.42)	0.15 / 0.08
TERT	rs2242652:A	8,319	0.0209	0.1442	458,714	0.00256	2.12×10^{-44}	7.87×10^{-7}	0.64 (0.53-0.76)	0.13 / 0.19
TERT	rs10069690:T			N/A	471,172	0.00313	4.08×10^{-84}	5.73×10^{-6}	0.69 (0.58-0.81)	0.183 / 0.242
TERT	rs72709458:T			N/A	454,347	0.00344	1.10×10^{-81}	3.92×10^{-5}	0.70 (0.59-0.83)	0.156 / 0.208
TERT	rs7726159:A			N/A	471,172	0.00476	1.16×10^{-219}	5.20×10^{-3}	0.81 (0.70-0.94)	0.292 / 0.329

SNP: single nucleotide polymorphism, N/A: not analyzed

[a] adjusted for age, gender, principal component 1 to 10.

[b] derived from UK Biobank analysis of >450.000 individuals of European decent

Supplementary Table 17: Estimates of the proportion of phenotypic variance explained by additive genome-wide significant SNPs for HCC (GWAS discovery cohort)

Discovery GWAS cohort n = 1910		*total variance explained by all SNPs*				*total variance explained by replicated risk variants / remaining variants*						
		all GWAS-SNPs [a] n = 7,585,576				*GWAS* SNPs [b] n = 7,583,550		***PNPLA3* / *TM6SF2* / *TERT* SNPs** [b] n = 2026 (LD region variants)				
Genetic variance component		Sum of V(G1+G2) / Vp				V(G1) / Vp		V(G2) / Vp				
		observed scale	disease prevalence 1% *	disease prevalence 2.5% *	LRT	observed scale	LRT	observed scale	disease prevalence 1% *	disease prevalence 2.5% *	LRT	*Proportion of variance explained by replicated risk variants*
Method	environmental variance component	h^2 (se) [a]	h^2 (se) [a]	h^2 (se) [a]	*P* [a]	h^2 (se) [b]	*P* [b]	h^2 (se) [b]	h^2 (se) [b]	h^2 (se) [b]	*P* [b]	% of total h^2 [c]
GCTA-GREML	15 PCs [e]	0.296 (0.181)	0.204 (0.107)	0.257 (0.135)	5.55×10^{-17}	0.221 (0.181)	0.108	0.075 (0.022)	0.042 (0.013)	0.053 (0.016)	1.32×10^{-17}	25.5%
GCTA-GREML	sex, age, 15 PCs [g]	0.243 (0.185)	0.176 (0.107)	0.222 (0.136)	3.16×10^{-13}	0.188 (0.185)	0.152	0.054 (0.018)	0.030 (0.010)	0.038 (0.013)	3.16×10^{-14}	22.2%

[a] GCTA-GREML estimate of the phenotypic variance (h^2) explained by all genotyped and imputed genome-wide SNPs in the discovery cohort (termed the SNP heritability), including the environmental variance component V(e) and the residual variance Vp.

[b] GCTA-GREML estimate of the phenotypic variance (h^2) explained by HCC associated genome-wide significant SNPs (V(G2) locating to the *PNPLA3* / *TM6SF2* / *TERT* associated linkage disequilibrium region (LD region) and remaining GWAS variants (V(G1) outside these LD regions.

[c] Percentage of SNP heritability due to *PNPLA3* / *TM6SF2* / *TERT* variants calculated by $\frac{h2(\mathrm{vc1})}{h2(\mathrm{vc0})+h2(\mathrm{vc1})}$.

[d] GCTA-GREML variance components G2 estimate (h^2) after adjustment for lead variants rs738409 in *PNPLA3* / rs58542926 in *TM6SF2* / rs2242652 in *TERT*.

[e] Likelihood model adjustments for top 15 principal components of genetic ancestry (environmental variance component).

[g] Likelihood model adjusted for sex, age and top 15 principal components of genetic ancestry (environmental variance component).

* Transformed h^2 estimates from heritability on the observed scale (GWAS cohort) to heritability on the liability scale (population) assuming HCC prevalence estimates of 1%-2.5% in patients with alcohol-related liver disease.

Abbreviations: V(G1) = genetic variance component 1; V(G2) = genetic variance component 2; (Vp) residual variance; h^2, SNP heritability; PC, principal components; se, standard error; LRT, likelihood ratio test; P, likelihood ratio test significance of the predicted change in log likelihood.
